# Supplementary material for: Experiences of and response to the COVID-19 pandemic at private retail pharmacies in Kenya: a mixed-methods study
Source: BMJ Open. 2022 Jun 28;12(6):e058688. doi: 10.1136/bmjopen-2021-058688 (PMC9240447; doi:10.1136/bmjopen-2021-058688)
Supplement: Supplementary data [file bmjopen-2021-058688supp002.pdf]

Supplementary Table I: Characteristics of participating pharmacies and respondents

A. Characteristics of participating pharmacies

Characteristic	Nairobi (n=38)		Mombasa (n=36)		Kisumu (n=34)		Total (n=108) ^	
	n	%	n	%	n	%	n	%
**Setting**								
Urban commercial centre	23	61%	29	81%	12	35%	64	59%
Urban residential area	13	34%	4	11%	6	18%	23	21%
Urban informal settlement	2	5%	3	8%	2	6%	7	6%
Rural town/ shopping centre	0	0%	0	0%	14	41%*	14	13%
**Years of operation**								
Number of years in operation (median [range])	5	1-19	4	1-42	5	1-11	5	1-42
**Physical facilities**								
Total number of rooms (Median [range])	2	1-10	2	1-6	2	1-8	1	1-10
Consultation room	14	37%	10	28%	20	59%*	44	41%
On-site lab	5	13%	1	3%	6	18%	12	11%
**Information systems**								
Computerized stock system	29	81%*	19	53%	23	68%	71	67%
Individual medical records[1]	9	25%	2	6%	7	21%	18	17%
**Chain network membership[2]**								
Part of a chain network	9	24%*	3	8%	1	3%	13	12%
Number of branches nationwide (Median [range])	1	1-41	1	1-41	1	1-9	1	1-41
**Branding[3]**								
None	33	87%	29	81%	32	94%	94	87%
Green Cross	2	5%	2	6%	0	0%	4	4%
Pharmnet (blue cross)	0	0%	3	8%	1	3%	4	4%
Corporate	3	8%	2	6%	1	3%	6	6%
**Distance to the nearest clinic**								
Within the pharmacy	0	0%	0	0%	2	6%	2	2%
< 100 meters	17	45%	13	36%	9	26%	39	36%
< 1km	19	50%	16	44%	21	62%	56	52%
< 5km	2	5%	7	19%	2	6%	11	10%
**Distance to the nearest HIV testing site**								
< 100 meters	14	37%	12	33%	11	32%	37	34%
< 1km	18	47%	16	44%	21	62%	55	51%
< 5km	4	11%	7	19%	2	6%	13	12%
>5km	1	3%	0	0%	0	0%	1	1%
Not sure	1	3%	1	3%	0	0%	2	2%
**Service providers**								
Total number of service providers (Median [range])	3*	1-16	2	1-9	2	1-4	2	1-16
At least one pharmacy degree holder	8	21%	7	19%	3	9%	18	17%

Characteristic	Nairobi (n=38)		Mombasa (n=36)		Kisumu (n=34)		Total (n=108) ^	
	n	%	n	%	n	%	n	%
At least one pharmacy diploma holder	36	95%	35	97%	34	100%	105	97%
All staff belong in a professional association	24	63%	19	53%	18	53%	61	56%
**Management**								
Written job descriptions	21	55%	12	33%	14	41%	47	44%
Regular staff meetings[4]	21	55%	16	44%	19	56%	56	52%
**Opening hours**								
24 hours daily, 7 days a week	4	11%	1	3%	1	3%	6	6%
At least seven days a week	23	61%	28	78%	25	74%	76	70%
At least 6 days a week	38	100%	34	94%	34	100%	106	98%
**Work load**								
Walk-in clients per day (Median [range])	55	15-300	50	20-200	40	20-200	50	15-300
Online clients per day (Median [range])	1	0-20	0	0-20	0	0-10	0	0-20
**Health promotion services**								
Over-the-counter STI treatment	29	76%	29	81%	30	88%	88	81%
Weight management	12	32%	6	17%	10	29%	28	26%
Smoking cessation	4	11%	1	3%	1	3%	6	6%
Vaccination	0	0%	1	3%	3	9%	4	4%
**Screening services**								
Blood pressure measurement	26	68%	26	72%	25	74%	77	71%
Blood sugar testing	19	50%	25	69%	20	59%	64	59%
HIV self-testing	21	55%	22	61%	20	59%	63	58%
Malaria testing	9	24%	19	53%	27	79%*	55	51%
In-pharmacy pregnancy testing	9	24%	9	25%	18	53%*	36	33%
Other screening services[5]	1	3%	0	0%	5	15%*	6	6%

^ Of 195 target pharmacies that had participated in the previous PHP study, 108 (55%) participated in the current survey. Of 87 that did not participate: 55 (63%) failed to complete the questionnaire after three reminders, 15 (17%) reported that the initial respondent had left and no one else was willing to participate, 8 (9%) were unreachable, 3 (3%) reported that the management did not approve participation, 2 (1%) were undergoing transition, 1 (1%) was closed, and 3 (3%) had other reasons (no access to smartphone or computer, preferred face-to-face interview, sold the pharmacy).

** Indicates significant difference between counties (p<0.05), based on chi square test or analysis of variance for means*

[1] An individual medical record is a document detailing the medications an individual patient is on, indications and drug allergies; may also include other clinical information.

[2] Chain network was defined as 6 or more branches nationwide, derived from chain size distribution pattern; 79 (73%) pharmacies were single branch, 16 (15%) were part of a 2-5 branch network, and 13 (12%) were part of a chain network

[3] Green Cross is a professional network of pharmacies owned by pharmacists; Pharmnet is for pharmaceutical technologists

[4] Excludes 18 pharmacies that responded "not applicable" since they have only one staff member

[5] Other screening services included; typhoid testing (n=4) and tuberculosis screening (n=2)

STI: Sexually transmitted infection

B. Characteristics of respondents

Characteristic	Nairobi (N=38)		Mombasa (N=36)		Kisumu (N=34)		Total (N=108)	
	**n**	**%**	**n**	**%**	**n**	**%**	**n**	**%**
**Gender**								
Female	12	32%	13	36%	16	47%	41	38%
Male	26	68%	23	64%	18	53%	67	62%
**Age**								
20 – 29 years	12	32%	12	33%	14	41%	38	35%
30 – 39 years	19	50%	18	50%	16	47%	53	49%
40 – 44 years	5	13%	4	11%	2	6%	11	10%
45 years and over	2	5%	2	6%	2	6%	6	6%
**Designation (role)**								
Owner	9	24%	12	33%	12	35%	33	31%
In-charge/ Superintendent	11	29%	13	36%	8	24%	32	30%
Staff[1]	18	47%	11	31%	14	41%	43	40%
**Highest level of education[2]**								
Non-health qualifications	0	0%	2	6%	1	3%	3	3%
Health-related certificate/ diploma/ bachelor's degree	2	5%	0	0%	2	6%	4	4%
Pharmacy certificate	2	5%	0	0%	1	3%	3	3%
Pharmacy Diploma	29	76%	31	86%	28	82%	88	81%
Pharmacy degree	3	8%	3	8%	0	0%	6	6%
Health-related master's degree	2	5%	0	0%	2	6%	4	4%
**Experience (Median [Range])**								
Total number of years worked	7	1-25	5	2-41	6	2-20	6	1-41
Number of years worked in the current pharmacy	3	1-14	3	1-30	3	1-17	3	1-30

[1] Staff designations included: pharmaceutical technologist (n=35), pharmacist (n=5), pharmacy assistant (n=2), and nurse aid (n=1)
[2] Non-health qualifications included: business management (n=1), supplies management (n=1), and college (n=1); health-related certificate/ diploma/ bachelor's degree included: medicine (n=1), nursing (n=2), and environmental health (n=1)h; health-related master's degree included: pharmacy (n=1), and other (n=3)
