# Supplementary material for: Experiences of and response to the COVID-19 pandemic at private retail pharmacies in Kenya: a mixed-methods study
Source: BMJ Open. 2022 Jun 28;12(6):e058688. doi: 10.1136/bmjopen-2021-058688 (PMC9240447; doi:10.1136/bmjopen-2021-058688)
Supplement: Supplementary data [file bmjopen-2021-058688supp003.pdf]

Supplementary Table II. Characteristics of in-depth interview participants. The interviewee # is used to reference illustrative quotes in the results section. Rows are sorted by interviewee #.

Interviewee #	County	Practice setting	Sex	Age category (years)	Highest level of education	Years in practice	Position
101	Nairobi	Urban residential area	Female	20 - 24	Pharmacy Diploma	4	Staff
102	Nairobi	Urban commercial center	Male	30 - 34	Pharmacy Diploma	10	Superintendent
103	Nairobi	Urban commercial center	Male	25 - 29	Pharmacy degree	2	Staff
104	Nairobi	Urban commercial center	Female	30 - 34	pharmacy assistant certificate	8	Staff
105	Nairobi	Urban commercial center	Male	25 - 29	Pharmacy Diploma	6	Superintendent
106	Nairobi	Urban residential area	Male	35 - 39	Pharmacy Diploma	10	Superintendent
201	Mombasa	Urban commercial center	Female	25 - 29	Pharmacy Diploma	5	In-charge
202	Mombasa	Urban commercial center	Male	40 - 44	Pharmacy Diploma	10	Owner or director
203	Mombasa	Urban informal settlement	Male	35 - 39	BSc supply chain	5	Owner or director
204	Mombasa	Urban commercial center	Male	25 - 29	Pharmacy Diploma	4	Staff
205	Mombasa	Urban commercial center	Male	25 - 29	Pharmacy Diploma	4	Superintendent
206	Mombasa	Urban commercial center	Female	20 - 24	Pharmacy Diploma	2	Staff
301	Kisumu	Urban commercial center	Male	25 - 29	Pharmacy Diploma	2	Staff
302	Kisumu	Rural town/ shopping center	Female	40 - 44	Nursing certificate	17	Staff
303	Kisumu	Urban commercial center	Male	30 - 34	Pharmacy Diploma	7	Superintendent
304	Kisumu	Rural town/ shopping center	Female	35 - 39	Pharmacy Diploma	7	Superintendent
305	Kisumu	Rural town/ shopping center	Female	30 - 34	Health-related masters	9	Owner or director
306	Kisumu	Rural town/ shopping center	Male	35 - 39	Health-related masters	10	Owner or director
