# Supplementary material for: Experiences of and response to the COVID-19 pandemic at private retail pharmacies in Kenya: a mixed-methods study
Source: BMJ Open. 2022 Jun 28;12(6):e058688. doi: 10.1136/bmjopen-2021-058688 (PMC9240447; doi:10.1136/bmjopen-2021-058688)
Supplement: Author's manuscript [file bmjopen-2021-058688.draft_revisions.pdf]

BMJ Open is committed to open peer review. As part of this commitment we make the peer review history of every article we publish publicly available.

When an article is published we post the peer reviewers' comments and the authors' responses online. We also post the versions of the paper that were used during peer review. These are the versions that the peer review comments apply to.

The versions of the paper that follow are the versions that were submitted during the peer review process. They are not the versions of record or the final published versions. They should not be cited or distributed as the published version of this manuscript.

BMJ Open is an open access journal and the full, final, typeset and author-corrected version of record of the manuscript is available on our site with no access controls, subscription charges or pay-per-view fees (http://bmjopen.bmj.com).

If you have any questions on BMJ Open's open peer review process please email info.bmjopen@bmj.com

BMJ Open

Experiences and response to the coronavirus disease 2019 (COVID-19) pandemic at private retail pharmacies in Kenya: a mixed methods study

Journal:	*BMJ Open*
Manuscript ID	bmjopen-2021-058688
Article Type:	Original research
Date Submitted by the Author:	26-Oct-2021
Complete List of Authors:	Mugo, Peter M.; KEMRI-Wellcome Trust Research Programme Nairobi, Health Economics Research Unit; KEMRI-Wellcome Trust Research Programme, Health Systems and Research Ethics Department<br>Mumbi, Audrey; KEMRI-Wellcome Trust Research Programme Nairobi, Health Economics Research Unit<br>Munene, Daniella; Pharmaceutical society of Kenya, CEO Office<br>Nzinga, Jacinta; KEMRI-Wellcome Trust Research Programme Nairobi, Health Economics Research Unit; KEMRI-Wellcome Trust Research Programme, Health Systems and Research Ethics<br>Molyneux, Sassy ; KEMRI-Wellcome Trust Research Programme, Health Systems and Research Ethics; University of Oxford, Nuffield Department of Medicine<br>Barasa, Edwine; KEMRI-Wellcome Trust Research Programme Nairobi, Health Economics Research Unit; University of Oxford, Nuffield Department of Medicine<br>Taskforce, COVID19 ; Pharmaceutical Society of Kenya (PSK), COVID-19 Response Taskforce
Keywords:	COVID-19, Public health < INFECTIOUS DISEASES, PRIMARY CARE, PUBLIC HEALTH, QUALITATIVE RESEARCH

SCHOLARONE™
Manuscripts

I, the Submitting Author has the right to grant and does grant on behalf of all authors of the Work (as defined in the below author licence), an exclusive licence and/or a non-exclusive licence for contributions from authors who are: i) UK Crown employees; ii) where BMJ has agreed a CC-BY licence shall apply, and/or iii) in accordance with the terms applicable for US Federal Government officers or employees acting as part of their official duties; on a worldwide, perpetual, irrevocable, royalty-free basis to BMJ Publishing Group Ltd ("BMJ") its licensees and where the relevant Journal is co-owned by BMJ to the co-owners of the Journal, to publish the Work in this journal and any other BMJ products and to exploit all rights, as set out in our licence.

The Submitting Author accepts and understands that any supply made under these terms is made by BMJ to the Submitting Author unless you are acting as an employee on behalf of your employer or a postgraduate student of an affiliated institution which is paying any applicable article publishing charge ("APC") for Open Access articles. Where the Submitting Author wishes to make the Work available on an Open Access basis (and intends to pay the relevant APC), the terms of reuse of such Open Access shall be governed by a Creative Commons licence – details of these licences and which Creative Commons licence will apply to this Work are set out in our licence referred to above.

Other than as permitted in any relevant BMJ Author's Self Archiving Policies, I confirm this Work has not been accepted for publication elsewhere, is not being considered for publication elsewhere and does not duplicate material already published. I confirm all authors consent to publication of this Work and authorise the granting of this licence.

For peer review only

Experiences and response to the coronavirus disease 2019 (COVID-19) pandemic at private retail pharmacies in Kenya: a mixed methods study

Short Title: Pharmacy COVID-19 Response

Peter Mugo[1,3]*, Audrey Mumbi[1], Daniella Munene[2], Jacinta Nzinga[1,3], Sassy Molyneux[3,4], Edwine Barasa [1,3,4], on behalf of PSK CRT[5]

[1] Health Economics Research Unit, Kemri-Wellcome Trust Research Programme, Nairobi, Kenya
[2] Pharmaceutical society of Kenya
[3] Health Systems and Research Ethics Department, KEMRI-Wellcome Trust Research Programme, Kilifi, Kenya
[4] University of Oxford, Headington, UK
[5] Pharmaceutical Society of Kenya (PSK) COVID-19 Response Taskforce (CRT)

*Corresponding author:
Peter Mwangi Mugo
KEMRI-Wellcome Trust Research Programme
P.O. Box 43640 – 00100 Nairobi, Kenya
Tel: + 254 730 162 000
E-mail: PMugo@kemri-wellcome.org

Key words: Private retail pharmacies, COVID-19 response, Kenya

For peer review only

Abstract

Objectives: To assess experiences and response to the COVID-19 pandemic at private retail pharmacies (community pharmacies). The main research question was: to what extent were pharmacies contributing to the national response and how can their contribution be maximized?

Setting: Pharmacies in 3 counties of Kenya (Nairobi, Mombasa, and Kisumu), selected purposively to represent the main urban centres.

Participants: This mixed-methods study included a questionnaire survey at 195 randomly selected pharmacies, a simulated client survey at 103 pharmacies, and in-depth interviews with 18 purposively selected survey respondents.

Results: The initial weeks of the pandemic were characterized by fear and panic among service providers and a surge in client flow. Subsequently, 61% of pharmacies experienced a dip in demand to below pre-pandemic levels and 31% reported challenges with unavailability, high price, and poor-quality of products. Almost all pharmacies were actively providing preventive materials and therapies; educating clients on prevention measures; counselling anxious clients; and handling and referring suspect cases. Fifty-nine pharmacies (55% [95% CI 45-65%]) reported ever receiving a client asking for COVID-19 testing and a similar proportion supported pharmacy-based testing. For treatment, most pharmacies (71%) recommended alternative therapies and nutritional supplements such as vitamin C; only 27% recommended conventional therapies such as antibiotics. While 48% had at least one staff member trained on COVID-19, a general feeling of disconnection from the national program prevailed.

Conclusions: Private pharmacies in Kenya were actively contributing to the COVID-19 response, but more deliberate engagement, support and linkages are required. Notably, there is an urgent need to develop guidelines for pharmacy-based COVID-19 testing, a service that is clearly needed and which could greatly increase test coverage. Roll-out of this and other pharmacy-based COVID-19 programs should be accompanied with implementation research in order to inform current and future pandemic responses.

Strengths and limitations

- The study used multiple quantitative and qualitative methods to enhance the comprehensiveness and reliability of the findings. Of note, we used simulated clients to objectively assess provider practices, an approach that is gaining acceptance as a gold standard for the measurement of clinical practice quality around the world.
- The study took advantage of information collected in a recent study to speed up recruitment and data collection.
- One weakness of the study was the low response rate (55%), considering that it targeted pharmacies that had participated in a similar study less than a year previously.

For peer review only

Introduction

The coronavirus disease 2019 (COVID-19) pandemic is an ongoing global health emergency [1, 2]. As of September 2021, over 230 million cases had been confirmed globally resulting in over 4.7 million deaths [3]. For the better part of 2020, non-pharmaceutical interventions such as early case detection, contact reduction, and hygiene measures were the main prevention strategies [4, 5]. In early 2021, following unprecedented public-private collaboration and speed in research and development, a number of highly effective vaccines became available [6, 7].

Non-pharmaceutical supportive care remains the key approach to treatment and management [8-10]. Among hospitalized patients, pharmaceuticals that have been shown to be effective include corticosteroids such as dexamethasone, antivirals such as remdesivir, and immunomodulators such as tocilizumab [8, 11, 12]. In the outpatient setting, pharmaceuticals used to manage mild COVID-19 cases include those targeting fever and pain such as ibuprofen, and those aiming to relieve respiratory congestion [9][12]. Other more specific agents that have been tried, such as hydroxychloroquine, ivermectin, and azithromycin, are discouraged [9, 12]. The World Health Organization and the US National Institutes of Health recommend that such unproven therapies be used only within the context of clinical trials [13, 14].

It is predicted that follow-up waves of COVID-19 infection may continue indefinitely [15]. Strategies are therefore required to enhance and sustain coverage, cost-effectiveness, and efficiency of prevention and treatment interventions. Private retail pharmacies (also referred to as community pharmacies) are a unique channel through which COVID-19 prevention and treatment interventions can be delivered, since they are often the first or only point of contact with the healthcare system in developing countries [16-18]. The main reasons for preferential care-seeking at pharmacies, compared to health facilities, include greater accessibility, lower cost, and greater perceived privacy [19, 20]. The International Pharmaceutical Federation highlights key areas where the pharmacy sector can support the pandemic response, including: management of the medication supply chain, patient education, identification and referral of patients to other health care providers, and provision of prevention materials and therapies [21, 22]. During the COVID-19 pandemic, pharmacies in many settings, including Kenya, were categorized as essential services hence they were exempted from closure [23].

The first COVID-19 case in Kenya was reported on 13th March 2020, and as of 12th October 2021, there were 251,248 confirmed cases and 5,190 deaths [24]. A number of control measures have been instituted by the government at different times depending on rates of infection and mortality [23, 25, 26]. Vaccine roll-out in Kenya started in March 2021 [27]. We assessed the preparedness and response to the COVID-19 pandemic at private retail pharmacies in Kenya, aiming to identify strategies for maximising their contribution to the national response.

For peer review only - http://bmjopen.bmj.com/site/about/guidelines.xhtml

Methods

Study design

We conducted a cross-sectional descriptive study, including a questionnaire survey, a simulated client survey, and in-depth interviews. A mixed-methods approach was used in data collection and analysis: insights from interviews were used to refine the survey tools and probe quantitative data; and survey data were used to refine the interview guide and qualitative data analysis. Given the physical distancing requirements at the time, data collection was done remotely.

Study settings and populations

The study was conducted in three counties of Kenya (Nairobi, Mombasa and Kisumu), selected purposively to represent the main urban centres. As of June 2020, the three counties together contributed 32% (1,602/5,033) of licensed pharmacies [28] and 78% (2,229/2,862) of reported COVID-19 cases [29] [24].

We targeted pharmacies that had participated in a pharmacy HIV prevention (PHP) study about a year previously (manuscript in preparation). Pharmacies had been selected randomly from the list of licensed pharmacies. Respondents were pharmacists (degree holders) and pharmaceutical technologists (diploma holders). Interview participants were purposively selected from among questionnaire respondents, targeting those that showed enthusiasm for the study through prompt and detailed responses. We aimed to increase variability of interview data by stratifying the sample by county, practice setting (urban vs. rural) and gender (male vs female).

Data collection

Data collection started in November 2020 (approximately seven months after the pandemic reached Kenya) and was concluded in December 2020 (for the surveys) and April 2021 (for the interviews).

One structured online questionnaire, taking about 20-30 minutes, was completed by each participating pharmacy (**Supplementary file A**). Data on pharmacy characteristics were imported from the PHP study database.

Simulated clients (SCs) made unscheduled phone calls to participating pharmacies, mimicking a client seeking COVID-19 preventive therapies (**Supplementary file B**). The SCs (2 women and 2 men, aged 25-35 years) had previously been engaged in the PHP study and trained comprehensively over a 2-week period. For the current study, further training was provided over a 1-week period through the Teams® digital meeting platform. An online debrief questionnaire was used to record observations (**Supplementary file C**). All calls were made on weekdays (Monday – Friday) between 8am and 5pm, over a 3-week period. Median [range] duration of calls was 4 [1-12] minutes. The majority (68%) of calls were made to a publicly available phone number, while the rest used a number in the study database as there was no public number. A few weeks after the simulated call, participating pharmacies were contacted to check if they

For peer review only - http://bmjopen.bmj.com/site/about/guidelines.xhtml

suspected any clients visiting the pharmacies during the survey period to be "fake" (detection survey).

In-depth interviews were conducted by the first and second authors using a semi-structured interview guide (**Supplementary file D**). Interviews were conducted via the Teams® digital meeting platform as a first option or a simple phone call if the participant so preferred or if internet connectivity was poor.

Data management and analysis

Quantitative data were captured directly into an open-source database (REDcap®, Vanderbilt University). Data cleaning and analysis was carried out using Stata® version 15 (College Station, Texas, USA). Summary statistics were compared across counties and multivariable modelling done using logistic regression. Systematicity in detection was assessed by correlating detection status with caller, pharmacy characteristics, and simulation process features.

Qualitative interviews were audio-recorded and transcribed verbatim in Microsoft Word®, translating into English where necessary. To ensure quality, all transcripts were cross-checked against the audio recordings. Transcripts were imported into NVivo 12 (SR International, Australia) and coded by the first and second authors. Analysis followed a thematic approach with initial categories based on the interview guide and emergent themes integrated in a second round of coding. A checklist on Consolidated criteria for Reporting Qualitative research (COREQ) was completed (**Supplementary file E**).

Ethical considerations

The study was approved by the Kenya Medical Research Institute (KEMRI) Scientific and Ethics Review Unit (KEMRI/ SERU/CGMR-C/208/4080). Participants signed written informed consent forms administered digitally.

Results

Response rate and characteristics of participants

Of 195 target pharmacies, 108 (55%) completed a questionnaire. Participating pharmacies were generally similar to non-participating pharmacies, but had features indicative of higher operational capacity and interest in public health interventions, such as having a computerized stock management system, written job descriptions, and providing blood pressure measurement (data not shown). Ninety-four (87%) pharmacies were located in urban areas; 44 (41%) had a consultation room; 71 (67%) had a computerised stock management system; and 13 (12%) were part of a chain network **(Supplementary table I)**. The majority of questionnaire respondents were male, below 40 years, and had a pharmacy diploma.

One hundred and three (95%) pharmacies received a simulated client call. Thirteen (13%) calls were determined as probably detected. Detection was less likely if the call was made by one

For peer review only - http://bmjopen.bmj.com/site/about/guidelines.xhtml

particular caller (perfect prediction), gender of person answering the call was male (adjusted odds ratio, aOR, 0.1, p=0.01); the pharmacy had a publicly available phone number (aOR 0.01, p=0.01), was part of a chain network (perfect prediction), opened 24 hours daily (perfect prediction), or had an on-site laboratory (perfect prediction). Main call outcomes (preventive therapies recommended, options for delivery of recommended products, and referral options) did not vary by detection status (data not shown), therefore all data were included in analysis.

Of 18 interview participants, 11 (61%) were male, 16 (88%) were below 40 years, 11 (61%) were pharmacy owner or in-charge, and 12 (67%) were diploma holders. Number of participants was balanced by county (6 each), but was skewed towards urban setting (13 v 5) and male gender (11 v 7) (**Supplementary Table II)**.

Impact of the pandemic on service providers and pharmacy operations

Interview participants described fear and panic when they first heard about COVID-19, especially regarding the risk of exposure to infection:

> *"In the beginning, it sent everybody into a panic mode. Including my entire staff where everybody now thought this is a killer disease and being healthcare workers, they must be people who will be among the victims."*
>
> - Male pharmaceutical technologist, Nairobi, 105

Some providers were in denial, feeling that the pandemic would never reach *"where we are"*, and others reported that some clients believed the pandemic was "*just an avenue maybe for our politicians ready to make some money or something*".

The main concerns reported in the questionnaire were: exposure to infection at work (66% of respondents), "keeping my family safe" (22%), meeting financial needs (6%), and being caught up in the curfew (3%).

One hundred (93%) of 108 pharmacies reported pandemic-induced operational challenges (**Figure 1**). The main supply-side challenge was unavailability and high price of products and the main demand-side challenge was reduced client flow.

Figure 1. Challenges experienced by pharmacies during the COVID-19 pandemic in Kenya

Interview data corroborated survey findings. Interviewees described temporal changes in client flow, with an initial surge of clients buying vitamins and other immune boosters, followed a few weeks later by a dip in demand to below pre-pandemic levels. The initial surge was accompanied by an increase in self-medication and stockpiling of long-term medications, such as for diabetes. While some of these requests were deemed potentially inappropriate, providers were not always able to effectively intervene:

For peer review only - http://bmjopen.bmj.com/site/about/guidelines.xhtml

"They just keep [these drugs] in case. …Like there was one [client] who came with a running nose, and then he was telling me he wanted Zithromax [a brand of the antibiotic azithromycin]. 'Get Flu gone, get antihistamine, this one will help you' [I told him]. So sometimes as a primary healthcare provider, you try to advise them, but now they have made up their mind, so you just go ahead and let them go [somewhere else]. Because whether you refuse, you do what, they've made up their mind."

- Male pharmaceutical technologist, Kisumu, 303

Participants attributed the subsequent reduction in demand mainly to the dusk-to-dawn curfew, despite the fact that pharmacy providers were classified as essential workers and issued curfew passes:

"Of course, the curfew really beat us down, because now by the time [it's] seven o'clock, people are going home… and it's our prime time to serve clients as from 7 pm. So, we as well missed out on business."

- Male pharmacy owner, Mombasa, 203

A number of interviewees believed that the decrease in service utilization was due to fear among clients, of infection and quarantine:

"Then people shied off from coming to the pharmacy especially the ones with coughs and flus because they were just thinking that I could tell them to go to hospital for the corona tests. So most clients decided to stay at home, not to come to the pharmacy for consultation, or they just sent somebody to buy them the drugs"

- Female pharmaceutical technologist, Kisumu, 304

One interviewee saw the stockpiling by clients in positive light, observing that it enabled him to offload slow-moving products:

"People resorted to scavenging where they might find these products and so that way [one was] able to liquidate what was lying in your shops"

- Male pharmaceutical technologist, Nairobi, 105

A reduction in hygiene-related illnesses was also hailed as a positive public health effect of the COVID-19 preventive measures:

"Due to proper maintenance of hygiene, the short-short illnesses have reduced. Washing of hands and stuff.""

- Male pharmacist, Nairobi, 103

Adjustments to pharmacy operations

Pharmacies adjusted their operations to cope with these challenges and to accommodate the government-instituted prevention measures: 74% reduced opening hours (mean 3 hours [range 1-7]); 38% reduced staffing; and 30% changed their meeting routine (**Table 1**). Adjustments did not vary significantly across counties.

For peer review only

For peer review only - http://bmjopen.bmj.com/site/about/guidelines.xhtml

Table 1. Adjustments to pharmacy operations following the outbreak of COVID-19 in Kenya

Change	Nairobi (N=38)		Mombasa (N=36)		Kisumu (N=34)		Total (N=108)	
	n	%	n	%	n	%	n	%
**Opening hours**[1]								
No change	7	18%	8	22%	10	29%	25	23%
Reduced open hours per day	31	82%	25	69%	24	71%	80	74%
Reduced open days per week	1	3%	4	11%	0	0%	5	5%
Closed temporarily	0	0%	0	0%	1	3%	1	1%
**Staffing**								
No change	19	50%	25	71%	19	59%	63	60%
Reduced number of staff	18	47%	9	26%	13	41%	40	38%
Increased number of staff	0	0%	1	3%	0	0%	1	1%
Reduced pay	1	3%	0	0%	0	0%	1	1%
**Stock volume levels**								
No change	12	32%	13	36%	8	24%	33	31%
Reduced overall	8	21%	12	33%	12	35%	32	30%
Increased overall	14	37%	10	28%	14	41%	38	35%
Both reduced and increased for different items	4	11%	1	3%	0	0%	5	5%
**Meeting routine**								
No change	24	63%	30	83%	22	65%	76	70%
Resorted to virtual/ remote/ online meetings[2]	8	21%	3	8%	7	21%	18	17%
Reduced frequency of meetings	4	11%	1	3%	3	9%	8	7%
Stopped meetings altogether	2	5%	1	3%	0	0%	3	3%
Reduced duration/ number of people	0	0%	1	3%	1	3%	2	2%
Changed timing	0	0%	0	0%	1	3%	1	1%

[1] Three pharmacies in Mombasa and one in Nairobi reduced their open days per week by one, while one pharmacy in Mombasa reduced by three days. One pharmacy in Kisumu closed temporarily for 2 weeks

[2] Main virtual platforms used included Zoom, Google Meet, and Whatsapp

COVID-19-related products and services provided by pharmacies

Table 2 presents the COVID-19-related products and services that were being provided or requested at pharmacies. Equipment and materials in highest demand were medical masks, N95/ K95 respirators, face shields, hand sanitizer, and thermometers; while the medications in highest demand were vitamin C, multivitamins, and antibiotics. Only 24 (22%) pharmacies reported keeping records related to COVID-19 services.

Table 2. COVID-19-related products and services provided by private retail pharmacies in Kenya. Data are number (%) of pharmacies providing or receiving requests (main row) and number of clients in past week (subsidiary row).

For peer review only

Product/ service	Nairobi (N=38)		Mombasa (N=36)		Kisumu (N=34)		Total (N=108)	
	N or median	% or IQR	N or median	% or IQR	N or median	% or IQR	N or median	% or IQR
**Equipment and materials**								
Cloth masks	1	3%	5	14%	6	18%	12	11%
Number of clients in past week	20	20-20	20	4-30	10	5-15	15	5-25
Medical Masks	37	97%	33	92%	30	88%	100	93%
Number of clients in past week	50	20-100	23	10-80	26	20-50	30	20-70
N95/ K95 masks	24	63%*	18	50%	11	32%	53	49%
Number of clients in past week	5	4-16	5	2-30	1	0-2	5	2-10
Goggles	6	16%	2	6%	3	9%	11	10%
Number of clients in past week	0	0-10	6	2-10	1	0-5	1	0-10
Face shields	19	50%*	10	28%	8	24%	37	34%
Number of clients in past week	1	0-6	1	0-3	1	0-8	1	0-5
Gowns/ coveralls	1	3%	1	3%	3	9%	5	5%
Number of clients in past week	0	0-0	10*	0-10	0	0-1	0	0-1
Hand sanitizer	37	97%	35	97%	31	91%	103	95%
Number of clients in past week	10	3-30	5	2-13	5	2-13	5	3-15
Thermometers	27	71%	23	64%	20	59%	70	65%
Number of clients in past week	2	1-5	2	1-5	1	0-2	2	2-3
Pulse oximeters	10	26%	11	31%	3	9%	24	22%
Number of clients in past week	1	1-3	2	0-10	1	0-2	2	1-3
**Medicines**								
Vitamin C[1]	35	92%*	29	81%	23	68%	87	81%
Number of clients in past week	10	10-30	15	10-30	5	3-11	10	4-20
Multivitamins[1]	29	76%	30	83%	24	71%	83	77%
Number of clients in past week	10	5-19	10	6-20	10	7-18	10	6-20
Traditional/ alternative/ natural remedies[1, 2]	1	3%	2	6%	0	0%	3	3%
Number of clients in past week	0	0-0	13	5-20	0	0-0	5	0-20
Hydroxychloroquine/ chloroquine	8	21%	7	19%	2	6%	17	16%
Number of clients in past week	3	2-7	1	0-5	3	0-5	2	0-5
Antibiotics[1, 3]	26	68%	22	61%	23	68%	71	66%
Number of clients in past week	3	2-10	15*	10-39	15	5-29	10	3-20
Steroids[1]	16	42%	16	44%	16	47%	48	44%
Number of clients in past week	3	1-8	9	5-19	10	4-15	7	3-15
Other medicines[4]	3	8%	4	11%	2	6%	9	8%
Number of clients in past week	3	1-5	28	23-45	3	1-4	13	3-28
**Information and counselling**								
How to protect themselves from infection	28	74%	28	78%	27	79%	83	77%
Number of clients in past week	5	2-13	5	3-15	10	3-20	5	3-15
Symptoms to look out for if they suspect infection	32	84%	28	78%	26	76%	86	80%
Number of clients in past week	3	2-10	5	4-10	9	2-11	5	2-11

For peer review only

Product/ service	Nairobi (N=38)		Mombasa (N=36)		Kisumu (N=34)		Total (N=108)	
	N or median	% or IQR	N or median	% or IQR	N or median	% or IQR	N or median	% or IQR
What to do if they develop symptoms/ suspect infection	22	58%	21	58%	16	47%	59	55%
Number of clients in past week	3	2-7	3	2-5	5	3-22	4	2-10
Mask use and related problems	19	50%	19	53%	20	59%	58	54%
Number of clients in past week	5	2-10	5	2-35	15	4-20	7	2-20
Counselling for anxiety related to getting infected/ becoming sick with COVID-19	14	37%	16	44%	13	38%	43	40%
Number of clients in past week	2	1-10	3	1-10	3	1-5	3	1-8
Counselling for anxiety about job/ business loss	5	13%	7	19%	5	15%	17	16%
Number of clients in past week	2	2-8	2	1-5	5	3-8	3	2-8
**Modes of communication with clients**								
Face-to-face at the pharmacy	33	87%	33	92%	34	100%	100	93%
Phone calls (including WhatsApp calls etc)	21	55%	25	69%	20	59%	66	61%
Texts (including WhatsApp messages, etc)	13	34%	15	42%	12	35%	40	37%
Facebook/ other web-based social media platforms	3	8%	2	6%	2	6%	7	6%

IQR: interquartile range
** Indicates significant difference between counties (p<0.05), based on chi square test or analysis of variance for means*
[1] A significant proportion of these sales may not have been related to COVD-19 prevention/ treatment
[2] Traditional/ alternative/ natural remedies included echinacea, chapa shoka, kaluma inhaler, menthol plus balm, and home remedies (lemon, papaya steaming)
[3] Antibiotics included azithromycin (n=55), cephalosporin (n=7), amoxicillin-containing (n=6), other macrolide (n=2); 3 pharmacies did not specify
[4] Other medicines included zinc+ vitamin D (n=4), vitamin B complex (n=1), salbutamol (n=1), and scotts emulsion (n=1); 2 pharmacies did not specify

Interview data suggested that many of these products and services were newly introduced following the outbreak, including products like vitamin C and surgical masks that one would have expected to find routinely in a pharmacy.

Demand for COVID-19 testing and provider views towards pharmacy-based testing

Fifty-nine (55% [95% CI 45-65%]) of 108 pharmacies reported ever receiving a client asking for COVID-19 testing, with a median [interquartile range] number of requests in the last week of 2 [1-4]. At the time of the survey, none of the pharmacies was providing COVID-19 testing; and among the 59 pharmacies experiencing demand for testing, alternatives given to clients included referral to other testing sites (94%) and body temperature measurement (15%). Referral options included: public health facilities (92%), private health facilities (27%), and private labs (13%).

Interviews revealed that test requests were mostly by clients with symptoms, those who suspected they had been exposed, and those who needed certification for travel or employment purposes (eg, drivers and hotel staff).

For peer review only - http://bmjopen.bmj.com/site/about/guidelines.xhtml

When asked if they thought COVID-19 testing should be provided in pharmacies, 60% [95% CI 50-70%] said "yes". Among the 64 that were supportive of pharmacy-based testing, main reasons for support were: pharmacies are easily accessible (36%), clients perceive pharmacies as more confidential (23%), clients shy away from health facilities (19%), and demand exists (9%).

In interviews, the confidentiality and convenience of the pharmacy setting was reiterated, with some interviewees suggesting home-based self-testing as an option:

> *"If it [testing] is available in pharmacies, somebody can just take the kit, you can just go home, you test yourself. If you find you are positive, you can just self-quarantine, without raising an alarm. So that when the quarantine period is over, you don't get that kind of stigma, rejection."*
>
> - Female pharmacy in charge, Mombasa, 201

Among the 42 that were not supportive of pharmacy-based testing, main reasons for opposing were: might be misused (29%), no private space in the pharmacy (24%), risk of infection (15%), protective gear is expensive or not available (12%), there is no demand for the service (10%), and staff are not adequately trained (10%). From the interviews, hesitancy to support pharmacy-based testing was mainly related to lack of capacity (*"I still need a thorough training…"*) and fear of infection.

Handling of simulated clients seeking covid-19-related services

When a simulated client called a pharmacy to ask if they have medicines that the client can use to protect themselves from getting COVID-19, 76 (74%) of 103 pharmacies recommended one or more medications: 27% recommended at least one conventional therapy and 71% at least one alternative therapy or nutritional supplement (**Table 3**). The main recommendations given to a client suspecting COVID-19 infection were to: get tested, visit the nearest health facility, and self-quarantine at home.

Table 3. Handling of simulated clients seeking COVID-19-related services at private retail pharmacies in Kenya.

Call outcome	Nairobi (N=38)		Mombasa (N=36)		Kisumu (N=34)		Total (N=103)	
	n	%	n	%	n	%	n	%
**Provider response to "*Do you have medicines that I can use to protect myself from getting COVID-19?*"**								
**Asked the client why he/she was asking for the medicines**	**14**	**38%***	**4**	**14%**	**2**	**6%**	**20**	**20%**
**Recommended conventional therapies**	**11**	**29%**	**6**	**19%**	**11**	**32%**	**28**	**27%**
Antibiotics	11	29%	6	19%	10	29%	27	26%
Azithromycin	9	24%	6	19%	9	26%	24	23%
Other antibiotics[1]	2	5%	1	3%	4	12%	7	7%
Painkillers	1	3%	0	0%	3	9%	4	4%

For peer review only - http://bmjopen.bmj.com/site/about/guidelines.xhtml

Call outcome	Nairobi (N=38)		Mombasa (N=36)		Kisumu (N=34)		Total (N=103)	
Antihistamines	0	0%	1	3%	2	6%	3	3%
**Recommended alternative therapies**	**29**	**76%**	**22**	**71%**	**22**	**65%**	**73**	**71%**
Vitamin C	28	74%	20	65%	19	56%	67	65%
Zinc	25	66%*	10	32%	7	21%	42	41%
Home remedies[2]	6	16%	4	13%	6	18%	16	16%
Vitamin B/ D	4	11%	4	13%	2	6%	10	10%
Multivitamins	2	5%	0	0%	3	9%	5	5%
Other remedies[3]	1	3%	0	0%	1	3%	2	2%
**Gave options on how to get the recommended medicines**	**37**	**97%**	**28**	**90%**	**34**	**100%**	**99**	**96%**
Pick from the pharmacy	21	55%	7	23%*	18	53%	46	45%
Home delivery	19	50%*	9	29%	4	12%	32	31%
Send someone to pick	5	13%	8	26%	4	12%	17	17%
**Provider response to "*What symptoms should I look out for if I suspect COVID-19 infection?*"**								
**Mentioned any symptoms**	**34**	**89%**	**29**	**94%**	**30**	**88%**	**93**	**90%**
Fever	23	61%	20	65%	21	62%	64	62%
Cough	13	34%	15	48%	16	47%	44	43%
Flu-like symptoms	15	39%	15	48%	12	35%	42	41%
Breathing difficulty	13	34%	12	39%	17	50%	42	41%
Loss/ change of smell or taste	16	42%	13	42%	11	32%	40	39%
Sore throat	15	39%*	8	26%	4	12%	27	26%
Chest pain or congestion	7	18%	4	13%	12	35%*	23	22%
Headache	6	16%	4	13%	8	24%	18	17%
Fatigue, tiredness, malaise	2	5%	8	26%*	4	12%	14	14%
Reduced appetite	2	5%	2	6%	7	21%	11	11%
Body aches/ pains	0	0%	3	10%	2	6%	5	5%
Gut symptoms, eg, stomach pain, diarrhea, etc	2	5%	0	0%	2	6%	4	4%
Dizziness	0	0%	2	6%	0	0%	2	2%
Weight loss	0	0%	0	0%	1	3%	1	1%
**Provider response to "*What should I do if I suspect I have COVID-19 infection?*"**								
**Immediate actions recommended**								
Get tested for COVID-19	25	66%	18	58%	16	47%	59	57%
Visit nearest health facility/ go see a doctor	18	47%	13	42%	24	71%*	55	53%
Self-quarantine/ isolate at home	3	8%	9	29%*	4	12%	16	16%
Take medications[4]	3	8%	2	6%	3	9%	8	8%
Check body temperature	2	5%	1	3%	2	6%	5	5%
Check oxygen levels	0	0%	1	3%	1	3%	2	2%
Check information online	0	0%	1	3%	1	3%	2	2%
Call 719[5]	2	5%	0	0%	0	0%	2	2%
**Supplementary actions recommended**								
Wear a mask	23	61%	16	52%	20	59%	59	57%
Maintain physical distance	21	55%	15	48%	18	53%	54	52%

For peer review only

Call outcome	Nairobi (N=38)		Mombasa (N=36)		Kisumu (N=34)		Total (N=103)	
Observe hand hygiene	7	18%	5	16%	7	21%	19	18%
Eat fruits	5	13%	5	16%	5	15%	15	15%
Eat well[6]	2	5%	3	10%	1	3%	6	6%
Take lots of fluids	1	3%	1	3%	3	9%	5	5%
Live well[7]	0	0%	0	0%	4	12%	4	4%
Other advice[8]	3	8%	1	3%	2	6%	6	6%
**Caller assessment of the interaction**								
**How friendly (approachable) was the person who served you?**								
1 - Very unfriendly	1	3%	0	0%	0	0%	1	1%
2 - Unfriendly	4	11%	3	10%	4	12%	11	11%
3 - Friendly	20	53%	22	71%	22	65%	64	62%
4 - Very friendly	13	34%	6	19%	8	24%	27	26%
**How enthusiastic was the provider about providing the service?**								
1 - Very unenthusiastic	1	3%	0	0%	0	0%	1	1%
2 - Unenthusiastic	4	11%	4	13%	7	21%	15	15%
3 - Enthusiastic	22	58%	22	71%	21	62%	65	63%
4 - Very enthusiastic	11	29%	5	16%	6	18%	22	21%
**Did the provider say or do anything that you found particularly helpful or nice?[9]**	**19**	**50%**	**9**	**29%**	**14**	**41%**	**42**	**41%**
**Did the provider say or do anything that you found particularly NOT helpful or NOT nice?[10]**	**4**	**11%**	**2**	**6%**	**7**	**21%**	**13**	**13%**

** Indicates significant difference between counties (p<0.05), based on chi square test*

[1] Other antibiotics included Augmentin, Amoxil, ceftriaxone, etc

[2] Home remedies included ginger/ lemon/ hot water drink, hot soup/ water, garlic, steam, frequent hot baths

[3] Other alternative therapies included ginsomin and omega-3

[4] Medications for treatment included: azithromycin (3%), other antibiotics (4%), painkillers (2%), steroids (1%), home remedies (1%)

[5] 719 is the government hotline for COVID-19 reporting and information-seeking

[6] Eat well: eat healthy, eat vegetables, etc

[7] Live well: exercise, enough rest, fresh air, don't panic, avoid stress, etc

[8] Other advice: don't touch face, avoid handshakes, do not take any medicines, don't reuse mask, etc

[9] Things that were deemed particularly helpful or nice related mainly to: reassurance that infection risk is low if prevention measures are followed; high chance of recovery in case of infection; detailed information on what to do or where to seek further help; and follow-up calls in cases where the provider was busy at the time of the initial call.

[10] Things that were deemed not helpful or not nice related mainly to: calls being hanged up; inattentiveness or being in a hurry to finish the call; and dismissiveness (eg, "don't you watch news? The symptoms are public knowledge!" and "we don't have those medicines you are asking for, jaribu kwingine [try elsewhere])

Infection prevention during service provision

The main infection prevention measures during service provision were hand hygiene, face masks, disinfection of services and physical distancing (**Table 4**). Eighty-five per cent of

For peer review only

respondents rated the prevention measures instituted at their pharmacy as either adequate (72%) or very adequate (13%). Only one pharmacy reported that a staff member had ever tested positive for COVID-19, though no pharmacy reported regular testing of staff.

Table 4. COVID-19 infection prevention during service provision by private retail pharmacies in Kenya.

Infection prevention measures	Nairobi (N=38)		Mombasa (N=36)		Kisumu (N=34)		Total (N=108)	
	n	%	n	%	n	%	n	%
**Among staff**								
Physical distancing[1]	25	66%	26	72%	27	79%	78	72%
Temperature monitoring	13	34%	14	39%	13	38%	40	37%
Hand hygiene	35	92%	33	92%	32	94%	100	93%
Disinfection of surfaces	29	76%	30	83%	27	79%	86	80%
Face masks[2]	34	89%	30	83%	30	88%	94	87%
Goggles	0	0%	1	3%	1	3%	2	2%
Ingestion of immune boosters	1	3%	0	0%	0	0%	1	1%
**Among clients**								
Physical distancing[1]	33	87%	31	86%	30	88%	94	87%
Temperature monitoring	13	34%	9	25%	11	32%	33	31%
Hand hygiene[3]	36	95%	33	92%	30	88%	99	92%
Face masks	27	71%	32	89%	27	79%	86	80%
Non-touch payment methods[4]	14	37%	16	44%	10	29%	40	37%
Information materials and messages[5]	12	32%	13	36%	11	32%	36	33%

[1] Physical distancing was achieved through various means including: reducing number of staff and working in shifts, ensuring a distance of 1.5 -3 meters between staff and clients through the use of posters and floor/seat markings, serving one client at a time or other crowd control measures, ribbons/ ropes/ glass/ metal-grill/ wooden barriers between client and provider, and home delivery of products. One pharmacy noted that "patients with cough and related symptoms are always first referred to the health center for covid19 testing before being served further".
[2] Majority of pharmacies (95%) were using surgical/medical masks; only 6% reported use of cloth masks.
[3] Hand hygiene measures among clients included: handwashing, hand sanitizer, and "no hand-shakes" policy
[4] Non-touch payment methods included: mobile money, and credit/ debit cards; one pharmacy reported disinfection of cash.
[4] Messages were delivered through: posters (21%), leaflets (5%), television screens (2%), and audio announcements (1%)

For peer review only

Information sources and training

Sources of general COVID-19 information included: continuing professional development sessions (37% of respondents), ministry of health (MoH) communications (23%), social media and internet (19%), television and radio (12%), Kenya Pharmaceutical Association (KPA)/ Pharmaceutical Society of Kenya (PSK) communications (5%), scientific journals (2%) and colleagues (2%). For specific questions about COVID-19, providers consulted the pharmacy-in-charge (48%), nearby doctor (37%), MoH officials (32%), the internet (5%), and medical representatives (2%).

Interviewees felt that the official announcement of the first case played was a major source of awareness, though some reported having learnt about the outbreak through radio, television,

For peer review only - http://bmjopen.bmj.com/site/about/guidelines.xhtml

and social media prior to the first reported case. However, they decried the rapidly changing and sometimes contradictory information, for example *"on whether to use or not to use masks".*

Fifty-two (48%) pharmacies had at least one staff member trained on COVID-19, with a median [range] of 1 [1-8] providers trained. This did not vary by county or by membership in professional associations. Modalities of training included: on-site in the pharmacy (67% of respondents), virtual (54%), and off-site (13%). Virtual trainings were more prevalent in Kisumu compared to Nairobi (76% vs 35%, p=0.04). Training sessions were facilitated by MoH (73%), KPA (51%), PSK (19%), AMREF (9%), and Kenyatta National Hospital (5%). While some interview participants felt they had received "a lot'' of training, others felt the trainings were not very adequate, particularly with regard to inclusion of updated information in follow-up sessions and the fact that most trainings included only the pharmacy in-charge and not the entire staff.

External support and linkages

Interviewees expressed appreciation for guidance from the ministry of health, including the pharmacy and poisons board, who "*did some inspection and brought some banners [information materials] on how I can protect myself and the general public."* The government-led community awareness-creation activities were also hailed as an important complement to pharmacy efforts in educating the public.

However, examples of direct linkages and material support were rare. One interviewee in Nairobi reported that community health workers from a nearby health centre regularly visited the pharmacy to collect information on number of suspect cases seen. Another interviewee in Kisumu described an instance where an ambulance was sent promptly on reporting a patient in critical condition.

More commonly, a feeling of isolation and neglect prevailed, with pharmacies reporting that they were not receiving much support from the government or other external parties with regard to COVID-19, unlike what happens with other public health programs:

> *"So far being a private facility, we are not receiving any financial support from the organizations in COVID related issues. We don't receive that apart from PS Kenya [NGO supporting TB and HIV programs]."*
>
> - Male pharmaceutical technologist, Kisumu, 301

Confidence, motivation, and coping mechanisms

Majority of questionnaire respondents reported feeling confident (68%) or very confident (19%) about providing COVID-19-related services. Over half felt prepared (51%) or very prepared (7%) about handling a client who presents to the pharmacy with COVID-19 symptoms.

Motivation for supporting delivery of COVID-19 services included: "to help people protect themselves or prevent transmission (75%), "It's part of my job/ I am required to do it" (14%),

For peer review only - http://bmjopen.bmj.com/site/about/guidelines.xhtml

encouragement and support from professional associations (7%), “to boost the profile of the pharmacy” (2%), and “to make money” (1%). When asked how much they felt their personal role in the COVID-19 response was valued by the community, the majority stated highly valued (68%) or very highly valued (11%)

In addition to the operational adjustments described above, interviewees described other coping mechanisms such putting up a shade outside to prevent crowding inside the pharmacy and improvisation of hand sanitizers when not available from suppliers. Some interviewees also reported going the extra mile sometimes, eg, by providing free masks to clients.

Provider suggestions and recommendations

Seventy-six (70%) questionnaire respondents highlighted inputs that they felt pharmacies can uniquely contribute to the national response, including: provision of information and counselling to clients (64%), distribution of preventive materials (21%), triage and referral of suspect cases (17%), surveillance of infection rates and community adherence to preventive measures (14%), provision of preventive and curative therapies (12%), and maintenance of long-term therapy (3%). Pharmacies’ location within communities was cited as a strength when it comes to surveillance of client behaviour and community awareness creation.

To facilitate these inputs, pharmacies suggested additional support that they would require from the government and other external parties, including: training (34% of respondents), free or subsidized preventive materials such as masks, sanitizers, and thermo guns for own use and distribution to clients (34%), supply management such as price and quality controls (15%), clear guidelines and referral systems (9%), community awareness creation (7%), information materials (6%), financial support such as loan repayment holidays and medical insurance (6%), and testing services (5%).

These suggestions were echoed in interviews. Post-marketing surveillance to ensure quality of products in the market and a streamlined process to capture and transmit information back to the government were pinpointed as key gaps in the pandemic response. Overall, there were earnest calls for more deliberate engagement, oversight, and support supervision by the government and other implementing partners:

> *“If the government could put us, the pharmacists and pharm techs in the you know, treat us like people who are fighting this virus and facilitate this thing it would be a major shift, it would be a major shift.”*
>
> - Male pharmacy in charge, Mombasa, 205

Discussion

In this study among private retail pharmacies (community pharmacies) in Kenya, we describe the impact of the COVID-19 pandemic on operations, COVID-19-related products and services being

For peer review only - http://bmjopen.bmj.com/site/about/guidelines.xhtml

provided, infection prevention during service provision, and provider suggestions and recommendations on how to improve pandemic preparedness and response.

The initial weeks after the pandemic reached Kenya were characterized by fear and panic among providers and a surge in client flow. Broadly speaking, the experiences documented in our study can be captured with the apt words of Austin et al in their paper describing the experiences of community pharmacists in Ontario, Canada: "the scale and magnitude of the COVID-19 pandemic defied imagination or pre-planning, yet health care professionals like pharmacists were required to continue to provide services and care to patients – and in many cases, expand their repertoire of clinical skills to assume new and even more challenging responsibilities" [15].

The initial surge in demand waned rapidly, with client flow dipping below pre-pandemic levels within a few weeks and remaining depressed until the time of data collection, seven months later. This was surprising as we had hypothesized, before the study, that pharmacies would experience an overall increase in demand as patients redirected their care-seeking from health facilities for fear of exposure to infection and quarantine. Participants attributed the decrease in demand mainly to the movement restrictions instituted by government. However, they also suggested – ironically – that clients, especially those with flu-like symptoms, may have avoided pharmacies for fear of being referred to public facilities and subsequently being quarantined.

Our findings clearly show that community pharmacies in Kenya were undertaking potentially impactful activities to support the response to the COVID-19 pandemic. Despite a feeling of disconnection from the government-led national response, providers expressed great readiness and motivation for provision of COVID-19-related services, similar to providers in Qatar [30], the USA [31], and other settings around the world [32]. The services pharmacies in our study were providing are generally similar to those suggested by experts in the field [22, 33-35], as well as those being provided in other low-to-middle income countries [36-39] and in high-income settings [30, 40, 41].

However, management of drug shortages and prevention of stockpiling (hoarding) by clients [22, 30, 33] were reported only as challenges and not as roles that Kenyan pharmacies were playing or could play. Additionally, while 60% of questionnaire respondents said they were receiving queries and providing information and counseling over the phone, other remote services such as online prescribing and home delivery of medications [22, 36] were not being widely used or mentioned as potential additional roles. Participants in our study, being frontline workers, may have deemed the carrying out of these innovations as being beyond the sphere of their influence. Similarly, COVID-19 vaccine administration was not mentioned as a potential role, understandably perhaps because a vaccine was not available at the time. But pharmacies in Kenya are actively involved in provision of other vaccines [42, 43], hence it is reasonable to hypothesize that COVID-19 vaccines could be delivered through private pharmacies in the future.

For peer review only - http://bmjopen.bmj.com/site/about/guidelines.xhtml

Interviews with policymakers and captains of industry may be required to probe these topics further.

The finding that no pharmacy was providing COVID-19 testing was unsurprising since no approved point-of-care (POC) tests were available at the time of the study. The more interesting finding is that there was significant demand and support for pharmacy-based testing, similar to what studies in the Middle East [44] and the USA [31] have found. In this context and given the fact that at least three rapid POC tests have since been approved in Kenya [45], there is an urgent need to start developing policies to guide implementation. This will obviate the emergence of an uncoordinated and potentially dangerous service, similar to the HIV testing one that we observed in 2016 before policy guidelines were issued [46, 47].

For treatment of COVID-19 symptoms, most pharmacies recommended alternative therapies and nutritional supplements such as vitamin C and zinc, and only about a third suggested conventional therapies with no proven efficacy such as antibiotics [13, 14]. This finding is similar to that of a small study in Nairobi in May 2020 which found that despite increased requests for antimalarials and antibiotics, pharmacists recommended alternative therapies [48]. A similar finding was also obtained in a multinational survey of Asian pharmacists [35]. While alternative therapies and nutritional supplements are themselves not evidence-based (as no rigorous trials have proven their efficacy against SARS-CoV-2), they have a better safety profile and their useful effects are more biologically plausible since they have been used for ages against the flu virus, itself a coronavirus. These findings are therefore encouraging. However, among Egyptian pharmacies, the antibiotic azithromycin was given to about 40% of presumptive COVID-19 patients with mild to moderate symptoms, albeit based on physician recommendation [49]. Further research in different jurisdictions may be needed to describe and contrast the use of antibiotics and other unproven COVID-19 therapies, in order to inform the promotion of rational drug use and the prevention of antimicrobial resistance.

Our study identified other important gaps in community pharmacies' preparedness and response to the pandemic. Notably, pharmacies did not seem to have any well-defined operational linkages to other units of the healthcare system, including for example, the emergency operations center at the ministry of health. Besides, while only about half of pharmacies had at least one staff member trained on COVID-19, some of those trained felt they had received a lot of training. This suggests a "network effect" that our study was unable to decipher, where a certain group of providers seem to be well-positioned to receive many trainings, while others receive none.

Specific programs will be required to address these gaps and enhance pandemic preparedness. Templates for public-private cooperation in pandemic responses do exist from other settings. Ung *et al*, for example, report how in one administrative region on the south-east coast of China, pre-existing public-private partnerships between the local government and community

For peer review only - http://bmjopen.bmj.com/site/about/guidelines.xhtml

pharmacies ensured rapid provision and prevented hoarding of face masks through a government-led “Guaranteed Mask Supply for Macao Residents Scheme” [50]. A qualitative study in Canada, identified key features that predicted community pharmacists’ resilience during the early period of the pandemic, including: use of and comfort with technology; early adoption of corporate and professional guidance; emphasis on task-focus rather than multi-tasking; and provision of personal protective equipment [15]. Roll-out of pharmacy-based COVID-19 programs should be accompanied with implementation studies to better uncover aspects of capacity, preparedness, and resilience.

Our study had a number of strengths. First, we took advantage of information collected in a recent study to speed up recruitment and data collection. Second, we used multiple quantitative and qualitative methods to enhance the comprehensiveness and reliability of the findings. Of note, we used simulated clients to objectively assess provider practices, an approach that is gaining acceptance as a gold standard for the measurement of clinical practice quality around the world [51]. One weakness of our study was the low response rate (55%), considering that we targeted pharmacies that had participated in a similar study less than a year previously. Compared to non-participating pharmacies, participating pharmacies had features suggestive of higher operational capacity and interest in public health interventions. While this limits generalizability of our findings, it can also be looked at in positive light. We propose that pharmacies with these characteristics could be identified for inclusion in a “practice and learning network” that could provide a platform for testing implementation strategies for various public health interventions [52-54]. Scale up of such interventions would then target similar pharmacies and include strategies for enhancing operational capacity and interest in public health among the other pharmacies.

In conclusion, we found that private retail pharmacies in Kenya were actively contributing to the COVID-19 response despite an apparent disconnection from the national response program. More deliberate engagement, support and linkages are required. Notably, there is an urgent need to develop policy guidelines for pharmacy-based COVID-19 testing, a service that is clearly needed and which could greatly increase test coverage. Roll-out of this and other pharmacy-based COVID-19 programs should be accompanied with implementation research in order to inform current and future pandemic responses.

Acknowledgments

We acknowledge the contributions of all participating pharmacy service providers. We thank James Wafula for supporting database development and data management; and Langat Justus, Lucy Omumbo, Milkah Auka, and Rodgers Ayoma for supporting the simulated client survey.

For peer review only - http://bmjopen.bmj.com/site/about/guidelines.xhtml

Author Contributions

Conceived the study: PM EB. Developed protocol: PM DM SM EB. Performed the experiments: PM AM; Analysed the data: PM AM JN. Wrote the manuscript: PM AM DM JN SM EB. Approved the final manuscript: PM AM DM JN SM EB.

The authors declare not conflicts of interest.

Funding statement

This work was supported by Wellcome Trust [#211353/Z/18/Z]. For the purpose of open access, the author has applied a CC-BY public copyright licence to any author accepted manuscript version arising from this submission. The KWTRP at the Centre for Geographical Medicine Research-Nairobi is supported by core funding from the Wellcome Trust [#077092]. This report was published with the permission of Director KEMRI CGMRC.

Patient and Public Involvement

Findings will be shared with the pharmacy sector at the 2021 Pharmaceutical Society of Kenya Annual Conference and a policy brief will be prepared targeting stakeholders in the ministry of health, pharmacy sector and other implementing partners.

References

1. *Zhu N, Zhang D, Wang W, Li X, Yang B, Song J, et al.* A novel coronavirus from patients with pneumonia in China, 2019. *New England Journal of Medicine* 2020.
2. *Anand U, Cabreros C, Mal J, Ballesteros Jr F, Sillanpää M, Tripathi V, et al.* Novel coronavirus disease 2019 (COVID-19) pandemic: From transmission to control with an interdisciplinary vision. *Environmental research* 2021:111126.
3. Coronavirus cases and deaths. https://www.worldometers.info/coronavirus/ Updated, Date Accessed: 29/09.
4. *Lai S, Ruktanonchai NW, Zhou L, Prosper O, Luo W, Floyd JR, et al.* Effect of non-pharmaceutical interventions for containing the COVID-19 outbreak: an observational and modelling study. *medRxiv* 2020.
5. *Cowling BJ, Ali ST, Ng TW, Tsang TK, Li JC, Fong MW, et al.* Impact assessment of non-pharmaceutical interventions against coronavirus disease 2019 and influenza in Hong Kong: an observational study. *The Lancet Public Health* 2020.
6. *Fauci AS*. The story behind COVID-19 vaccines: American Association for the Advancement of Science, 2021.
7. *Kashte S, Gulbake A, El-Amin III SF, Gupta A*. COVID-19 vaccines: rapid development, implications, challenges and future prospects. *Human cell* 2021:1-23.
8. *US National Institutes of Health*. Care of Critically Ill Adult Patients With COVID-19. 2020. https://www.covid19treatmentguidelines.nih.gov/critical-care/
9. *US National Institutes of Health*. Outpatient Management of Acute COVID-19. 2021. https://www.covid19treatmentguidelines.nih.gov/outpatient-management/
10. *Jin Y, Yang H, Ji W, Wu W, Chen S, Zhang W, et al.* Virology, epidemiology, pathogenesis, and control of COVID-19. *Viruses* 2020;12(4):372.
11. *Siemieniuk RA, Bartoszko JJ, Ge L, Zeraatkar D, Izcovich A, Kum E, et al.* Drug treatments for covid-19: living systematic review and network meta-analysis. *BMJ* 2020;370:m2980.

For peer review only - http://bmjopen.bmj.com/site/about/guidelines.xhtml

12. *American Society of Hospital Pharmacists*. Assessment of Evidence for COVID-19-Related Treatments. 2021. https://www.ashp.org/-/media/assets/pharmacy-practice/resource-centers/Coronavirus/docs/ASHP-COVID-19-Evidence-Table.ashx?la=en&hash=FD5D4C4AEDAC4B725B85D3375A88216E526C39EC
13. *World Health Organization*. Clinical management of COVID-19. 2020.
14. *US National Institutes of Health*. Prevention and Prophylaxis of SARS-CoV-2 Infection. 2021. https://www.covid19treatmentguidelines.nih.gov/overview/prevention-of-sars-cov-2/
15. *Austin Z, Gregory P*. Resilience in the time of pandemic: the experience of community pharmacists during COVID-19. *Research in Social and Administrative Pharmacy* 2021;17(1):1867-75.
16. *Abuya TO, Mutemi W, Karisa B, Ochola SA, Fegan G, Marsh V*. Use of over-the-counter malaria medicines in children and adults in three districts in Kenya: implications for private medicine retailer interventions. *Malar J* 2007;6:57.
17. *Bigogo G, Audi A, Aura B, Aol G, Breiman RF, Feikin DR*. Health-seeking patterns among participants of population-based morbidity surveillance in rural western Kenya: implications for calculating disease rates. *Int J Infect Dis* 2010;14(11):e967-73.
18. *Tran BX, Vu GT, Le HT, Pham HQ, Phan HT, Latkin CA, et al.* Understanding health seeking behaviors to inform COVID-19 surveillance and detection in resource-scarce settings. *Journal of Global Health* 2020;10(2).
19. *Fonck K, Mwai C, Rakwar J, Kirui P, Ndinya-Achola JO, Temmerman M*. Healthcare-seeking behavior and sexual behavior of patients with sexually transmitted diseases in Nairobi, Kenya. *Sex Transm Dis* 2001;28(7):367-71.
20. *Onwujekwe O, Hanson K, Uzochukwu B*. Do poor people use poor quality providers? Evidence from the treatment of presumptive malaria in Nigeria. *Trop Med Int Health* 2011;16(9):1087-98.
21. *International Pharmaceutical Federation*. Guidelines for Pharmacists and the Pharmacy Workforce to manage COVID-19. 2020. https://www.fip.org/publications?publicationCategory=97&publicationYear=2020&publicationKeyword=
22. *Aruru M, Truong H-A, Clark S*. Pharmacy Emergency Preparedness and Response (PEPR): a proposed framework for expanding pharmacy professionals' roles and contributions to emergency preparedness and response during the COVID-19 pandemic and beyond. *Research in Social and Administrative Pharmacy* 2021;17(1):1967-77.
23. *Shaw, N*. Kenya: protocols for the containment of the coronavirus (COVID-19) announced. 2020. https://www.ensafrica.com/news/detail/2373/kenya-announces-protocols-for-the-containment
24. *Worldometer*. Kenya Coronavirus Cases. 2021. https://www.worldometers.info/coronavirus/country/kenya/
25. *Wangari EN, Gichuki P, Abuor AA, Wambui J, Okeyo SO, Oyatsi HT, et al.* Kenya's response to the COVID-19 pandemic: a balance between minimising morbidity and adverse economic impact. *AAS Open Research* 2021;4.
26. *Barasa E, Kazungu J, Orangi S, Kabia E, Ogero M, Kasera K*. Assessing the Indirect Health Effects of the COVID-19 Pandemic in Kenya. *CGD Work. Pap* 2021;570.
27. *Global Alliance for Vaccines and Immunization*. Kenya completes its first round of COVID-19 vaccinations. 2021. https://www.gavi.org/vaccineswork/kenya-completes-its-first-round-covid-19-vaccinations
28. *Pharmacy and Poisons Board*. Online Services Portal: License Status. 2019. https://practice.pharmacyboardkenya.org/LicenseStatus

For peer review only

29. *Statista*. Cumulative number of confirmed coronavirus (COVID-19) cases in Kenya by county. 2021. https://www.statista.com/statistics/1136519/cumulative-coronavirus-cases-in-kenya-by-county/
30. *ElGeed H, Owusu Y, Abdulrhim S, Awaisu A, Kattezhathu VS, Abdulrouf PV, et al.* Evidence of community pharmacists' response preparedness during COVID-19 public health crisis: A cross-sectional study. *The Journal of Infection in Developing Countries* 2021;15(01):40-50.
31. *Nguyen E, Owens CT, Daniels T, Boyle J, Robinson RF*. Pharmacists' Willingness to Provide Coronavirus Disease (COVID-19) Services and the Needs to Support COVID-19 Testing, Management, and Prevention. *Journal of community health* 2020:1-6.
32. *Jordan D, Guiu-Segura JM, Sousa-Pinto G, Wang L-N*. How COVID-19 has impacted the role of pharmacists around the world. *Farmacia Hospitalaria: Organo Oficial de Expresion Cientifica de la Sociedad Espanola de Farmacia Hospitalaria* 2021;45(2):89-95.
33. *Liu S, Luo P, Tang M, Hu Q, Polidoro JP, Sun S, et al.* Providing pharmacy services during the coronavirus pandemic. *International Journal of Clinical Pharmacy* 2020;42(2):299-304.
34. *Bukhari N, Rasheed H, Nayyer B, Babar Z-U-D*. Pharmacists at the frontline beating the COVID-19 pandemic: Springer, 2020.
35. *Godman B, Haque M, Islam S, Iqbal S, Urmi UL, Kamal ZM, et al.* Rapid assessment of price instability and paucity of medicines and protection for COVID-19 across Asia: findings and public health implications for the future. *Frontiers in public health* 2020;8.
36. *Bahlol M, Dewey RS*. Pandemic preparedness of community pharmacies for COVID-19. *Research in Social & Administrative Pharmacy* 2021;17(1):1888.
37. *Meghana A, Aparna Y, Chandra SM, Sanjeev S*. Emergency preparedness and response (EP&R) by pharmacy professionals in India: lessons from the COVID-19 pandemic and the way forward. *Research in Social and Administrative Pharmacy* 2021;17(1):2018-22.
38. *Kasahun GG, Kahsay GM, Asayehegn AT, Demoz GT, Desta DM, Gebretekle GB*. Pharmacy preparedness and response for the prevention and control of coronavirus disease (COVID-19) in Aksum, Ethiopia; a qualitative exploration. *BMC health services research* 2020;20(1):1-6.
39. *Hoti K, Jakupi A, Hetemi D, Raka D, Hughes J, Desselle S*. Provision of community pharmacy services during COVID-19 pandemic: a cross sectional study of community pharmacists' experiences with preventative measures and sources of information. *International journal of clinical pharmacy* 2020;42(4):1197-206.
40. *Zaidi STR, Hasan SS*. Personal protective practices and pharmacy services delivery by community pharmacists during COVID-19 pandemic: Results from a national survey. *Research in Social and Administrative Pharmacy* 2021;17(1):1832-37.
41. *Giua C, Paoletti G, Minerba L, Malipiero G, Melone G, Heffler E, et al.* Community pharmacist's professional adaptation amid Covid-19 emergency: a national survey on Italian pharmacists. *International journal of clinical pharmacy* 2021:1-8.
42. *Goodlife Pharmacy*. Get your vaccinations today. 2021. https://www.goodlife.co.ke/get-your-vaccinations-today/
43. *Portal Pharmacy*. Flu vaccine. 2021. https://portalpharmacy.ke/flu-vaccine
44. *Mukattash TL, Jarab AS, Abu-Farha RK, Nusair M, Mukattash IL, Obaidat RM, et al.* Willingness and readiness to test for COVID-19; A qualitative exploration of community pharmacists. *International journal of clinical practice* 2020;74(12):e13620.
45. *Pharmacy and Poisons Board, Kenya Ministry of Health*. View Medical Devices. 2021. https://products.pharmacyboardkenya.org/ppb_admin/pages/system_reports_public.php

For peer review only - http://bmjopen.bmj.com/site/about/guidelines.xhtml

46. *Mugo PM, Micheni M, Shangala J, Hussein MH, Graham SM, Rinke de Wit TF, et al.* Uptake and Acceptability of Oral HIV Self-Testing among Community Pharmacy Clients in Kenya: A Feasibility Study. *PLoS One* 2017;12(1):e0170868.
47. *National AIDS and STI Control Programme (NASCOP)*. An operational manual for the delivery of HIV Self-Testing services in Kenya. 2017.
48. *Opanga SA, Rizvi N, Wamaitha A, Sefah IA, Godman B*. Availability of medicines in community pharmacy to manage patients with COVID-19 in Kenya; pilot study and implications. *Scholars Academic Journal of Pharmacy* 2021;10(3):36-43.
49. *Elsayed AA, Darwish SF, Zewail MB, Mohammed M, Saeed H, Rabea H*. Antibiotic misuse and compliance with infection control measures during COVID-19 pandemic in community pharmacies in Egypt. *International journal of clinical practice* 2021;75(6):e14081.
50. *Ung COL*. Community pharmacist in public health emergencies: Quick to action against the coronavirus 2019-nCoV outbreak. *Research in Social and Administrative Pharmacy* 2020;16(4):583-86.
51. *Daniels B, Dolinger A, Bedoya G, Rogo K, Goicoechea A, Coarasa J, et al.* Use of standardised patients to assess quality of healthcare in Nairobi, Kenya: a pilot, cross-sectional study with international comparisons. *BMJ global health* 2017;2(2):e000333.
52. *English M, Ayieko P, Nyamai R, Were F, Githanga D, Irimu G*. What do we think we are doing? How might a clinical information network be promoting implementation of recommended paediatric care practices in Kenyan hospitals? *Health research policy and systems* 2017;15(1):1-12.
53. *Gachau S, Ayieko P, Gathara D, Mwaniki P, Ogero M, Akech S, et al.* Does audit and feedback improve the adoption of recommended practices? Evidence from a longitudinal observational study of an emerging clinical network in Kenya. *BMJ global health* 2017;2(4):e000468.
54. *Tuti T, Bitok M, Malla L, Paton C, Muinga N, Gathara D, et al.* Improving documentation of clinical care within a clinical information network: an essential initial step in efforts to understand and improve care in Kenyan hospitals. *BMJ Global Health* 2016;1(1):e000028.

For peer review only

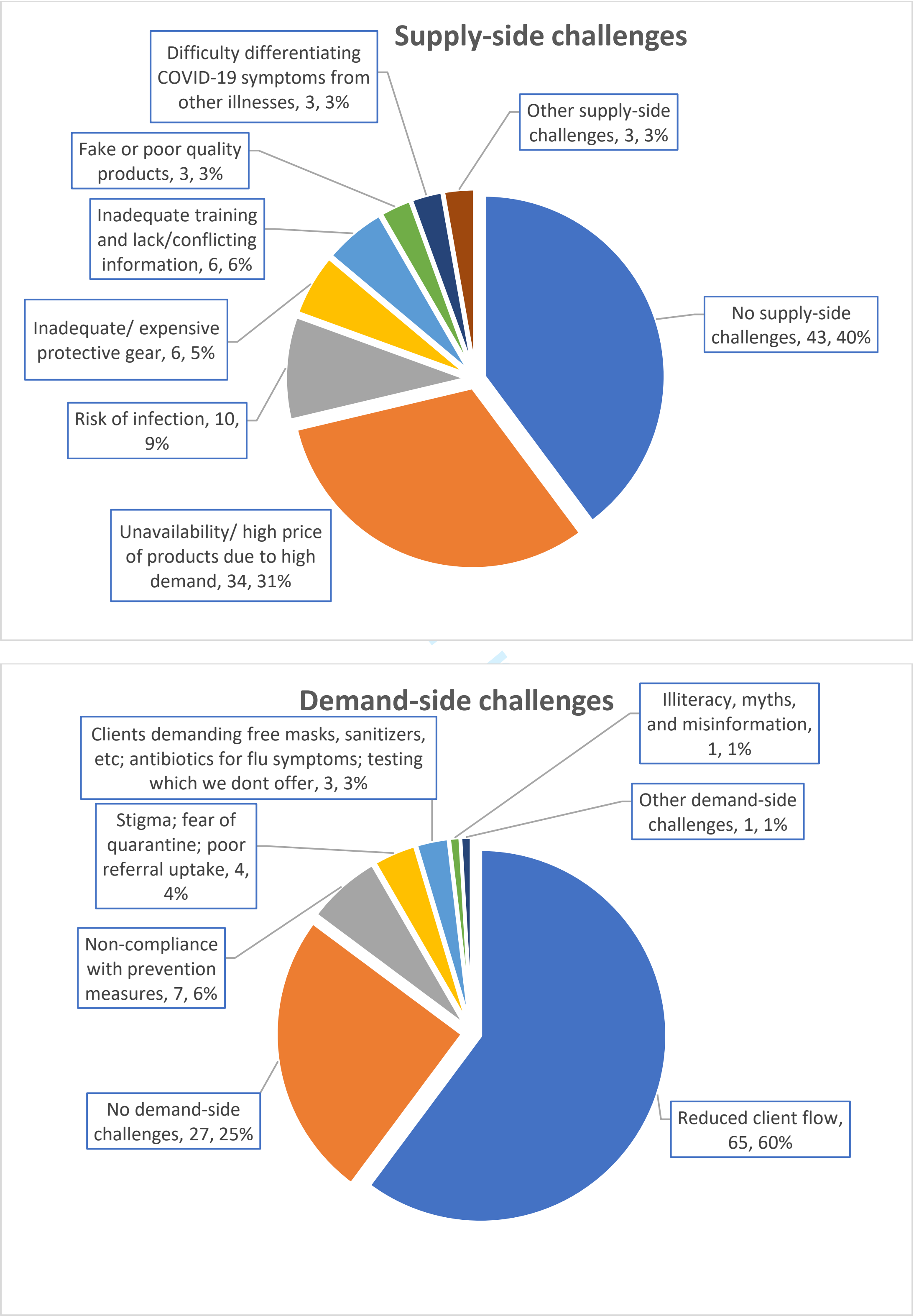

Figure 1. Challenges experienced by pharmacies during the COVID-19 pandemic in Kenya

Supplementary Table I: Characteristics of participating pharmacies and respondents

A. Characteristics of participating pharmacies

Characteristic	Nairobi (n=38)		Mombasa (n=36)		Kisumu (n=34)		Total (n=108) ^	
	n	%	n	%	n	%	n	%
**Setting**								
Urban commercial centre	23	61%	29	81%	12	35%	64	59%
Urban residential area	13	34%	4	11%	6	18%	23	21%
Urban informal settlement	2	5%	3	8%	2	6%	7	6%
Rural town/ shopping centre	0	0%	0	0%	14	41%*	14	13%
**Years of operation**								
Number of years in operation (median [range])	5	1-19	4	1-42	5	1-11	5	1-42
**Physical facilities**								
Total number of rooms (Median [range])	2	1-10	2	1-6	2	1-8	1	1-10
Consultation room	14	37%	10	28%	20	59%*	44	41%
On-site lab	5	13%	1	3%	6	18%	12	11%
**Information systems**								
Computerized stock system	29	81%*	19	53%	23	68%	71	67%
Individual medical records[1]	9	25%	2	6%	7	21%	18	17%
**Chain network membership[2]**								
Part of a chain network	9	24%*	3	8%	1	3%	13	12%
Number of branches nationwide (Median [range])	1	1-41	1	1-41	1	1-9	1	1-41
**Branding[3]**								
None	33	87%	29	81%	32	94%	94	87%
Green Cross	2	5%	2	6%	0	0%	4	4%
Pharmnet (blue cross)	0	0%	3	8%	1	3%	4	4%
Corporate	3	8%	2	6%	1	3%	6	6%
**Distance to the nearest clinic**								
Within the pharmacy	0	0%	0	0%	2	6%	2	2%
< 100 meters	17	45%	13	36%	9	26%	39	36%
< 1km	19	50%	16	44%	21	62%	56	52%
< 5km	2	5%	7	19%	2	6%	11	10%
**Distance to the nearest HIV testing site**								
< 100 meters	14	37%	12	33%	11	32%	37	34%
< 1km	18	47%	16	44%	21	62%	55	51%
< 5km	4	11%	7	19%	2	6%	13	12%
>5km	1	3%	0	0%	0	0%	1	1%
Not sure	1	3%	1	3%	0	0%	2	2%
**Service providers**								
Total number of service providers (Median [range])	3*	1-16	2	1-9	2	1-4	2	1-16
At least one pharmacy degree holder	8	21%	7	19%	3	9%	18	17%

For peer review only

Characteristic	Nairobi (n=38)		Mombasa (n=36)		Kisumu (n=34)		Total (n=108) ^	
	n	%	n	%	n	%	n	%
At least one pharmacy diploma holder	36	95%	35	97%	34	100%	105	97%
All staff belong in a professional association	24	63%	19	53%	18	53%	61	56%
**Management**								
Written job descriptions	21	55%	12	33%	14	41%	47	44%
Regular staff meetings[4]	21	55%	16	44%	19	56%	56	52%
**Opening hours**								
24 hours daily, 7 days a week	4	11%	1	3%	1	3%	6	6%
At least seven days a week	23	61%	28	78%	25	74%	76	70%
At least 6 days a week	38	100%	34	94%	34	100%	106	98%
**Work load**								
Walk-in clients per day (Median [range])	55	15-300	50	20-200	40	20-200	50	15-300
Online clients per day (Median [range])	1	0-20	0	0-20	0	0-10	0	0-20
**Health promotion services**								
Over-the-counter STI treatment	29	76%	29	81%	30	88%	88	81%
Weight management	12	32%	6	17%	10	29%	28	26%
Smoking cessation	4	11%	1	3%	1	3%	6	6%
Vaccination	0	0%	1	3%	3	9%	4	4%
**Screening services**								
Blood pressure measurement	26	68%	26	72%	25	74%	77	71%
Blood sugar testing	19	50%	25	69%	20	59%	64	59%
HIV self-testing	21	55%	22	61%	20	59%	63	58%
Malaria testing	9	24%	19	53%	27	79%*	55	51%
In-pharmacy pregnancy testing	9	24%	9	25%	18	53%*	36	33%
Other screening services[5]	1	3%	0	0%	5	15%*	6	6%

^ Of 195 target pharmacies that had participated in the previous PHP study, 108 (55%) participated in the current survey. Of 87 that did not participate: 55 (63%) failed to complete the questionnaire after three reminders, 15 (17%) reported that the initial respondent had left and no one else was willing to participate, 8 (9%) were unreachable, 3 (3%) reported that the management did not approve participation, 2 (1%) were undergoing transition, 1 (1%) was closed, and 3 (3%) had other reasons (no access to smartphone or computer, preferred face-to-face interview, sold the pharmacy).
** Indicates significant difference between counties (p<0.05), based on chi square test or analysis of variance for means*
[1] An individual medical record is a document detailing the medications an individual patient is on, indications and drug allergies; may also include other clinical information.
[2] Chain network was defined as 6 or more branches nationwide, derived from chain size distribution pattern; 79 (73%) pharmacies were single branch, 16 (15%) were part of a 2-5 branch network, and 13 (12%) were part of a chain network
[3] Green Cross is a professional network of pharmacies owned by pharmacists; Pharmnet is for pharmaceutical technologists
[4] Excludes 18 pharmacies that responded "not applicable" since they have only one staff member
[5] Other screening services included; typhoid testing (n=4) and tuberculosis screening (n=2)
STI: Sexually transmitted infection

For peer review only

B. Characteristics of respondents

Characteristic	Nairobi (N=38)		Mombasa (N=36)		Kisumu (N=34)		Total (N=108)	
	n	%	n	%	n	%	n	%
**Gender**								
Female	12	32%	13	36%	16	47%	41	38%
Male	26	68%	23	64%	18	53%	67	62%
**Age**								
20 – 29 years	12	32%	12	33%	14	41%	38	35%
30 – 39 years	19	50%	18	50%	16	47%	53	49%
40 – 44 years	5	13%	4	11%	2	6%	11	10%
45 years and over	2	5%	2	6%	2	6%	6	6%
**Designation (role)**								
Owner	9	24%	12	33%	12	35%	33	31%
In-charge/ Superintendent	11	29%	13	36%	8	24%	32	30%
Staff[1]	18	47%	11	31%	14	41%	43	40%
**Highest level of education[2]**								
Non-health qualifications	0	0%	2	6%	1	3%	3	3%
Health-related certificate/ diploma/ bachelor's degree	2	5%	0	0%	2	6%	4	4%
Pharmacy certificate	2	5%	0	0%	1	3%	3	3%
Pharmacy Diploma	29	76%	31	86%	28	82%	88	81%
Pharmacy degree	3	8%	3	8%	0	0%	6	6%
Health-related master's degree	2	5%	0	0%	2	6%	4	4%
**Experience (Median [Range])**								
Total number of years worked	7	1-25	5	2-41	6	2-20	6	1-41
Number of years worked in the current pharmacy	3	1-14	3	1-30	3	1-17	3	1-30

[1] Staff designations included: pharmaceutical technologist (n=35), pharmacist (n=5), pharmacy assistant (n=2), and nurse aid (n=1)
[2] Non-health qualifications included: business management (n=1), supplies management (n=1), and college (n=1); health-related certificate/ diploma/ bachelor's degree included: medicine (n=1), nursing (n=2), and environmental health (n=1)h; health-related master's degree included: pharmacy (n=1), and other (n=3)

For peer review only

Supplementary Table II. Characteristics of in-depth interview participants. The interviewee # is used to reference illustrative quotes in the results section. Rows are sorted by interviewee #.

Interviewee #	County	Practice setting	Sex	Age category (years)	Highest level of education	Years in practice	Position
101	Nairobi	Urban residential area	Female	20 - 24	Pharmacy Diploma	4	Staff
102	Nairobi	Urban commercial center	Male	30 - 34	Pharmacy Diploma	10	Superintendent
103	Nairobi	Urban commercial center	Male	25 - 29	Pharmacy degree	2	Staff
104	Nairobi	Urban commercial center	Female	30 - 34	pharmacy assistant certificate	8	Staff
105	Nairobi	Urban commercial center	Male	25 - 29	Pharmacy Diploma	6	Superintendent
106	Nairobi	Urban residential area	Male	35 - 39	Pharmacy Diploma	10	Superintendent
201	Mombasa	Urban commercial center	Female	25 - 29	Pharmacy Diploma	5	In-charge
202	Mombasa	Urban commercial center	Male	40 - 44	Pharmacy Diploma	10	Owner or director
203	Mombasa	Urban informal settlement	Male	35 - 39	BSc supply chain	5	Owner or director
204	Mombasa	Urban commercial center	Male	25 - 29	Pharmacy Diploma	4	Staff
205	Mombasa	Urban commercial center	Male	25 - 29	Pharmacy Diploma	4	Superintendent
206	Mombasa	Urban commercial center	Female	20 - 24	Pharmacy Diploma	2	Staff
301	Kisumu	Urban commercial center	Male	25 - 29	Pharmacy Diploma	2	Staff
302	Kisumu	Rural town/ shopping center	Female	40 - 44	Nursing certificate	17	Staff
303	Kisumu	Urban commercial center	Male	30 - 34	Pharmacy Diploma	7	Superintendent
304	Kisumu	Rural town/ shopping center	Female	35 - 39	Pharmacy Diploma	7	Superintendent
305	Kisumu	Rural town/ shopping center	Female	30 - 34	Health-related masters	9	Owner or director
306	Kisumu	Rural town/ shopping center	Male	35 - 39	Health-related masters	10	Owner or director

For peer review only

Supplementary file A – PHARMACY QUESTIONNAIRE

Note to interviewer: This questionnaire is targeted at the pharmacy service provider (PSP) previously included in the PHP study. In case they are not available, target the in-charge or most experienced or most highly-educated PSP, in that order.

BASIC INFORMATION
Interviewer identifier; Pharmacy identifier; Date; Time

INTRODUCTION
Thank you for finding time for this interview. I would like to ask you some questions regarding general operations in your pharmacy and your experience providing COVID19-related services…

SECTION I: RESPONDENT CHARACTERISTICS

1. What is your gender? Male/Female/other, specify
2. What is your age category in years?
   18 – 24
   25 – 29
   30 – 34
   35 – 39
   40 – 44
   45 – 49
   50 – 54
   55 – 59
   >59

3. What position do you hold in the pharmacy? [pharmacy owner or director/ Superintendent/ Pharmacy in-charge/ Pharm Tech/ Pharmacist/ other, specify]
4. What is your highest level of education? Pharmacy degree/ Pharmacy Diploma / Other
5. How many years have you worked in the retail/ community pharmacy sector? [numeric; if less than a year, enter 0.5]
   a. How many years have you worked in the current pharmacy?

SECTION II: OVERALL EFFECT OF THE COVID19 EPIDEMIC ON PHARMACY OPERATIONS

6. Have you had to make any changes to your operations since the outbreak of COVID19 epidemic in Kenya (mid-March 2020)? Yes/no
   a. Have you had to change your hours of operation? [can select more than one]
      No
      Reduced open hours per day, specify number of hours reduced
      Reduced open days per week, specify number of days reduced
      Closed for some time then re-opened, specify closure period in weeks
      Closed indefinitely, specify date of closure
      Other, specify
   b. Have you had to change number of staff? [select one]
      No
      Reduced
      Increased
      Other, specify
   c. Have you had to change stocking levels? [can select more than one]
      No
      Reduced stock volumes [specify 3 main items reduced in volume]
      Increased stock volumes [specify 3 main items increased in volume]

For peer review only - http://bmjopen.bmj.com/site/about/guidelines.xhtml

d. Have you had to change your meeting routine? Yes/ no
If yes, describe [open-ended]

7. Have you observed any changes in client flow due to the COVID19 outbreak? [select one]
   No
   Decreased client flow overall
   Increased client flow overall
   Both decrease and increase in different client categories

   a. If both decrease and increase,
      i. Which client category(ies) have decreased? [open-ended]
8. Which client category(ies) have increased? [open-ended]

SECTION III: PROVISION OF COVID19-RELATED SERVICES AND PRODUCTS

9. Which COVID-related equipment and materials does your pharmacy provide? [indicate number of clients in the last one week]
   None
   Cloth masks [number clients]
   Medical masks [number clients]
   N95/ K95 masks/ respirators [number clients]
   Goggles [number clients]
   Face shields [number clients]
   Gowns/ coveralls [number clients]
   Hand sanitizer [number clients]
   Thermometers [number clients]
   Pulse oximeters [number clients]
   Other, specify type and number of clients
   Specify other COVID19-related equipment and materials that your pharmacy provides

10. Which COVID-related preventive and curative therapies does your pharmacy provide? [indicate number of clients in the last one week]
   None
   Vitamin C [number clients]
   Multivitamins [number clients]
   Traditional/ alternative/ natural remedies, specify [number clients]
   Hydroxychloroquine/ chloroquine [number clients]
   Antibiotics, specify [number clients]
   Antivirals, specify [number clients]
   Immunomodulators, specify [number clients]
   Steroids, specify [number clients]
   Other, specify type [number of clients]
   Specify other preventive and curative therapies provided

   Indicate **number of clients** who bought or requested vitamin C in the last one week
   How many of these vitamin C purchases do you think were COVID-related? [numeric]

   [same format for related subsequent questions]

11. Which COVID-related information and counselling do clients seek from your pharmacy? [indicate number of clients in the last one week]
   None

For peer review only - http://bmjopen.bmj.com/site/about/guidelines.xhtml
For peer review only

How to protect themselves from infection [number clients]
Symptoms to look out for if they suspect infection [number clients]
What to do if they develop symptoms or suspect infection [number clients]
Mask use and related problems [number clients]
Counselling for anxiety related to getting infected/ becoming sick with COVID19 [number clients]
Counselling for anxiety about job or business loss [number clients]
Other, specify [number clients]

Specify other type of information or counselling that clients seek: ___________________________

Indicate number of clients in the last one week who asked how to protect themselves from infection

Indicate number of clients in the last one week who asked about symptoms to look out for if they suspect infection

Indicate number of clients in the last one week who asked what to do if they develop symptoms or suspect infection

Indicate number of clients in the last one week who asked about mask use and related problems

Indicate number of clients in the last one week who sought counselling for anxiety related to getting infected or becoming sick with COVID19

Indicate number of clients in the last one week who sought counselling for anxiety about job or business loss

Indicate number of clients in the last one week who sought for other type of information or counselling

12. Select the modes of communication that clients use to seek information/ counselling
    In-person (visit the pharmacy physically)
    Phone calls (including WhatsApp calls etc)
    Texts (including WhatsApp)
    Facebook/ other web-based platforms
    Other, specify

13. What records do you keep regarding COVID-related services and products? [open-ended]
14. Do you prepare any reports regarding COVID-related services and products? Yes/no
    a. To whom do you send the reports?
        Don't send (reports are for internal use)
        Ministry of health (MoH)/ government agency
        Other, specify
    b. What type of information is contained in the reports? [open-ended]

15. What challenges have you had in providing COVID-related services and products? [open-ended]
16. What would you say is your main motivation for supporting delivery of COVID-related services and products? [select one]
        Help people protect themselves/ Help prevent transmission
        Boost the profile of the pharmacy
        Make money
        It's part of my job/ I am required to do it
        Encouragement/ support from professional association (PSK/ KPA/ etc)
        Other, specify
17. How confident do you feel about providing COVID-related services and products? [select one]

For peer review only - http://bmjopen.bmj.com/site/about/guidelines.xhtml

Very uncertain/ uncertain/ confident/ very confident

18. What additional support do you feel pharmacies require in provision of COVID-related services and products? [open-ended]
19. If you don't provide COVID-related products or services,
    a. Do you get clients who ask for COVID-related services or products? Yes/no
        i. If yes, what help do you offer them? Referral/ other
            1. If you refer, to where? Another pharmacy/ clinic/ Hospital/ Lab/ other
    b. What COVID-related services or products do clients ask for?
        Personal protective equipment and materials
        Preventive and curative therapies
        Information/ counselling on COVID19
        Other, specify
    c. Select the type of information/ counselling that clients seek, and indicate number of clients in the last one week [select one]
        How to protect themselves from infection [number clients]
        Symptoms to look out for if they suspect infection [number clients]
        What to do if they develop symptoms/ suspect infection [number clients]
        Mask use and related problems
        Counselling for anxiety related to getting infected/ becoming sick with COVID19 [number clients]
        Counselling for anxiety about job/ business loss [number clients]
        Other, specify [number clients]
    d. Select the modes of communication that clients use to seek information, and indicate number of clients in the last one week [select one]
        In-person (visit the pharmacy physically)
        Phone calls (including WhatsApp calls etc)
        Texts (including WhatsApp)
        Facebook/ other web-based platforms
        Other, specify
20. Has any staff member been trained on COVID19?
    a. How many current staff members have been trained on COVID19?
    b. Where did the training take place? On-site/ off-site/ virtual
    c. Who provided the training? MoH/ PSK/ KPA/ Other, specify
21. When you have questions about COVID19, who do you consult? [can select more than one]
    None/ don't consult
    MoH
    Pharmacy in-charge/ manager/ colleague (s)
    Med reps
    Nearby doctor
    Other, specify
22. What is your main source of COVID19 information? [select one]
    TV/ Radio
    Webinar
    Social media
    Google search/ internet
    Scientific journals
    Colleagues/ other pharmacy
    KPA/ PSK communications
    CME/ CPD session
    MoH communications
    Other, specify

<u>SECTION IV: INFECTION PREVENTION</u>

For peer review only

For peer review only - http://bmjopen.bmj.com/site/about/guidelines.xhtml

23. Which infection prevention measure(s) is your pharmacy using among staff? [can select more than one]
    - None
    - Physical distancing, describe
    - Temperature monitoring
    - Hand hygiene (handwashing, hand sanitizer, etc)
    - Disinfection of surfaces
    - Face masks
    - Goggles
    - Other, specify
24. Which infection control measure(s) is your pharmacy using among clients? [can select more than one]
    - None
    - Physical distancing, describe
    - Temperature monitoring before entering the pharmacy
    - Require clients to be wearing face masks before entering the pharmacy
    - Hand washing
    - Hand sanitizer
    - Use of non-touch payment methods, describe
    - Information materials and messages
    - Other, specify

    Which information materials and messages is your pharmacy providing to clients?
      - Posters/ banners
      - Leaflets/ pamphlets
      - Audio announcements
      - TV screen displays
      - Other, specify

25. Which method do staff members mainly use for cleaning their hands? [select one]
    - Handwashing
    - Hand sanitizer
    - Other, specify

    a. [If handwashing is main] In the last 5 working days,
        i. On how many days was water available for handwashing in your pharmacy? [enter value between 0 and 5]
        ii. On how many days was soap available for handwashing in your pharmacy? [enter value between 0 and 5]
    b. [If hand sanitizer is main] In the last 5 working days,
        i. On how many days was hand sanitizer available in your pharmacy? [enter value between 0 and 5]
26. What type of masks do staff use in your pharmacy? [can select more than one]
    - Cloth masks
    - Surgical/ medical masks
    - N95 or similar
    - Other, specify
27. Have staff members been trained on COVID19 infection prevention? Yes/no
    a. If yes, by who? [can select more than one]
        - MoH
        - Pharmacy in charge/ colleague
        - Other, specify
    b. Where did the training take place? [can select more than one]
        - Off-site, specify venue
        - In-person within the pharmacy

For peer review only - http://bmjopen.bmj.com/site/about/guidelines.xhtml

Remotely (virtually) when at the pharmacy
Remotely (virtually) when at home
Other, specify

28. Do you have any written guidelines on COVID19 infection prevention? Yes/no
    a. If yes, describe the guidelines you have access to (e.g. source, title, etc) [open-ended]
29. How adequate do you feel the infection prevention measures in your pharmacy are? [select one]
    Very inadequate/ inadequate/ adequate/ very adequate
30. Has any staff member in your pharmacy ever tested positive for COVID19? [yes/no]
    a. How many staff members have ever tested COVID19 positive? [numeric]
31. State any other comments you have regarding COVID19 infection prevention at your workplace? [open-ended, leave blank if none]

SECTION V: VIEWS ABOUT CORONAVIRUS 2019 (COV19) TESTING IN PHARMACIES

32. Do you get clients asking for COV19 testing? Yes/no
    a. In the last one week, how many clients asked for COV19 testing? [numeric]
    b. What help do you offer clients asking for COV19 testing? COV19 tests/ Temperature measurement/ Referral/ other
        i. If referral, where do you refer? [can select more than one]
            Another pharmacy
            Public clinic/ hospital
            Private clinic/ hospital
            Private lab
            Other, specify
33. [If providing COV19 testing] What type of tests do you provide? [can select more than one]
    Antibody rapid tests, specify [unit cost]
    Antigen rapid tests, specify [unit cost]
    Laboratory tests, specify [unit cost]
    Other, specify [unit cost]
34. [If not currently providing] Do you think COV19 testing should be offered in pharmacies? Yes/no
    a. If yes, state the main reason for supporting pharmacy-based COV19 testing [select one]
        There is demand for it/ clients ask for it
        Clients shy away from health facilities
        Clients perceive pharmacies as more confidential compared to other testing facilities
        Pharmacies are open for longer hours
        Pharmacies are easily accessible
        Pharmacies provide speedier (faster) services
        Other, specify
    b. If no, state the main reason for NOT supporting pharmacy-based COV19 testing
        There is no demand for it
        No privacy in pharmacies
        Might be misused
        Other, specify
35. State any other comments you have regarding provision of COV19 testing in retail pharmacies? [open-ended, leave blank if none]

SECTION VI: OTHER VIEWS AND EXPERIENCES

36. How prepared do you feel about handling a client who presents to the pharmacy with COVID19 symptoms? [select one]
    Very unprepared/ unprepared/ prepared/ very prepared
37. Which is the main concern you are facing related to personal health and wellbeing? [select one]
    None
    Exposure to infection at work

For peer review only - http://bmjopen.bmj.com/site/about/guidelines.xhtml

Lack of transportation to get to work
Being caught up with the curfew
Keeping my family safe
Meeting financial needs for me and my family
Other, specify

38. How much do you feel your personal role as a health worker in the COVID19 response is valued by the community you are serving?
    Not at all valued/ little valued/ highly valued/ very highly valued
39. What other inputs can pharmacies (uniquely) contribute to the national response to the epidemic?? [open-ended]
40. State any other comments you have regarding pharmacy practice during the COVID19 pandemic? [open-ended, leave blank if none]

For peer review only

For peer review only - http://bmjopen.bmj.com/site/about/guidelines.xhtml

Supplementary file B - Simulated client script

Call-in simulation

- Look up the pharmacy on Google Maps and note basic operational details, such as opening hours or COVID19-related client interactions. Take note of any COVID-related messaging.
- Call the customer service number on the Google Maps profile, or if no profile or no number provided, try a simple google search, otherwise contact the study coordinators. They will check if the number in the database is personal or official; if personal further discussion is needed to assess risk of detection.
- **Ask if they have medicines that you can use to protect yourself from getting COVID19, using appropriate language** as agreed during the training. Avoid prompting for information unless it is in the script.
- Make a mental note of **questions asked or advice given**. Encourage the provider to talk by listening actively and saying "don't know" if the provider asks, for example, if you know how to prepare ginger home remedy.
    - If asked why you are asking for the medicines, say that your job requires interaction with customers, some of who don't wear masks
    - If asked where you are located, mention a name of a place near the pharmacy
- If any products are recommended, ask and **write down the names.** You can also ask for the price to keep the conversation natural, but the price data is not being collected
- Ask the service provider **how you can get the medicines**. List down the **options given**, and then if "pick up from the pharmacy" is one of the options, mention that you would rather not visit the pharmacy physically, because you are afraid of getting exposed to COVID19.
- If not told spontaneously,
    - ask what **symptoms you should look out for** if you suspect COVID19 infection? If asked what symptoms you have, emphasize you don't have symptoms but just want to be ready.
    - ask **what you should do if you suspect you have COVID19 infection**?
- Thank the service provider and mention that you will get back to them to arrange how to get the medicines.

Completing the debrief questionnaire

- The debrief questionnaire should ideally be completed within 30 minutes of the simulation, while memory is still fresh. In any case, do not call another pharmacy before the previous debrief interview has been done.
- It is **very important to say honestly how the call went**, even if you feel like it didn't go very well. The **study is assessing the pharmacy and not you**, the simulated client.
- Try to capture the responses to open-ended questions close as possible to what the provider says (verbatim)
- If there is follow-up interaction, you can leave the questionnaire "unsubmitted" and finish later. Open a fresh page if you need to do another pharmacy
- If further information comes in after you have submitted, relay to the study coordinators who will then edit the record from the back end.

Ver 1.2; 08 dec 2020

For peer review only

Supplementary file C - debrief questionnaire

Basic data

- Pharmacy identifier
- Simulated client identifier
- Simulation type: in-person/ call-in

Call-in simulation

- What time did you call the pharmacy? [24-hour format]
- What was the gender of the person who served you? Male/ female/ could not tell
- Were any medicines recommended? Yes/no
- If yes, which medicines were recommended for prevention? [Indicate price for each]
    - None
    - Vitamin C
    - Zinc
    - Multivitamins
    - Azithromycin,
    - Other antibiotics (Augmentin, Amoxil, ceftriaxone, etc)
    - Herbal medicine, specify
    - Home remedies, specify
    - Other, specify
- What options were given on how you can get the recommended medicines?
    - None
    - Come to the pharmacy and pick
    - Send someone to the pharmacy to pick
    - The pharmacy to deliver to client's location
    - From nearest chemist/ health facility
    - Other, specify
- If no medicines recommended, what was recommended?
    - None
    - Mask use
    - Hand hygiene (washing, sanitizer)
    - Other, specify
- Were you asked why you are asking for the medicines? (yes/no)
- Before prompting, were you told the symptoms to look out for if you suspect COVID19 infection? Yes/no
    - If yes, describe [open-ended]
- After prompting, what symptoms were you told to look out for if you suspect COVID19 infection? [open-ended]
- Before prompting, were you told what to do if you suspect you have COVID19 infection? Yes/no
    - If yes, what was recommended if you suspect COVID19 infection?
        - Medications
        - Visit nearest health facility/ go see a doctor
        - Check for information online/ WHO website
        - Get tested for COVID19
        - Get checked for oxygen level
        - Get checked for body temperature
        - Self-quarantine/ isolate at home
        - other, specify
    - If medications, what medications were recommended for treatment?
        - Steriods (dexamethasone, betamethasone, prednisolone, etc)
        - Azithromycin,

Ver 1.3; 08 dec 2020

For peer review only - http://bmjopen.bmj.com/site/about/guidelines.xhtml

    - Other antibiotics (Augmentin, Amoxil, ceftriaxone, etc)
    - Painkillers (paracetamol, brufen, etc)
    - Herbal medicine, specify
    - Home remedies, specify
    - Other, specify
- After prompting, what were you told to do if you suspect you have COVID19 infection? Medications
    - Nothing
    - Visit nearest health facility/ go see a doctor
    - Check for information online/ WHO website
    - Get tested for COVID19
    - Get checked for oxygen level
    - Get checked for body temperature
    - Self-quarantine/ isolate at home
    - other, specify
  - If medications, what medications were recommended for treatment?
    - Steriods (dexamethasone, betamethasone, prednisolone, etc)
    - Azithromycin,
    - Other antibiotics (Augmentin, Amoxil, ceftriaxone, etc)
    - Painkillers (paracetamol, brufen, etc)
    - Herbal medicine, specify
    - Home remedies
    - Other, specify
- What other advice was given?
    - None
    - Wear a mask
    - Sanitize/ wash hands
    - Eat fruits
    - Other, specify
- What other questions were you asked? [open-ended]
- About how long was the call? [mins]
- Was there further interaction after the call? Yes/no
  - If yes, describe
    - SMS
    - Whatsapp
    - Follow-up call
    - Other, specify

<u>General observations</u>

- How friendly (approachable) was the person (s) who served you?
  1 – Very unfriendly
  2 – Unfriendly
  3 – Friendly
  4 – Very friendly
- How enthusiastic was the provider about providing the service?
  1 – Very unenthusiastic
  2 – Unenthusiastic
  3 – Enthusiastic
  4 – Very enthusiastic
- Did the provider say or do anything that you found particularly helpful or nice? Yes/no
  If yes, describe [free text]
- Did the provider say or do anything that you found particularly NOT helpful or NOT nice? Yes/no
  If yes, describe [free text]

Ver 1.3; 08 dec 2020

For peer review only

- Do you have any additional observations about the interaction? Yes/no
  If yes, describe [free text]

For peer review only

For peer review only - http://bmjopen.bmj.com/site/about/guidelines.xhtml

Supplementary file D - In-depth Interview Topic Guide:

Assessing the response to the COVID-19 epidemic by private retail pharmacies in Kenya.

Notes to interviewer:

1. As first option, use Teams platform with video on. If internet is slow, try Teams without video and if that still doesn't work then use plain old telephone (POT).
2. If using POT, put the phone on speaker and record on a standard audio recorder placed next to the phone, and if using Teams, record directly through the application.
3. When setting up the interview, ask about internet access, preference of platform, etc. Inform the participant that they will need to be in a quiet place for about 30-60 minutes and that earphones are preferred to speaker mode.
4. Information required from the questionnaire:
    a. Preparedness level in handling COVID suspect case
    b. Products provided
    c. Information and counseling provided
    d. Whether they get clients who ask for testing
    e. Whether they think testing can be offered in a pharmacy

We would like to understand how the pharmacy sector is responding to the COVID19 epidemic, including: initial impact, experiences providing services during the epidemic, and views about pharmacy-based COVID19 testing. Towards the end of the interview I will also ask you a few questions about your experience being interviewed remotely compared to face-to-face.

Interviewee profile

1. Briefly tell me about your pharmacy (setting, staff, clients)?
    - What is your role in the pharmacy?

Initial response and impact

2. When did you first hear about COVID19? From who?
    - What did you think and feel about it at that time?

3. What was the initial response at your pharmacy?

4. What was the effect of the epidemic on pharmacy operations?
    - Client flow, sales, working hours, staff, meeting routine, etc

5. What has been your role in responding to the epidemic?
    - You said your preparedness in handling a COVID-19 case was: ________________. Please explain further
    - What aspects of your education and previous experience helped you in responding to the outbreak?

COVID-related products and services

6. In the questionnaire, you mentioned a number of products that your pharmacy is providing in relation to the epidemic. ____
   Which of these products were introduced specifically because of the epidemic?
    - You also mentioned information and counseling that clients are seeking or you are providing? ____
    - How is this done?
    - How does your pharmacy handle suspected COVID19 cases?

7. Have you been involved in any COVID training or peer learning sessions, either as a participant or a trainer?

Version 1.4; 19feb2021

For peer review only - http://bmjopen.bmj.com/site/about/guidelines.xhtml

- If yes, Where? When? Who else was included?
  - Was it stand alone training on this topic or part of a wider training?
  - Do you feel the training was adequate?
  - Who was supporting the training (financially and technically)?
- If no, how did you learn how to provide COVID services and products?

8. What do you think is needed to improve the delivery of COVID-related services in the retail pharmacy sector?
   - What support (material or financial) can be given by the government or any other external party? (e.g., drugs and other supplies/ toolkits/ job-aids/ digital systems)
   - Do you report information on COVID-related services to the government or any other external party?

<u>Pharmacy-based COVID19 testing</u>

9. If they get clients who ask for COVID19 testing: YES NO (*circle one*)
   - What type of clients ask for testing? (reasons for wanting to test, etc)
   - How do you help clients asking for COVID19 testing?
   - Why do you think they prefer pharmacies rather than health facilities or other testing sites?

10. You mentioned that COVID19 testing SHOULD/ SHOULD NOT *(circle one)* be provided in pharmacies? Explain.
    - What would be needed to ensure successful implementation of COV19 testing in the retail pharmacy sector?
    - What type of tests would be most suited for pharmacy-based COVID19 testing?

<u>Other potential inputs into the response</u>

11. What other inputs can pharmacies (uniquely) contribute to the national response to the epidemic?
    - How would this be done?

Conclusion of main interview: We have come to the end of the specific questions I had. Do you have any further comments?

<u>Experience doing the interview remotely</u>

Now I would like to ask you a few questions about your experience being interviewed remotely compared to face-to-face.

12. How would you describe your experience doing the interview remotely, compared to face-to-face?
    - Have you done a face-to-face interview before? Describe.
    - Advantages and challenges
    - Would you have preferred a face-to-face interview? Explain.

13. What did you like or not like about the Teams platform?
    - Do you have previous experience with digital meeting applications? (Teams, Zoom, etc)
    - Would you have preferred another platform?

Conclusion: Thank you very much for your time. If it is ok with you, I may contact you again for any clarifications or to hear about additional experiences and views on the topic. We will share a report once data analysis is complete.

For peer review only - http://bmjopen.bmj.com/site/about/guidelines.xhtml

Supplementary file E - Checklist – Consolidated criteria for reporting qualitative studies (COREQ)[i]

Manuscript: Response to the coronavirus disease 2019 (COVID-19) pandemic at private retail pharmacies in Kenya: a mixed methods study

Item number	Guide questions/description	Reported on Page #
**Domain 1: Research team and reflexivity**		
Personal Characteristics		
1. Inter viewer/facilitator	Which author/s conducted the interview or focus group?	4
2. Credentials	What were the researcher's credentials? E.g. PhD, MD	The names and affiliations are included on title page
3. Occupation	What was their occupation at the time of the study?	The names and affiliations are included on title page
4. Gender	Was the researcher male or female?	One female, one male
5. Experience and training	What experience or training did the researcher have?	The names and affiliations are included on title page
Relationship with participants		
6. Relationship established	Was a relationship established prior to study commencement?	4- Participants had been included in a previous study
7. Participant knowledge of the interviewer	What did the participants know about the researcher? e.g. personal goals, reasons for doing the research	5 – participants signed a consent form that explained the research goals
8. Interviewer characteristics	What characteristics were reported about the inter viewer/facilitator? e.g. Bias, assumptions, reasons and interests in the research topic	No bias to report
**Domain 2: study design**		
Theoretical framework		
9. Methodological orientation and Theory	What methodological orientation was stated to underpin the study? e.g. grounded theory, discourse analysis, ethnography, phenomenology, content analysis	5- thematic analysis
Participant selection		
10. Sampling	How were participants selected? e.g. purposive, convenience, consecutive, snowball	4- purposive
11. Method of approach	How were participants approached? e.g. face-to-face, telephone, mail, email	4- telephone
12. Sample size	How many participants were in the study?	5 – 18 participants
13. Non-participation	How many people refused to participate or dropped out? Reasons?	Participatin rate was 56% of 32 providers invited; 7 of those invited did not respond after three reminders, 5 said they were not interested, and 2 had already left the pharmacy

For peer review only

Item number	Guide questions/description	Reported on Page #
		Not included due to word limit
Setting		
14. Setting of data collection	Where was the data collected? e.g. home, clinic, workplace	4-phone
15. Presence of non-participants	Was anyone else present besides the participants and researchers?	No.<br><br>*Not included due to word limit*
16. Description of sample	What are the important characteristics of the sample? e.g. demographic data, date	5 – supp tab 2
Data collection		
17. Interview guide	Were questions, prompts, guides provided by the authors? Was it pilot tested?	4 – supp file D<br><br>The interview guide was updated after the first few interviews based on responses from participants and how effectively these addressed research objectives.<br><br>*Not included due to word limit*
18. Repeat interviews	Were repeat interviews carried out? If yes, how many?	No repeat interviews were done.<br><br>*Not included due to word limit*
19. Audio/visual recording	Did the research use audio or visual recording to collect the data?	5 – data management and analysis
20. Field notes	Were field notes made during and/or after the interview or focus group?	Field notes made after the interviews<br><br>*Not included due to word limit*
21. Duration	What was the duration of the interviews or focus group?	Interviews lasted a median [range] of 35 [20-92] minutes<br><br>*Not included due to word limit*
22. Data saturation	Was data saturation discussed?	To expedite data collection, all 18 interviews were completed before starting analysis or checking for saturation<br><br>*Not included due to word limit*
23. Transcripts returned	Were transcripts returned to participants for comment and/or correction?	Transcripts were not returned to participants for comment and/or correction

For peer review only

Item number	Guide questions/description	Reported on Page #
		Not included due to word limit
**Domain 3: analysis and findings**		
Data analysis		
24. Number of data coders	How many data coders coded the data?	5 – data management and analysis
25. Description of the coding tree	Did authors provide a description of the coding tree?	No.<br><br>*Not included due to word limit*
26. Derivation of themes	Were themes identified in advance or derived from the data?	Analysis followed a thematic approach with initial categories based on the interview guide and emergent themes integrated<br><br>*Not included due to word limit*
27. Software	What software, if applicable, was used to manage the data?	5- NVivo 12
28. Participant checking	Did participants provide feedback on the findings?	Participants were not asked for feedback on the findings.<br><br>*Not included due to word limit*
Reporting		
29. Quotations presented	Were participant quotations presented to illustrate the themes/findings? Was each quotation identified? e.g. participant number	Yes. Results section
30. Data and findings consistent	Was there consistency between the data presented and the findings?	Yes.
31. Clarity of major themes	Were major themes clearly presented in the findings?	Yes. Results section
32. Clarity of minor themes	Is there a description of diverse cases or discussion of minor themes?	Yes. Results section

For peer review only

[i] Developed from:
Tong A, Sainsbury P, Craig J. Consolidated criteria for reporting qualitative research (COREQ): a 32-item checklist for interviews and focus groups. *International Journal for Quality in Health Care*. 2007. Volume 19, Number 6: pp. 349 – 357

STROBE Statement—Checklist of items that should be included in reports of ***cross-sectional studies***

	Item No	Recommendation	Page No
**Title and abstract**	1	(*a*) Indicate the study's design with a commonly used term in the title or the abstract	1, 2
		(*b*) Provide in the abstract an informative and balanced summary of what was done and what was found	2
**Introduction**			
Background/rationale	2	Explain the scientific background and rationale for the investigation being reported	2-3
Objectives	3	State specific objectives, including any prespecified hypotheses	3
**Methods**			
Study design	4	Present key elements of study design early in the paper	3
Setting	5	Describe the setting, locations, and relevant dates, including periods of recruitment, exposure, follow-up, and data collection	3
Participants	6	(*a*) Give the eligibility criteria, and the sources and methods of selection of participants	4
Variables	7	Clearly define all outcomes, exposures, predictors, potential confounders, and effect modifiers. Give diagnostic criteria, if applicable	4
Data sources/ measurement	8*	For each variable of interest, give sources of data and details of methods of assessment (measurement). Describe comparability of assessment methods if there is more than one group	*4*
Bias	9	Describe any efforts to address potential sources of bias	4-5
Study size	10	Explain how the study size was arrived at	4
Quantitative variables	11	Explain how quantitative variables were handled in the analyses. If applicable, describe which groupings were chosen and why	4-5
Statistical methods	12	(*a*) Describe all statistical methods, including those used to control for confounding	5
		(*b*) Describe any methods used to examine subgroups and interactions	5
		(*c*) Explain how missing data were addressed	5
		(*d*) If applicable, describe analytical methods taking account of sampling strategy	5
		(*e*) Describe any sensitivity analyses	5
**Results**			
Participants	13*	(a) Report numbers of individuals at each stage of study—eg numbers potentially eligible, examined for eligibility, confirmed eligible, included in the study, completing follow-up, and analysed	5
		(b) Give reasons for non-participation at each stage	5
		(c) Consider use of a flow diagram	n/a
Descriptive data	14*	(a) Give characteristics of study participants (eg demographic, clinical, social) and information on exposures and potential confounders	5
		(b) Indicate number of participants with missing data for each variable of interest	5
Outcome data	15*	Report numbers of outcome events or summary measures	5
Main results	16	(*a*) Give unadjusted estimates and, if applicable, confounder-adjusted estimates and their precision (eg, 95% confidence interval). Make clear which confounders were adjusted for and why they were included	6-17

For peer review only

		(*b*) Report category boundaries when continuous variables were categorized	6-17
		(*c*) If relevant, consider translating estimates of relative risk into absolute risk for a meaningful time period	6-17
Other analyses	17	Report other analyses done—eg analyses of subgroups and interactions, and sensitivity analyses	6-17
**Discussion**			
Key results	18	Summarise key results with reference to study objectives	17-20
Limitations	19	Discuss limitations of the study, taking into account sources of potential bias or imprecision. Discuss both direction and magnitude of any potential bias	20
Interpretation	20	Give a cautious overall interpretation of results considering objectives, limitations, multiplicity of analyses, results from similar studies, and other relevant evidence	17-20
Generalisability	21	Discuss the generalisability (external validity) of the study results	20
**Other information**			
Funding	22	Give the source of funding and the role of the funders for the present study and, if applicable, for the original study on which the present article is based	20-21

*Give information separately for exposed and unexposed groups.

Note: An Explanation and Elaboration article discusses each checklist item and gives methodological background and published examples of transparent reporting. The STROBE checklist is best used in conjunction with this article (freely available on the Web sites of PLoS Medicine at http://www.plosmedicine.org/, Annals of Internal Medicine at http://www.annals.org/, and Epidemiology at http://www.epidem.com/). Information on the STROBE Initiative is available at www.strobe-statement.org.

For peer review only - http://bmjopen.bmj.com/site/about/guidelines.xhtml

BMJ Open

Experiences and response to the coronavirus disease 2019 (COVID-19) pandemic at private retail pharmacies in Kenya: a mixed methods study

Journal:	*BMJ Open*
Manuscript ID	bmjopen-2021-058688.R1
Article Type:	Original research
Date Submitted by the Author:	23-Feb-2022
Complete List of Authors:	Mugo, Peter; KEMRI-Wellcome Trust Research Programme Nairobi, Health Economics Research Unit; KEMRI-Wellcome Trust Research Programme, Health Systems and Research Ethics Department<br>Mumbi, Audrey; KEMRI-Wellcome Trust Research Programme Nairobi, Health Economics Research Unit<br>Munene, Daniella; Pharmaceutical society of Kenya, CEO Office<br>Nzinga, Jacinta; KEMRI-Wellcome Trust Research Programme Nairobi, Health Economics Research Unit; KEMRI-Wellcome Trust Research Programme, Health Systems and Research Ethics<br>Molyneux, Sassy ; KEMRI-Wellcome Trust Research Programme, Health Systems and Research Ethics; University of Oxford, Nuffield Department of Medicine<br>Barasa, Edwine; KEMRI-Wellcome Trust Research Programme Nairobi, Health Economics Research Unit; University of Oxford, Nuffield Department of Medicine<br>Taskforce, COVID19 ; Pharmaceutical Society of Kenya (PSK), COVID-19 Response Taskforce
**Primary Subject Heading**:	Public health
Secondary Subject Heading:	Global health, Health services research, Infectious diseases
Keywords:	COVID-19, Public health < INFECTIOUS DISEASES, PRIMARY CARE, PUBLIC HEALTH, QUALITATIVE RESEARCH, Quality in health care < HEALTH SERVICES ADMINISTRATION & MANAGEMENT

SCHOLARONE™
Manuscripts

I, the Submitting Author has the right to grant and does grant on behalf of all authors of the Work (as defined in the below author licence), an exclusive licence and/or a non-exclusive licence for contributions from authors who are: i) UK Crown employees; ii) where BMJ has agreed a CC-BY licence shall apply, and/or iii) in accordance with the terms applicable for US Federal Government officers or employees acting as part of their official duties; on a worldwide, perpetual, irrevocable, royalty-free basis to BMJ Publishing Group Ltd ("BMJ") its licensees and where the relevant Journal is co-owned by BMJ to the co-owners of the Journal, to publish the Work in this journal and any other BMJ products and to exploit all rights, as set out in our licence.

The Submitting Author accepts and understands that any supply made under these terms is made by BMJ to the Submitting Author unless you are acting as an employee on behalf of your employer or a postgraduate student of an affiliated institution which is paying any applicable article publishing charge ("APC") for Open Access articles. Where the Submitting Author wishes to make the Work available on an Open Access basis (and intends to pay the relevant APC), the terms of reuse of such Open Access shall be governed by a Creative Commons licence – details of these licences and which Creative Commons licence will apply to this Work are set out in our licence referred to above.

Other than as permitted in any relevant BMJ Author's Self Archiving Policies, I confirm this Work has not been accepted for publication elsewhere, is not being considered for publication elsewhere and does not duplicate material already published. I confirm all authors consent to publication of this Work and authorise the granting of this licence.

For peer review only

Experiences and response to the coronavirus disease 2019 (COVID-19) pandemic at private retail pharmacies in Kenya: a mixed methods study

Short Title: Pharmacy COVID-19 Response

Peter Mugo[1,3]*, Audrey Mumbi[1], Daniella Munene[2], Jacinta Nzinga[1,3], Sassy Molyneux[3,4], Edwine Barasa [1,3,4], on behalf of PSK CRT[5]

[1] Health Economics Research Unit, Kemri-Wellcome Trust Research Programme, Nairobi, Kenya
[2] Pharmaceutical society of Kenya
[3] Health Systems and Research Ethics Department, KEMRI-Wellcome Trust Research Programme, Kilifi, Kenya
[4] University of Oxford, Headington, UK
[5] Pharmaceutical Society of Kenya (PSK) COVID-19 Response Taskforce (CRT)

*Corresponding author:
Peter Mwangi Mugo
KEMRI-Wellcome Trust Research Programme
P.O. Box 43640 – 00100 Nairobi, Kenya
Tel: + 254 730 162 000
E-mail: PMugo@kemri-wellcome.org

Key words: Private retail pharmacies, COVID-19 response, Kenya

For peer review only

Abstract

Objectives: To assess experiences and response to the COVID-19 pandemic at community pharmacies in Kenya.

Design, setting, and participants: This was a mixed-methods study conducted from November 2020 to April 2021, targeting service providers in three counties (Nairobi, Mombasa, and Kisumu), selected purposively to represent the main urban centres; pharmacies were selected randomly from a list of licensed pharmacies.

Results: Of 195 sampled pharmacies, 108 (55%) completed a questionnaire and 103 (53%) received a simulated client call; 18 service providers were interviewed. The initial weeks of the pandemic were characterized by fear and panic among service providers and a surge in client flow. Subsequently, 65 (60%) of 108 pharmacies experienced a dip in demand to below pre-pandemic levels and 34 (31%) reported challenges with unavailability, high price, and poor-quality of products. Almost all pharmacies were actively providing preventive materials and therapies; educating clients on prevention measures; counselling anxious clients; and handling and referring suspect cases. Fifty-nine pharmacies (55% [95% CI 45-65%]) reported receiving a client asking for COVID-19 testing and a similar proportion stated they would support pharmacy-based testing if implemented. For treatment of simulated clients, most pharmacies (71%, 73 of 103) recommended alternative therapies and nutritional supplements such as vitamin C; the rest recommended conventional therapies such as antibiotics. While 52 (48%) of 108 pharmacies had at least one staff member trained on COVID-19, a general feeling of disconnection from the national program prevailed.

Conclusions: Private pharmacies in Kenya were actively contributing to the COVID-19 response, but more deliberate engagement, support and linkages are required. Notably, there is an urgent need to develop guidelines for pharmacy-based COVID-19 testing, a service that is clearly needed and which could greatly increase test coverage. Pharmacy-based COVID-19 programs should be accompanied with implementation research to inform current and future pandemic responses.

Strengths and limitations

- The study used multiple quantitative and qualitative methods to enhance the comprehensiveness and reliability of the findings.
- Of note, we used simulated clients to objectively assess provider practices, an approach that is gaining acceptance as a gold standard for the measurement of clinical practice quality around the world.

For peer review only - http://bmjopen.bmj.com/site/about/guidelines.xhtml

- The study took advantage of information collected in a recent study to speed up recruitment and data collection.
- One weakness of the study was the low response rate (55%), considering that it targeted pharmacies that had participated in a similar study less than a year previously.

Introduction

The coronavirus disease 2019 (COVID-19) pandemic is an ongoing global health emergency [1, 2]. As of September 2021, over 230 million cases had been confirmed globally resulting in over 4.7 million deaths [3]. For the better part of 2020, non-pharmaceutical interventions such as early case detection, contact reduction, and hygiene measures were the main prevention strategies [4, 5]. In early 2021, following unprecedented public-private collaboration and speed in research and development, a number of highly effective vaccines became available [6, 7].

Non-pharmaceutical supportive care remains the key approach to treatment and management [8-10]. Among hospitalized patients, pharmaceuticals that have been shown to be effective include corticosteroids such as dexamethasone, antivirals such as remdesivir, and immunomodulators such as tocilizumab [8, 11, 12]. In the outpatient setting, pharmaceuticals used to manage mild COVID-19 cases include those targeting fever and pain such as ibuprofen, and those aiming to relieve respiratory congestion [9][12]. Other more specific agents that have been tried, such as hydroxychloroquine, ivermectin, and azithromycin, are discouraged [9, 12]. The World Health Organization and the US National Institutes of Health recommend that such unproven therapies be used only within the context of clinical trials [13, 14].

It is predicted that follow-up waves of COVID-19 infection may continue indefinitely [15]. Strategies are therefore required to enhance and sustain coverage, cost-effectiveness, and efficiency of prevention and treatment interventions. Private retail pharmacies (also referred to as community pharmacies) are a unique channel through which COVID-19 prevention and treatment interventions can be delivered, since they are often the first or only point of contact with the healthcare system in developing countries [16-18]. The main reasons for preferential care-seeking at pharmacies, compared to health facilities, include greater accessibility, lower cost, and greater perceived privacy [19, 20]. The International Pharmaceutical Federation highlights key areas where the pharmacy sector can support the pandemic response, including: management of the medication supply chain, patient education, identification and referral of patients to other health care providers, and provision of prevention materials and therapies [21, 22]. During the COVID-19 pandemic, pharmacies in many settings, including Kenya, were categorized as essential services hence they were exempted from closure [23].

The first COVID-19 case in Kenya was reported on 13th March 2020, and as of 12th October 2021, there were 251,248 confirmed cases and 5,190 deaths [24]. A number of control measures have been instituted by the government at different times depending on rates of infection and mortality [23, 25, 26]. Vaccine roll-out in Kenya started in March 2021 [27]. The aim of the study

For peer review only - http://bmjopen.bmj.com/site/about/guidelines.xhtml

was to assess the preparedness, experiences, and response to the COVID-19 pandemic at private retail pharmacies in Kenya, with an overall goal of identifying strategies for maximising the contribution of pharmacies to the national response.

Methods

Study design

We conducted a cross-sectional descriptive study, including a questionnaire survey, a simulated client survey, and in-depth interviews. A mixed-methods approach was used in data collection and analysis: insights from interviews were used to refine the survey tools and probe quantitative data; and survey data were used to refine the interview guide and qualitative data analysis. Given the physical distancing requirements at the time, data collection was done remotely.

Study settings and populations

Community pharmacies in Kenya are generally small to medium-sized businesses providing typical pharmacy services such as filling of prescriptions, over-the counter products, and point-of-care testing or self-testing kits for common diseases. While traditionally a fragmented market, the sector is starting to consolidate. In 2019, of 5,033 licensed pharmacies, 4,286 (85%) were independent (single branch) pharmacies, 594 (12%) belonged to small chain networks (2-5 branches), and 152 (3%) were part of large chain networks (6-41 branches) belonging to 13 companies [28].

The study was conducted in three counties (Nairobi, Mombasa and Kisumu), selected purposively to represent the main urban centres, where there was high concentration of pharmacies and COVID-19 cases. As of June 2020, the three counties together contributed 32% (1,602/5,033) of licensed pharmacies [28] and 78% (2,229/2,862) of reported COVID-19 cases [29] [24].

We targeted pharmacies that had participated in a pharmacy HIV prevention (PHP) study about a year previously. Pharmacies had been selected randomly from the list of licensed pharmacies. The sample size for the PhP study (n=195) had been calculated to provide 80% power to detect associations at the 5% significance level, assuming a binary dependent variable split at 70% and 50% across a binary predictor variable. The sample size achieved in the current study (n=108) gives 57% power for similar parameters and assumptions, and 80% power for a 75%-50% split. Respondents were pharmacists (bachelor's degree holders) and pharmaceutical technologists (diploma holders). Interview participants were purposively selected from among questionnaire respondents, targeting those that showed enthusiasm for the study through prompt and detailed responses. We aimed to increase variability of interview data by stratifying the sample by county, practice setting (urban vs. rural) and gender (male vs female).

Data collection

For peer review only

Data collection started in November 2020 (approximately seven months after the pandemic reached Kenya) and was concluded in December 2020 (for the surveys) and April 2021 (for the interviews).

We used previously obtained phone numbers to contact sampled pharmacies, obtain informed consent and share a link to an online questionnaire. One questionnaire, taking about 20-30 minutes, was completed by each participating pharmacy (**Supplementary file A**). Up to three reminder phone calls were made. Data on pharmacy characteristics were imported from the PHP study database.

Simulated clients (SCs) made unscheduled phone calls to participating pharmacies, mimicking a client seeking COVID-19 preventive therapies (**Supplementary file B**). The SCs (2 women and 2 men, aged 25-35 years) had previously been engaged in the PHP study and trained comprehensively over a 2-week period. For the current study, further training was provided over a 1-week period through the Teams® digital meeting platform. An online debrief questionnaire was used to record observations (**Supplementary file C**). All calls were made on weekdays (Monday – Friday) between 8am and 5pm, over a 3-week period. Median [range] duration of calls was 4 [1-12] minutes. The majority (68%) of calls were made to a publicly available phone number, while the rest used a number in the study database as there was no public number. A few weeks after the simulated call, participating pharmacies were contacted to check if they suspected any clients visiting the pharmacies during the survey period to be "fake" (detection survey).

In-depth interviews were conducted by the first and second authors using a semi-structured interview guide (**Supplementary file D**). Interviews were conducted via the Teams® digital meeting platform as a first option or a simple phone call if the participant so preferred or if internet connectivity was poor.

Data management and analysis

Quantitative data were captured directly into an open-source database (REDcap®, Vanderbilt University). Data cleaning and analysis was carried out using Stata® version 15 (College Station, Texas, USA). Summary statistics were compared across counties using the chi square test or analysis of variance for means. Exploratory multivariable modelling was done using logistic regression. Variables with $p<0.10$ in bivariate analysis were included in the multivariable model and those with $p<0.05$ in the multivariable model were considered to be statistically significant. Systematicity in detection was assessed by correlating detection status with caller, pharmacy characteristics, and simulation process features.

Qualitative interviews were audio-recorded and transcribed verbatim in Microsoft Word®, translating into English where necessary. To ensure quality, all transcripts were cross-checked against the audio recordings. Transcripts were imported into NVivo 12 (SR International,

For peer review only - http://bmjopen.bmj.com/site/about/guidelines.xhtml

Australia) and coded by the first and second authors. Analysis followed a thematic approach with initial categories based on the interview guide and emergent themes integrated in a second round of coding. A checklist on Consolidated criteria for Reporting Qualitative research (COREQ) was completed (**Supplementary file E**).

Ethical considerations

The study was approved by the Kenya Medical Research Institute (KEMRI) Scientific and Ethics Review Unit (KEMRI/ SERU/CGMR-C/208/4080). Participants signed written informed consent forms administered digitally.

Results

Response rate and characteristics of participants

Of 195 target pharmacies, 108 (55%) completed a questionnaire. Participating pharmacies were generally similar to non-participating pharmacies, but had features indicative of higher operational capacity and interest in public health interventions, such as having a computerized stock management system, written job descriptions, and providing blood pressure measurement (data not shown). Ninety-four (87%) pharmacies were located in urban areas; 44 (41%) had a consultation room; 71 (67%) had a computerised stock management system; and 13 (12%) were part of a chain network **(Supplementary table I)**. The majority of questionnaire respondents were male, below 40 years, and had a pharmacy diploma.

One hundred and three (95%) pharmacies received a simulated client call. Thirteen (13%) calls were determined as probably detected. Detection was less likely if the call was made by one particular caller (perfect prediction), gender of person answering the call was male (adjusted odds ratio, aOR, 0.1, p=0.01); the pharmacy had a publicly available phone number (aOR 0.01, p=0.01), was part of a chain network (perfect prediction), opened 24 hours daily (perfect prediction), or had an on-site laboratory (perfect prediction). Main call outcomes (preventive therapies recommended, options for delivery of recommended products, and referral options) did not vary by detection status (data not shown), therefore all data were included in analysis.

Of 18 interview participants, 11 (61%) were male, 16 (88%) were below 40 years, 11 (61%) were pharmacy owner or in-charge, and 12 (67%) were diploma holders. Number of participants was balanced by county (6 each), but was skewed towards urban setting (13 v 5) and male gender (11 v 7) (**Supplementary Table II)**.

Impact of the pandemic on service providers and pharmacy operations

Interview participants described fear and panic when they first heard about COVID-19, especially regarding the risk of exposure to infection:

> *"In the beginning, it sent everybody into a panic mode. Including my entire staff where everybody now thought this is a killer disease and being healthcare workers, they must be people who will be among the victims."*

For peer review only

- Male pharmaceutical technologist, Nairobi, 105

Some providers were in denial, feeling that the pandemic would never reach *"where we are"*, and others reported that some clients believed the pandemic was "*just an avenue maybe for our politicians ready to make some money or something*".

The main concerns reported in the questionnaire were: exposure to infection at work (66% of respondents), "keeping my family safe" (22%), meeting financial needs (6%), and being caught up in the curfew (3%).

One hundred (93%) of 108 pharmacies reported pandemic-induced operational challenges (**Figure 1**). The main supply-side challenge was unavailability and high price of products and the main demand-side challenge was reduced client flow.

Figure 1. Challenges experienced by pharmacies during the COVID-19 pandemic in Kenya

Interview data corroborated survey findings. Interviewees described temporal changes in client flow, with an initial surge of clients buying vitamins and other immune boosters, followed a few weeks later by a dip in demand to below pre-pandemic levels. The initial surge was accompanied by an increase in self-medication and stockpiling of long-term medications, such as for diabetes. While some of these requests were deemed potentially inappropriate, providers were not always able to effectively intervene:

> *"They just keep [these drugs] in case. …Like there was one [client] who came with a running nose, and then he was telling me he wanted Zithromax [a brand of the antibiotic azithromycin]. 'Get Flu gone, get antihistamine, this one will help you' [I told him]. So sometimes as a primary healthcare provider, you try to advise them, but now they have made up their mind, so you just go ahead and let them go [somewhere else]. Because whether you refuse, you do what, they've made up their mind."*
>
> - Male pharmaceutical technologist, Kisumu, 303

Participants attributed the subsequent reduction in demand mainly to the dusk-to-dawn curfew, despite the fact that pharmacy providers were classified as essential workers and issued curfew passes:

> *"Of course, the curfew really beat us down, because now by the time [it's] seven o'clock, people are going home… and it's our prime time to serve clients as from 7 pm. So, we as well missed out on business."*
>
> - Male pharmacy owner, Mombasa, 203

A number of interviewees believed that the decrease in service utilization was due to fear among clients, of infection and quarantine:

For peer review only - http://bmjopen.bmj.com/site/about/guidelines.xhtml

"Then people shied off from coming to the pharmacy especially the ones with coughs and flus because they were just thinking that I could tell them to go to hospital for the corona tests. So most clients decided to stay at home, not to come to the pharmacy for consultation, or they just sent somebody to buy them the drugs"

- Female pharmaceutical technologist, Kisumu, 304

One interviewee saw the stockpiling by clients in positive light, observing that it enabled him to offload slow-moving products:

"People resorted to scavenging where they might find these products and so that way [one was] able to liquidate what was lying in your shops"

- Male pharmaceutical technologist, Nairobi, 105

A reduction in hygiene-related illnesses was also hailed as a positive public health effect of the COVID-19 preventive measures:

"Due to proper maintenance of hygiene, the short-short illnesses have reduced. Washing of hands and stuff.""

- Male pharmacist, Nairobi, 103

Adjustments to pharmacy operations

Pharmacies adjusted their operations to cope with these challenges and to accommodate the government-instituted prevention measures: 74% reduced opening hours (mean 3 hours [range 1-7]); 38% reduced staffing; and 30% changed their meeting routine (**Table 1**). Adjustments did not vary significantly across counties.

Table 1. Adjustments to pharmacy operations following the outbreak of COVID-19 in Kenya

Change	Nairobi (N=38)		Mombasa (N=36)		Kisumu (N=34)		Total (N=108)	
	n	%	n	%	n	%	n	%
**Opening hours**[1]								
No change	7	18%	8	22%	10	29%	25	23%
Reduced open hours per day	31	82%	25	69%	24	71%	80	74%
Reduced open days per week	1	3%	4	11%	0	0%	5	5%
Closed temporarily	0	0%	0	0%	1	3%	1	1%
**Staffing**								
No change	19	50%	25	71%	19	59%	63	60%
Reduced number of staff	18	47%	9	26%	13	41%	40	38%
Increased number of staff	0	0%	1	3%	0	0%	1	1%
Reduced pay	1	3%	0	0%	0	0%	1	1%
**Stock volume levels**								
No change	12	32%	13	36%	8	24%	33	31%
Reduced overall	8	21%	12	33%	12	35%	32	30%
Increased overall	14	37%	10	28%	14	41%	38	35%

For peer review only

Change	Nairobi (N=38)		Mombasa (N=36)		Kisumu (N=34)		Total (N=108)	
Both reduced and increased for different items	4	11%	1	3%	0	0%	5	5%
**Meeting routine**								
No change	24	63%	30	83%	22	65%	76	70%
Resorted to virtual/ remote/ online meetings[2]	8	21%	3	8%	7	21%	18	17%
Reduced frequency of meetings	4	11%	1	3%	3	9%	8	7%
Stopped meetings altogether	2	5%	1	3%	0	0%	3	3%
Reduced duration/ number of people	0	0%	1	3%	1	3%	2	2%
Changed timing	0	0%	0	0%	1	3%	1	1%

[1] Three pharmacies in Mombasa and one in Nairobi reduced their open days per week by one, while one pharmacy in Mombasa reduced by three days. One pharmacy in Kisumu closed temporarily for 2 weeks
[2] Main virtual platforms used included Zoom, Google Meet, and Whatsapp

COVID-19-related products and services provided by pharmacies

Table 2 presents the COVID-19-related products and services that were being provided or requested at pharmacies. Equipment and materials in highest demand were medical masks, N95/ K95 respirators, face shields, hand sanitizer, and thermometers; while the medications in highest demand were vitamin C, multivitamins, and antibiotics. Only 24 (22%) pharmacies reported keeping records related to COVID-19 services.

Table 2. COVID-19-related products and services provided by private retail pharmacies in Kenya. Data are number (%) of pharmacies providing or receiving requests (main row) and number of clients in past week (subsidiary row).

Product/ service	Nairobi (N=38)		Mombasa (N=36)		Kisumu (N=34)		Total (N=108)	
	N or median	% or IQR	N or median	% or IQR	N or median	% or IQR	N or median	% or IQR
**Equipment and materials**								
Cloth masks	1	3%	5	14%	6	18%	12	11%
Number of clients in past week	20	20-20	20	4-30	10	5-15	15	5-25
Medical Masks	37	97%	33	92%	30	88%	100	93%
Number of clients in past week	50	20-100	23	10-80	26	20-50	30	20-70
N95/ K95 masks	24	63%*	18	50%	11	32%	53	49%
Number of clients in past week	5	4-16	5	2-30	1	0-2	5	2-10
Goggles	6	16%	2	6%	3	9%	11	10%
Number of clients in past week	0	0-10	6	2-10	1	0-5	1	0-10
Face shields	19	50%*	10	28%	8	24%	37	34%
Number of clients in past week	1	0-6	1	0-3	1	0-8	1	0-5
Gowns/ coveralls	1	3%	1	3%	3	9%	5	5%
Number of clients in past week	0	0-0	10*	0-10	0	0-1	0	0-1

For peer review only - http://bmjopen.bmj.com/site/about/guidelines.xhtml

Product/ service	Nairobi (N=38)		Mombasa (N=36)		Kisumu (N=34)		Total (N=108)	
	N or median	% or IQR	N or median	% or IQR	N or median	% or IQR	N or median	% or IQR
Hand sanitizer	37	97%	35	97%	31	91%	103	95%
Number of clients in past week	10	3-30	5	2-13	5	2-13	5	3-15
Thermometers	27	71%	23	64%	20	59%	70	65%
Number of clients in past week	2	1-5	2	1-5	1	0-2	2	2-3
Pulse oximeters	10	26%	11	31%	3	9%	24	22%
Number of clients in past week	1	1-3	2	0-10	1	0-2	2	1-3
**Medicines**								
Vitamin C[1]	35	92%*	29	81%	23	68%	87	81%
Number of clients in past week	10	10-30	15	10-30	5	3-11	10	4-20
Multivitamins[1]	29	76%	30	83%	24	71%	83	77%
Number of clients in past week	10	5-19	10	6-20	10	7-18	10	6-20
Traditional/ alternative/ natural remedies[1, 2]	1	3%	2	6%	0	0%	3	3%
Number of clients in past week	0	0-0	13	5-20	0	0-0	5	0-20
Hydroxychloroquine/ chloroquine	8	21%	7	19%	2	6%	17	16%
Number of clients in past week	3	2-7	1	0-5	3	0-5	2	0-5
Antibiotics[1, 3]	26	68%	22	61%	23	68%	71	66%
Number of clients in past week	3	2-10	15*	10-39	15	5-29	10	3-20
Steroids[1]	16	42%	16	44%	16	47%	48	44%
Number of clients in past week	3	1-8	9	5-19	10	4-15	7	3-15
Other medicines[4]	3	8%	4	11%	2	6%	9	8%
Number of clients in past week	3	1-5	28	23-45	3	1-4	13	3-28
**Information and counselling**								
How to protect themselves from infection	28	74%	28	78%	27	79%	83	77%
Number of clients in past week	5	2-13	5	3-15	10	3-20	5	3-15
Symptoms to look out for if they suspect infection	32	84%	28	78%	26	76%	86	80%
Number of clients in past week	3	2-10	5	4-10	9	2-11	5	2-11
What to do if they develop symptoms/ suspect infection	22	58%	21	58%	16	47%	59	55%
Number of clients in past week	3	2-7	3	2-5	5	3-22	4	2-10
Mask use and related problems	19	50%	19	53%	20	59%	58	54%
Number of clients in past week	5	2-10	5	2-35	15	4-20	7	2-20
Counselling for anxiety related to getting infected/ becoming sick with COVID-19	14	37%	16	44%	13	38%	43	40%
Number of clients in past week	2	1-10	3	1-10	3	1-5	3	1-8
Counselling for anxiety about job/ business loss	5	13%	7	19%	5	15%	17	16%
Number of clients in past week	2	2-8	2	1-5	5	3-8	3	2-8
**Modes of communication with clients**								
Face-to-face at the pharmacy	33	87%	33	92%	34	100%	100	93%
Phone calls (including WhatsApp calls etc)	21	55%	25	69%	20	59%	66	61%
Texts (including WhatsApp messages, etc)	13	34%	15	42%	12	35%	40	37%
Facebook/ other web-based social media platforms	3	8%	2	6%	2	6%	7	6%

For peer review only

Product/ service	Nairobi (N=38)		Mombasa (N=36)		Kisumu (N=34)		Total (N=108)	
	N or median	% or IQR	N or median	% or IQR	N or median	% or IQR	N or median	% or IQR

IQR: interquartile range
** Indicates significant difference between counties ($p<0.05$), based on chi square test or analysis of variance for means*
[1] A significant proportion of these sales may not have been related to COVD-19 prevention/ treatment
[2] Traditional/ alternative/ natural remedies included echinacea, chapa shoka, kaluma inhaler, menthol plus balm, and home remedies (lemon, papaya steaming)
[3] Antibiotics included azithromycin (n=55), cephalosporin (n=7), amoxicillin-containing (n=6), other macrolide (n=2); 3 pharmacies did not specify
[4] Other medicines included zinc+ vitamin D (n=4), vitamin B complex (n=1), salbutamol (n=1), and scotts emulsion (n=1); 2 pharmacies did not specify

Interview data suggested that many of these products and services were newly introduced following the outbreak, including products like vitamin C and surgical masks that one would have expected to find routinely in a pharmacy.

Demand for COVID-19 testing and provider views towards pharmacy-based testing

Fifty-nine (55% [95% CI 45-65%]) of 108 pharmacies reported ever receiving a client asking for COVID-19 testing, with a median [interquartile range] number of requests in the last week of 2 [1-4]. At the time of the survey, none of the pharmacies was providing COVID-19 testing; and among the 59 pharmacies experiencing demand for testing, alternatives given to clients included referral to other testing sites (94%) and body temperature measurement (15%). Referral options included: public health facilities (92%), private health facilities (27%), and private labs (13%).

Interviews revealed that test requests were mostly by clients with symptoms, those who suspected they had been exposed, and those who needed certification for travel or employment purposes (eg, drivers and hotel staff).

When asked if they thought COVID-19 testing should be provided in pharmacies, 60% [95% CI 50-70%] said “yes”. Among the 64 that were supportive of pharmacy-based testing, main reasons for support were: pharmacies are easily accessible (36%), clients perceive pharmacies as more confidential (23%), clients shy away from health facilities (19%), and demand exists (9%).

In interviews, the confidentiality and convenience of the pharmacy setting was reiterated, with some interviewees suggesting home-based self-testing as an option:

> *“If it [testing] is available in pharmacies, somebody can just take the kit, you can just go home, you test yourself. If you find you are positive, you can just self-quarantine, without raising an alarm. So that when the quarantine period is over, you don't get that kind of stigma, rejection.”*
>
> - Female pharmacy in charge, Mombasa, 201

For peer review only

Among the 42 that were not supportive of pharmacy-based testing, main reasons for opposing were: might be misused (29%), no private space in the pharmacy (24%), risk of infection (15%), protective gear is expensive or not available (12%), there is no demand for the service (10%), and staff are not adequately trained (10%). From the interviews, hesitancy to support pharmacy-based testing was mainly related to lack of capacity (*"I still need a thorough training…"*) and fear of infection.

Handling of simulated clients seeking covid-19-related services

When a simulated client called a pharmacy to ask if they have medicines that the client can use to protect themselves from getting COVID-19, 76 (74%) of 103 pharmacies recommended one or more medications: 27% recommended at least one conventional therapy and 71% at least one alternative therapy or nutritional supplement (**Table 3**). The main recommendations given to a client suspecting COVID-19 infection were to: get tested, visit the nearest health facility, and self-quarantine at home.

Table 3. Handling of simulated clients seeking COVID-19-related services at private retail pharmacies in Kenya.

**Call outcome**	**Nairobi (N=38)**		**Mombasa (N=36)**		**Kisumu (N=34)**		**Total (N=103)**	
	**n**	**%**	**n**	**%**	**n**	**%**	**n**	**%**
**Provider response to "*Do you have medicines that I can use to protect myself from getting COVID-19?*"**								
**Asked the client why he/she was asking for the medicines**	**14**	**38%***	**4**	**14%**	**2**	**6%**	**20**	**20%**
**Recommended conventional therapies**	**11**	**29%**	**6**	**19%**	**11**	**32%**	**28**	**27%**
Antibiotics	11	29%	6	19%	10	29%	27	26%
Azithromycin	9	24%	6	19%	9	26%	24	23%
Other antibiotics[1]	2	5%	1	3%	4	12%	7	7%
Painkillers	1	3%	0	0%	3	9%	4	4%
Antihistamines	0	0%	1	3%	2	6%	3	3%
**Recommended alternative therapies**	**29**	**76%**	**22**	**71%**	**22**	**65%**	**73**	**71%**
Vitamin C	28	74%	20	65%	19	56%	67	65%
Zinc	25	66%*	10	32%	7	21%	42	41%
Home remedies[2]	6	16%	4	13%	6	18%	16	16%
Vitamin B/ D	4	11%	4	13%	2	6%	10	10%
Multivitamins	2	5%	0	0%	3	9%	5	5%
Other remedies[3]	1	3%	0	0%	1	3%	2	2%
**Gave options on how to get the recommended medicines**	**37**	**97%**	**28**	**90%**	**34**	**100%**	**99**	**96%**
Pick from the pharmacy	21	55%	7	23%*	18	53%	46	45%
Home delivery	19	50%*	9	29%	4	12%	32	31%
Send someone to pick	5	13%	8	26%	4	12%	17	17%
**Provider response to "*What symptoms should I look out for if I suspect COVID-19 infection?*"**								

For peer review only - http://bmjopen.bmj.com/site/about/guidelines.xhtml

Call outcome	Nairobi (N=38)		Mombasa (N=36)		Kisumu (N=34)		Total (N=103)	
**Mentioned any symptoms**	**34**	**89%**	**29**	**94%**	**30**	**88%**	**93**	**90%**
Fever	23	61%	20	65%	21	62%	64	62%
Cough	13	34%	15	48%	16	47%	44	43%
Flu-like symptoms	15	39%	15	48%	12	35%	42	41%
Breathing difficulty	13	34%	12	39%	17	50%	42	41%
Loss/ change of smell or taste	16	42%	13	42%	11	32%	40	39%
Sore throat	15	39%*	8	26%	4	12%	27	26%
Chest pain or congestion	7	18%	4	13%	12	35%*	23	22%
Headache	6	16%	4	13%	8	24%	18	17%
Fatigue, tiredness, malaise	2	5%	8	26%*	4	12%	14	14%
Reduced appetite	2	5%	2	6%	7	21%	11	11%
Body aches/ pains	0	0%	3	10%	2	6%	5	5%
Gut symptoms, eg, stomach pain, diarrhea, etc	2	5%	0	0%	2	6%	4	4%
Dizziness	0	0%	2	6%	0	0%	2	2%
Weight loss	0	0%	0	0%	1	3%	1	1%
**Provider response to "*What should I do if I suspect I have COVID-19 infection?*"**								
**Immediate actions recommended**								
Get tested for COVID-19	25	66%	18	58%	16	47%	59	57%
Visit nearest health facility/ go see a doctor	18	47%	13	42%	24	71%*	55	53%
Self-quarantine/ isolate at home	3	8%	9	29%*	4	12%	16	16%
Take medications[4]	3	8%	2	6%	3	9%	8	8%
Check body temperature	2	5%	1	3%	2	6%	5	5%
Check oxygen levels	0	0%	1	3%	1	3%	2	2%
Check information online	0	0%	1	3%	1	3%	2	2%
Call 719[5]	2	5%	0	0%	0	0%	2	2%
**Supplementary actions recommended**								
Wear a mask	23	61%	16	52%	20	59%	59	57%
Maintain physical distance	21	55%	15	48%	18	53%	54	52%
Observe hand hygiene	7	18%	5	16%	7	21%	19	18%
Eat fruits	5	13%	5	16%	5	15%	15	15%
Eat well[6]	2	5%	3	10%	1	3%	6	6%
Take lots of fluids	1	3%	1	3%	3	9%	5	5%
Live well[7]	0	0%	0	0%	4	12%	4	4%
Other advice[8]	3	8%	1	3%	2	6%	6	6%
**Caller assessment of the interaction**								
**How friendly (approachable) was the person who served you?**								
1 - Very unfriendly	1	3%	0	0%	0	0%	1	1%
2 - Unfriendly	4	11%	3	10%	4	12%	11	11%
3 - Friendly	20	53%	22	71%	22	65%	64	62%
4 - Very friendly	13	34%	6	19%	8	24%	27	26%

For peer review only - http://bmjopen.bmj.com/site/about/guidelines.xhtml

Call outcome	Nairobi (N=38)		Mombasa (N=36)		Kisumu (N=34)		Total (N=103)	
**How enthusiastic was the provider about providing the service?**								
1 - Very unenthusiastic	1	3%	0	0%	0	0%	1	1%
2 - Unenthusiastic	4	11%	4	13%	7	21%	15	15%
3 - Enthusiastic	22	58%	22	71%	21	62%	65	63%
4 - Very enthusiastic	11	29%	5	16%	6	18%	22	21%
**Did the provider say or do anything that you found particularly helpful or nice?[9]**	**19**	**50%**	**9**	**29%**	**14**	**41%**	**42**	**41%**
**Did the provider say or do anything that you found particularly NOT helpful or NOT nice?[10]**	**4**	**11%**	**2**	**6%**	**7**	**21%**	**13**	**13%**

** Indicates significant difference between counties ($p<0.05$), based on chi square test*

[1] Other antibiotics included Augmentin, Amoxil, ceftriaxone, etc

[2] Home remedies included ginger/ lemon/ hot water drink, hot soup/ water, garlic, steam, frequent hot baths

[3] Other alternative therapies included ginsomin and omega-3

[4] Medications for treatment included: azithromycin (3%), other antibiotics (4%), painkillers (2%), steroids (1%), home remedies (1%)

[5] 719 is the government hotline for COVID-19 reporting and information-seeking

[6] Eat well: eat healthy, eat vegetables, etc

[7] Live well: exercise, enough rest, fresh air, don't panic, avoid stress, etc

[8] Other advice: don't touch face, avoid handshakes, do not take any medicines, don't reuse mask, etc

[9] Things that were deemed particularly helpful or nice related mainly to: reassurance that infection risk is low if prevention measures are followed; high chance of recovery in case of infection; detailed information on what to do or where to seek further help; and follow-up calls in cases where the provider was busy at the time of the initial call.

[10] Things that were deemed not helpful or not nice related mainly to: calls being hanged up; inattentiveness or being in a hurry to finish the call; and dismissiveness (eg, "don't you watch news? The symptoms are public knowledge!" and "we don't have those medicines you are asking for, jaribu kwingine [try elsewhere])

Infection prevention during service provision

The main infection prevention measures during service provision were hand hygiene, face masks, disinfection of services and physical distancing (**Table 4**). Eighty-five per cent of respondents rated the prevention measures instituted at their pharmacy as either adequate (72%) or very adequate (13%). Only one pharmacy reported that a staff member had ever tested positive for COVID-19, though no pharmacy reported regular testing of staff.

Table 4. COVID-19 infection prevention during service provision by private retail pharmacies in Kenya.

Infection prevention measures	Nairobi (N=38)		Mombasa (N=36)		Kisumu (N=34)		Total (N=108)	
	n	%	n	%	n	%	n	%
**Among staff**								
Physical distancing[1]	25	66%	26	72%	27	79%	78	72%
Temperature monitoring	13	34%	14	39%	13	38%	40	37%
Hand hygiene	35	92%	33	92%	32	94%	100	93%

For peer review only - http://bmjopen.bmj.com/site/about/guidelines.xhtml

Infection prevention measures	Nairobi (N=38)		Mombasa (N=36)		Kisumu (N=34)		Total (N=108)	
Disinfection of surfaces	29	76%	30	83%	27	79%	86	80%
Face masks[2]	34	89%	30	83%	30	88%	94	87%
Goggles	0	0%	1	3%	1	3%	2	2%
Ingestion of immune boosters	1	3%	0	0%	0	0%	1	1%
**Among clients**								
Physical distancing[1]	33	87%	31	86%	30	88%	94	87%
Temperature monitoring	13	34%	9	25%	11	32%	33	31%
Hand hygiene[3]	36	95%	33	92%	30	88%	99	92%
Face masks	27	71%	32	89%	27	79%	86	80%
Non-touch payment methods[4]	14	37%	16	44%	10	29%	40	37%
Information materials and messages[5]	12	32%	13	36%	11	32%	36	33%

[1] Physical distancing was achieved through various means including: reducing number of staff and working in shifts, ensuring a distance of 1.5 -3 meters between staff and clients through the use of posters and floor/seat markings, serving one client at a time or other crowd control measures, ribbons/ ropes/ glass/ metal-grill/ wooden barriers between client and provider, and home delivery of products. One pharmacy noted that "patients with cough and related symptoms are always first referred to the health center for covid19 testing before being served further".
[2] Majority of pharmacies (95%) were using surgical/medical masks; only 6% reported use of cloth masks.
[3] Hand hygiene measures among clients included: handwashing, hand sanitizer, and "no hand-shakes" policy
[4] Non-touch payment methods included: mobile money, and credit/ debit cards; one pharmacy reported disinfection of cash.
[4] Messages were delivered through: posters (21%), leaflets (5%), television screens (2%), and audio announcements (1%)

Information sources and training

Sources of general COVID-19 information included: continuing professional development sessions (37% of respondents), ministry of health (MoH) communications (23%), social media and internet (19%), television and radio (12%), Kenya Pharmaceutical Association (KPA)/ Pharmaceutical Society of Kenya (PSK) communications (5%), scientific journals (2%) and colleagues (2%). For specific questions about COVID-19, providers consulted the pharmacy-in-charge (48%), nearby doctor (37%), MoH officials (32%), the internet (5%), and medical representatives (2%).

Interviewees felt that the official announcement of the first case played was a major source of awareness, though some reported having learnt about the outbreak through radio, television, and social media prior to the first reported case. However, they decried the rapidly changing and sometimes contradictory information, for example *"on whether to use or not to use masks"*.

Fifty-two (48%) pharmacies had at least one staff member trained on COVID-19, with a median [range] of 1 [1-8] providers trained. This did not vary by county or by membership in professional associations. Modalities of training included: on-site in the pharmacy (67% of respondents), virtual (54%), and off-site (13%). Virtual trainings were more prevalent in Kisumu compared to Nairobi (76% vs 35%, p=0.04). Training sessions were facilitated by MoH (73%), KPA (51%), PSK (19%), AMREF (9%), and Kenyatta National Hospital (5%). While some interview participants felt they had received "a lot'' of training, others felt the trainings were not very adequate, particularly

For peer review only

For peer review only - http://bmjopen.bmj.com/site/about/guidelines.xhtml

with regard to inclusion of updated information in follow-up sessions and the fact that most trainings included only the pharmacy in-charge and not the entire staff.

External support and linkages

Interviewees expressed appreciation for guidance from the ministry of health, including the pharmacy and poisons board, who *"did some inspection and brought some banners [information materials] on how I can protect myself and the general public."* The government-led community awareness-creation activities were also hailed as an important complement to pharmacy efforts in educating the public.

However, examples of direct linkages and material support were rare. One interviewee in Nairobi reported that community health workers from a nearby health centre regularly visited the pharmacy to collect information on number of suspect cases seen. Another interviewee in Kisumu described an instance where an ambulance was sent promptly on reporting a patient in critical condition.

More commonly, a feeling of isolation and neglect prevailed, with pharmacies reporting that they were not receiving much support from the government or other external parties with regard to COVID-19, unlike what happens with other public health programs:

> *"So far being a private facility, we are not receiving any financial support from the organizations in COVID related issues. We don't receive that apart from PS Kenya [NGO supporting TB and HIV programs]."*
>
> - Male pharmaceutical technologist, Kisumu, 301

Confidence, motivation, and coping mechanisms

Majority of questionnaire respondents reported feeling confident (68%) or very confident (19%) about providing COVID-19-related services. Over half felt prepared (51%) or very prepared (7%) about handling a client who presents to the pharmacy with COVID-19 symptoms.

Motivation for supporting delivery of COVID-19 services included: "to help people protect themselves or prevent transmission (75%), "It's part of my job/ I am required to do it" (14%), encouragement and support from professional associations (7%), "to boost the profile of the pharmacy" (2%), and "to make money" (1%). When asked how much they felt their personal role in the COVID-19 response was valued by the community, the majority stated highly valued (68%) or very highly valued (11%)

In addition to the operational adjustments described above, interviewees described other coping mechanisms such putting up a shade outside to prevent crowding inside the pharmacy and improvisation of hand sanitizers when not available from suppliers. Some interviewees also reported going the extra mile sometimes, eg, by providing free masks to clients.

For peer review only

Provider suggestions and recommendations

Seventy-six (70%) questionnaire respondents highlighted inputs that they felt pharmacies can uniquely contribute to the national response, including: provision of information and counselling to clients (64%), distribution of preventive materials (21%), triage and referral of suspect cases (17%), surveillance of infection rates and community adherence to preventive measures (14%), provision of preventive and curative therapies (12%), and maintenance of long-term therapy (3%). Pharmacies' location within communities was cited as a strength when it comes to surveillance of client behaviour and community awareness creation.

To facilitate these inputs, pharmacies suggested additional support that they would require from the government and other external parties, including: training (34% of respondents), free or subsidized preventive materials such as masks, sanitizers, and thermo guns for own use and distribution to clients (34%), supply management such as price and quality controls (15%), clear guidelines and referral systems (9%), community awareness creation (7%), information materials (6%), financial support such as loan repayment holidays and medical insurance (6%), and testing services (5%).

These suggestions were echoed in interviews. Post-marketing surveillance to ensure quality of products in the market and a streamlined process to capture and transmit information back to the government were pinpointed as key gaps in the pandemic response. Overall, there were earnest calls for more deliberate engagement, oversight, and support supervision by the government and other implementing partners:

> *"If the government could put us, the pharmacists and pharm techs in the you know, treat us like people who are fighting this virus and facilitate this thing it would be a major shift, it would be a major shift."*
>
> \- Male pharmacy in charge, Mombasa, 205

Discussion

In this study among private retail pharmacies (community pharmacies) in Kenya, we describe the impact of the COVID-19 pandemic on operations, COVID-19-related products and services being provided, infection prevention during service provision, and provider suggestions and recommendations on how to improve pandemic preparedness and response.

The initial weeks after the pandemic reached Kenya were characterized by fear and panic among providers and a surge in client flow. Broadly speaking, the experiences documented in our study can be captured with the apt words of Austin et al in their paper describing the experiences of community pharmacists in Ontario, Canada, viz: "the scale and magnitude of the COVID-19 pandemic defied imagination or pre-planning, yet health care professionals like pharmacists were required to continue to provide services and care to patients – and in many cases, expand their repertoire of clinical skills to assume new and even more challenging responsibilities" [15].

For peer review only - http://bmjopen.bmj.com/site/about/guidelines.xhtml

The initial surge in demand waned rapidly, with client flow dipping below pre-pandemic levels within a few weeks and remaining depressed until the time of data collection, seven months later. This was surprising as we had hypothesized, before the study, that pharmacies would experience an overall increase in demand as patients redirected their care-seeking from health facilities for fear of exposure to infection and quarantine. Participants attributed the decrease in demand mainly to the movement restrictions instituted by government. However, they also suggested – ironically – that clients, especially those with flu-like symptoms, may have avoided pharmacies for fear of being referred to public facilities and subsequently being quarantined.

Our findings clearly show that community pharmacies in Kenya were undertaking potentially impactful activities to support the response to the COVID-19 pandemic. Despite a feeling of disconnection from the government-led national response, providers expressed great readiness and motivation for provision of COVID-19-related services, similar to providers in Qatar [30], the USA [31], and other settings around the world [32]. The services pharmacies in our study were providing are generally similar to those suggested by experts in the field [22, 33-35], as well as those being provided in other low-to-middle income countries [36-39] and in high-income settings [30, 40, 41].

However, management of drug shortages and prevention of stockpiling (hoarding) by clients [22, 30, 33] were reported only as challenges and not as roles that Kenyan pharmacies were playing or could play. Additionally, while 60% of questionnaire respondents said they were receiving queries and providing information and counseling over the phone, other remote services such as online prescribing and home delivery of medications [22, 36] were not being widely used or mentioned as potential additional roles. Participants in our study, being frontline workers, may have deemed the carrying out of these innovations as being beyond the sphere of their influence. Similarly, COVID-19 vaccine administration was not mentioned as a potential role, understandably perhaps because a vaccine was not available at the time. But pharmacies in Kenya are actively involved in provision of other vaccines [42, 43], hence it is reasonable to hypothesize that COVID-19 vaccines could be delivered through private pharmacies in the future. Interviews with policymakers and captains of industry may be required to probe these topics further.

The finding that no pharmacy was providing COVID-19 testing was unsurprising since no approved point-of-care (POC) tests were available at the time of the study. The more interesting finding is that there was significant demand and support for pharmacy-based testing, similar to what studies in the Middle East [44] and the USA [31] have found. In this context and given the fact that at least three rapid POC tests have since been approved in Kenya [45], there is an urgent need to start developing policies to guide implementation. This will obviate the emergence of an uncoordinated and potentially dangerous service, similar to the HIV testing one that we observed in 2016 before policy guidelines were issued [46, 47].

For peer review only - http://bmjopen.bmj.com/site/about/guidelines.xhtml

For treatment of COVID-19 symptoms, most pharmacies recommended alternative therapies and nutritional supplements such as vitamin C and zinc, and only about a third suggested conventional therapies with no proven efficacy such as antibiotics [13, 14]. This finding is similar to that of a small study in Nairobi in May 2020 which found that despite increased requests for antimalarials and antibiotics, pharmacists recommended alternative therapies [48]. A similar finding was also obtained in a multinational survey of Asian pharmacists [35]. While alternative therapies and nutritional supplements are themselves not evidence-based (as no rigorous trials have proven their efficacy against SARS-CoV-2), they have a better safety profile and their useful effects are more biologically plausible since they have been used for ages against the flu virus, itself a coronavirus. These findings are therefore encouraging. However, among Egyptian pharmacies, the antibiotic azithromycin was given to about 40% of presumptive COVID-19 patients with mild to moderate symptoms, albeit based on physician recommendation [49]. Further research in different jurisdictions may be needed to describe and contrast the use of antibiotics and other unproven COVID-19 therapies, in order to inform the promotion of rational drug use and the prevention of antimicrobial resistance.

Our study identified other important gaps in community pharmacies' preparedness and response to the pandemic. Notably, pharmacies did not seem to have any well-defined operational linkages to other units of the healthcare system, including for example, the emergency operations center at the ministry of health. Besides, while only about half of pharmacies had at least one staff member trained on COVID-19, some of those trained felt they had received a lot of training, suggesting a "network effect" where some providers were receiving many trainings while others received none.

Specific programs will be required to address these gaps and enhance pandemic preparedness. Templates for public-private cooperation in pandemic responses do exist from other settings. Ung *et al*, for example, report how in one administrative region on the south-east coast of China, pre-existing public-private partnerships between the local government and community pharmacies ensured rapid provision and prevented hoarding of face masks through a government-led "Guaranteed Mask Supply for Macao Residents Scheme" [50]. A qualitative study in Canada, identified key features that predicted community pharmacists' resilience during the early period of the pandemic, including: use of and comfort with technology; early adoption of corporate and professional guidance; emphasis on task-focus rather than multi-tasking; and provision of personal protective equipment [15]. Roll-out of pharmacy-based COVID-19 programs should be accompanied with implementation studies to better uncover aspects of capacity, preparedness, and resilience.

Our study had a number of strengths. First, we took advantage of information collected in a recent study to speed up recruitment and data collection. Second, we used multiple quantitative and qualitative methods to enhance the comprehensiveness and reliability of the findings. Of

For peer review only - http://bmjopen.bmj.com/site/about/guidelines.xhtml

note, we used simulated clients to objectively assess provider practices, an approach that is gaining acceptance as a gold standard for the measurement of clinical practice quality around the world [51]. One weakness of our study was the low response rate (55%), considering that we targeted pharmacies that had participated in a similar study less than a year previously. Compared to non-participating pharmacies, participating pharmacies had features suggestive of higher operational capacity and interest in public health interventions. While this limits generalizability of our findings, it can also be looked at in positive light. We propose that pharmacies with these characteristics could be identified for inclusion in a "practice and learning network" that could provide a platform for testing implementation strategies for various public health interventions [52-54]. Scale up of such interventions would then target similar pharmacies and include strategies for enhancing operational capacity and interest in public health among the other pharmacies.

In conclusion, we found that private retail pharmacies in Kenya were actively contributing to the COVID-19 response despite an apparent disconnection from the national response program. More deliberate engagement, support and linkages are required. Notably, there is an urgent need to develop policy guidelines for pharmacy-based COVID-19 testing, a service that is clearly needed and which could greatly increase test coverage. Roll-out of this and other pharmacy-based COVID-19 programs should be accompanied with implementation research in order to inform current and future pandemic responses.

Acknowledgments

We acknowledge the contributions of all participating pharmacy service providers. We thank James Wafula for supporting database development and data management; and Langat Justus, Lucy Omumbo, Milkah Auka, and Rodgers Ayoma for supporting the simulated client survey.

Author Contributions

Conceived the study: PM EB. Developed protocol: PM DM SM EB. Performed the experiments: PM AM; Analysed the data: PM AM JN. Wrote the manuscript: PM AM DM JN SM EB. Approved the final manuscript: PM AM DM JN SM EB.

The authors declare not conflicts of interest.

Funding statement

This work was supported by Wellcome Trust [#211353/Z/18/Z]. For the purpose of open access, the author has applied a CC-BY public copyright licence to any author accepted manuscript version arising from this submission. The KWTRP at the Centre for Geographical Medicine Research-Nairobi is supported by core funding from the Wellcome Trust [#077092]. This report was published with the permission of Director KEMRI CGMRC.

Patient and Public Involvement

For peer review only - http://bmjopen.bmj.com/site/about/guidelines.xhtml

An early version of the study protocol received inputs from the Pharmaceutical Society of Kenya (PSK) COVID-19 response taskforce composed of pharmacy sector stakeholders. Preliminary findings were shared with the pharmacy sector at the 2021 PSK Annual Conference and a policy brief has been distributed to stakeholders in the ministry of health, pharmacy sector and other implementing partners. Findings were also discussed in a local Radio show in February 2022.

Data availability statement

Underlying data can be accessed in the Harvard Dataverse using the following link: https://doi.org/10.7910/DVN/XTLUFP

References

1. *Zhu N, Zhang D, Wang W, Li X, Yang B, Song J, et al.* A novel coronavirus from patients with pneumonia in China, 2019. *New England Journal of Medicine* 2020.
2. *Anand U, Cabreros C, Mal J, Ballesteros Jr F, Sillanpää M, Tripathi V, et al.* Novel coronavirus disease 2019 (COVID-19) pandemic: From transmission to control with an interdisciplinary vision. *Environmental research* 2021:111126.
3. Coronavirus cases and deaths. https://www.worldometers.info/coronavirus/ Updated, Date Accessed: 29/09.
4. *Lai S, Ruktanonchai NW, Zhou L, Prosper O, Luo W, Floyd JR, et al.* Effect of non-pharmaceutical interventions for containing the COVID-19 outbreak: an observational and modelling study. *medRxiv* 2020.
5. *Cowling BJ, Ali ST, Ng TW, Tsang TK, Li JC, Fong MW, et al.* Impact assessment of non-pharmaceutical interventions against coronavirus disease 2019 and influenza in Hong Kong: an observational study. *The Lancet Public Health* 2020.
6. *Fauci AS*. The story behind COVID-19 vaccines: American Association for the Advancement of Science, 2021.
7. *Kashte S, Gulbake A, El-Amin III SF, Gupta A*. COVID-19 vaccines: rapid development, implications, challenges and future prospects. *Human cell* 2021:1-23.
8. *US National Institutes of Health*. Care of Critically Ill Adult Patients With COVID-19. 2020. https://www.covid19treatmentguidelines.nih.gov/critical-care/
9. *US National Institutes of Health*. Outpatient Management of Acute COVID-19. 2021. https://www.covid19treatmentguidelines.nih.gov/outpatient-management/
10. *Jin Y, Yang H, Ji W, Wu W, Chen S, Zhang W, et al.* Virology, epidemiology, pathogenesis, and control of COVID-19. *Viruses* 2020;12(4):372.
11. *Siemieniuk RA, Bartoszko JJ, Ge L, Zeraatkar D, Izcovich A, Kum E, et al.* Drug treatments for covid-19: living systematic review and network meta-analysis. *BMJ* 2020;370:m2980.
12. *American Society of Hospital Pharmacists*. Assessment of Evidence for COVID-19-Related Treatments. 2021. https://www.ashp.org/-/media/assets/pharmacy-practice/resource-centers/Coronavirus/docs/ASHP-COVID-19-Evidence-Table.ashx?la=en&hash=FD5D4C4AEDAC4B725B85D3375A88216E526C39EC
13. *World Health Organization*. Clinical management of COVID-19. 2020.
14. *US National Institutes of Health*. Prevention and Prophylaxis of SARS-CoV-2 Infection. 2021. https://www.covid19treatmentguidelines.nih.gov/overview/prevention-of-sars-cov-2/

For peer review only

15. *Austin Z, Gregory P*. Resilience in the time of pandemic: the experience of community pharmacists during COVID-19. *Research in Social and Administrative Pharmacy* 2021;17(1):1867-75.
16. *Abuya TO, Mutemi W, Karisa B, Ochola SA, Fegan G, Marsh V*. Use of over-the-counter malaria medicines in children and adults in three districts in Kenya: implications for private medicine retailer interventions. *Malar J* 2007;6:57.
17. *Bigogo G, Audi A, Aura B, Aol G, Breiman RF, Feikin DR*. Health-seeking patterns among participants of population-based morbidity surveillance in rural western Kenya: implications for calculating disease rates. *Int J Infect Dis* 2010;14(11):e967-73.
18. *Tran BX, Vu GT, Le HT, Pham HQ, Phan HT, Latkin CA, et al.* Understanding health seeking behaviors to inform COVID-19 surveillance and detection in resource-scarce settings. *Journal of Global Health* 2020;10(2).
19. *Fonck K, Mwai C, Rakwar J, Kirui P, Ndinya-Achola JO, Temmerman M*. Healthcare-seeking behavior and sexual behavior of patients with sexually transmitted diseases in Nairobi, Kenya. *Sex Transm Dis* 2001;28(7):367-71.
20. *Onwujekwe O, Hanson K, Uzochukwu B*. Do poor people use poor quality providers? Evidence from the treatment of presumptive malaria in Nigeria. *Trop Med Int Health* 2011;16(9):1087-98.
21. *International Pharmaceutical Federation*. Guidelines for Pharmacists and the Pharmacy Workforce to manage COVID-19. 2020. https://www.fip.org/publications?publicationCategory=97&publicationYear=2020&publicationKeyword=
22. *Aruru M, Truong H-A, Clark S*. Pharmacy Emergency Preparedness and Response (PEPR): a proposed framework for expanding pharmacy professionals' roles and contributions to emergency preparedness and response during the COVID-19 pandemic and beyond. *Research in Social and Administrative Pharmacy* 2021;17(1):1967-77.
23. *Shaw, N*. Kenya: protocols for the containment of the coronavirus (COVID-19) announced. 2020. https://www.ensafrica.com/news/detail/2373/kenya-announces-protocols-for-the-containment
24. *Worldometer*. Kenya Coronavirus Cases. 2021. https://www.worldometers.info/coronavirus/country/kenya/
25. *Wangari EN, Gichuki P, Abuor AA, Wambui J, Okeyo SO, Oyatsi HT, et al.* Kenya's response to the COVID-19 pandemic: a balance between minimising morbidity and adverse economic impact. *AAS Open Research* 2021;4.
26. *Barasa E, Kazungu J, Orangi S, Kabia E, Ogero M, Kasera K*. Assessing the Indirect Health Effects of the COVID-19 Pandemic in Kenya. *CGD Work. Pap* 2021;570.
27. *Global Alliance for Vaccines and Immunization*. Kenya completes its first round of COVID-19 vaccinations. 2021. https://www.gavi.org/vaccineswork/kenya-completes-its-first-round-covid-19-vaccinations
28. *Pharmacy and Poisons Board*. Online Services Portal: License Status. 2019. https://practice.pharmacyboardkenya.org/LicenseStatus
29. *Statista*. Cumulative number of confirmed coronavirus (COVID-19) cases in Kenya by county. 2021. https://www.statista.com/statistics/1136519/cumulative-coronavirus-cases-in-kenya-by-county/
30. *ElGeed H, Owusu Y, Abdulrhim S, Awaisu A, Kattezhathu VS, Abdulrouf PV, et al.* Evidence of community pharmacists' response preparedness during COVID-19 public health crisis: A cross-sectional study. *The Journal of Infection in Developing Countries* 2021;15(01):40-50.

For peer review only - http://bmjopen.bmj.com/site/about/guidelines.xhtml

31. *Nguyen E, Owens CT, Daniels T, Boyle J, Robinson RF*. Pharmacists' Willingness to Provide Coronavirus Disease (COVID-19) Services and the Needs to Support COVID-19 Testing, Management, and Prevention. *Journal of community health* 2020:1-6.
32. *Jordan D, Guiu-Segura JM, Sousa-Pinto G, Wang L-N*. How COVID-19 has impacted the role of pharmacists around the world. *Farmacia Hospitalaria: Organo Oficial de Expresion Cientifica de la Sociedad Espanola de Farmacia Hospitalaria* 2021;45(2):89-95.
33. *Liu S, Luo P, Tang M, Hu Q, Polidoro JP, Sun S, et al.* Providing pharmacy services during the coronavirus pandemic. *International Journal of Clinical Pharmacy* 2020;42(2):299-304.
34. *Bukhari N, Rasheed H, Nayyer B, Babar Z-U-D*. Pharmacists at the frontline beating the COVID-19 pandemic: Springer, 2020.
35. *Godman B, Haque M, Islam S, Iqbal S, Urmi UL, Kamal ZM, et al.* Rapid assessment of price instability and paucity of medicines and protection for COVID-19 across Asia: findings and public health implications for the future. *Frontiers in public health* 2020;8.
36. *Bahlol M, Dewey RS*. Pandemic preparedness of community pharmacies for COVID-19. *Research in Social & Administrative Pharmacy* 2021;17(1):1888.
37. *Meghana A, Aparna Y, Chandra SM, Sanjeev S*. Emergency preparedness and response (EP&R) by pharmacy professionals in India: lessons from the COVID-19 pandemic and the way forward. *Research in Social and Administrative Pharmacy* 2021;17(1):2018-22.
38. *Kasahun GG, Kahsay GM, Asayehegn AT, Demoz GT, Desta DM, Gebretekle GB*. Pharmacy preparedness and response for the prevention and control of coronavirus disease (COVID-19) in Aksum, Ethiopia; a qualitative exploration. *BMC health services research* 2020;20(1):1-6.
39. *Hoti K, Jakupi A, Hetemi D, Raka D, Hughes J, Desselle S*. Provision of community pharmacy services during COVID-19 pandemic: a cross sectional study of community pharmacists' experiences with preventative measures and sources of information. *International journal of clinical pharmacy* 2020;42(4):1197-206.
40. *Zaidi STR, Hasan SS*. Personal protective practices and pharmacy services delivery by community pharmacists during COVID-19 pandemic: Results from a national survey. *Research in Social and Administrative Pharmacy* 2021;17(1):1832-37.
41. *Giua C, Paoletti G, Minerba L, Malipiero G, Melone G, Heffler E, et al.* Community pharmacist's professional adaptation amid Covid-19 emergency: a national survey on Italian pharmacists. *International journal of clinical pharmacy* 2021:1-8.
42. *Goodlife Pharmacy*. Get your vaccinations today. 2021. https://www.goodlife.co.ke/get-your-vaccinations-today/
43. *Portal Pharmacy*. Flu vaccine. 2021. https://portalpharmacy.ke/flu-vaccine
44. *Mukattash TL, Jarab AS, Abu-Farha RK, Nusair M, Mukattash IL, Obaidat RM, et al.* Willingness and readiness to test for COVID-19; A qualitative exploration of community pharmacists. *International journal of clinical practice* 2020;74(12):e13620.
45. *Pharmacy and Poisons Board, Kenya Ministry of Health*. View Medical Devices. 2021. https://products.pharmacyboardkenya.org/ppb_admin/pages/system_reports_public.php
46. *Mugo PM, Micheni M, Shangala J, Hussein MH, Graham SM, Rinke de Wit TF, et al.* Uptake and Acceptability of Oral HIV Self-Testing among Community Pharmacy Clients in Kenya: A Feasibility Study. *PLoS One* 2017;12(1):e0170868.
47. *National AIDS and STI Control Programme (NASCOP)*. An operational manual for the delivery of HIV Self-Testing services in Kenya. 2017.
48. *Opanga SA, Rizvi N, Wamaitha A, Sefah IA, Godman B*. Availability of medicines in community pharmacy to manage patients with COVID-19 in Kenya; pilot study and implications. *Scholars Academic Journal of Pharmacy* 2021;10(3):36-43.

For peer review only - http://bmjopen.bmj.com/site/about/guidelines.xhtml

49. *Elsayed AA, Darwish SF, Zewail MB, Mohammed M, Saeed H, Rabea H*. Antibiotic misuse and compliance with infection control measures during COVID-19 pandemic in community pharmacies in Egypt. *International journal of clinical practice* 2021;75(6):e14081.
50. *Ung COL*. Community pharmacist in public health emergencies: Quick to action against the coronavirus 2019-nCoV outbreak. *Research in Social and Administrative Pharmacy* 2020;16(4):583-86.
51. *Daniels B, Dolinger A, Bedoya G, Rogo K, Goicoechea A, Coarasa J, et al.* Use of standardised patients to assess quality of healthcare in Nairobi, Kenya: a pilot, cross-sectional study with international comparisons. *BMJ global health* 2017;2(2):e000333.
52. *English M, Ayieko P, Nyamai R, Were F, Githanga D, Irimu G*. What do we think we are doing? How might a clinical information network be promoting implementation of recommended paediatric care practices in Kenyan hospitals? *Health research policy and systems* 2017;15(1):1-12.
53. *Gachau S, Ayieko P, Gathara D, Mwaniki P, Ogero M, Akech S, et al.* Does audit and feedback improve the adoption of recommended practices? Evidence from a longitudinal observational study of an emerging clinical network in Kenya. *BMJ global health* 2017;2(4):e000468.
54. *Tuti T, Bitok M, Malla L, Paton C, Muinga N, Gathara D, et al.* Improving documentation of clinical care within a clinical information network: an essential initial step in efforts to understand and improve care in Kenyan hospitals. *BMJ Global Health* 2016;1(1):e000028.

For peer review only

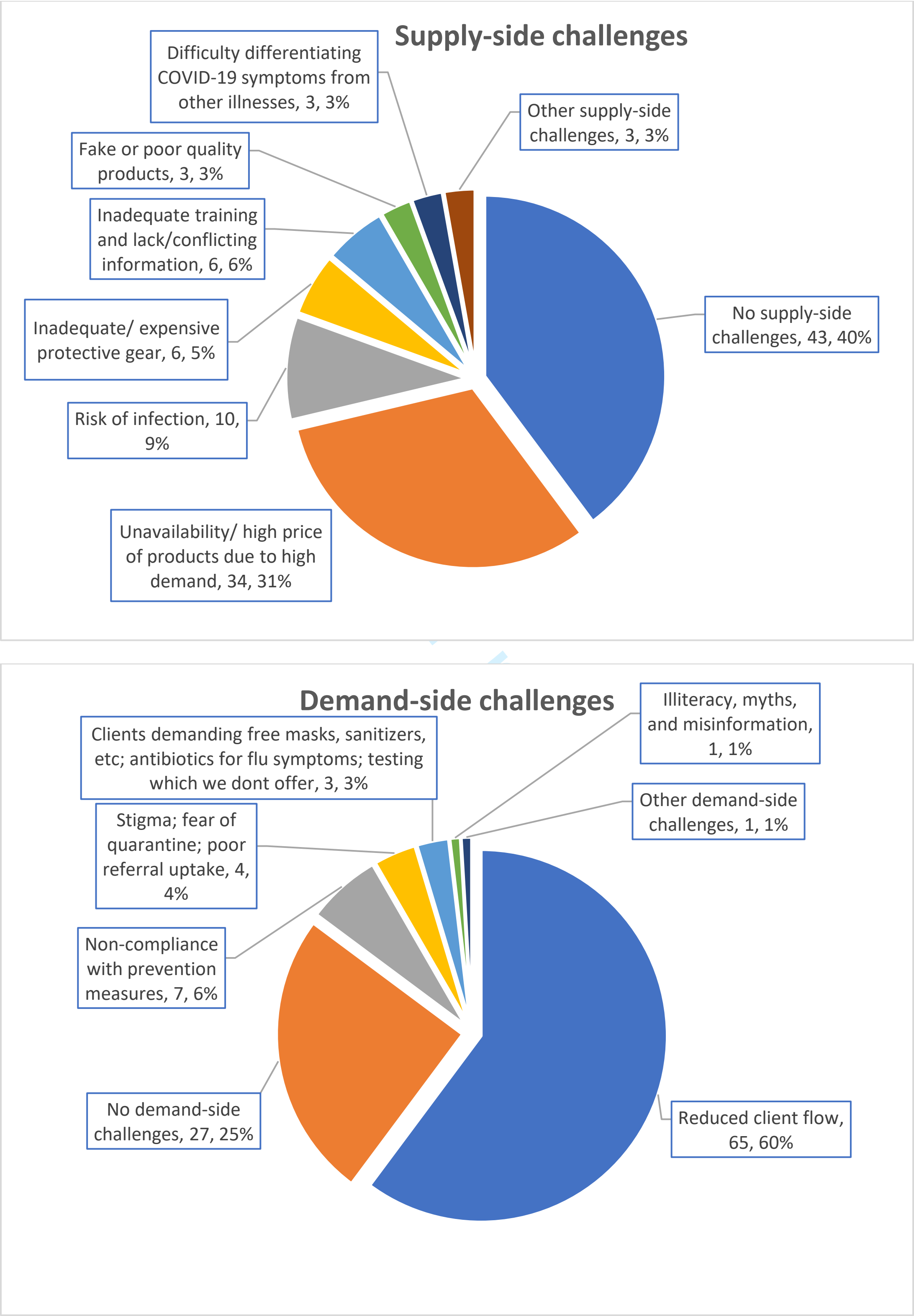

Figure 1. Challenges experienced by pharmacies during the COVID-19 pandemic in Kenya

Supplementary Table I: Characteristics of participating pharmacies and respondents

A. Characteristics of participating pharmacies

Characteristic	Nairobi (n=38)		Mombasa (n=36)		Kisumu (n=34)		Total (n=108) ^	
	n	%	n	%	n	%	n	%
**Setting**								
Urban commercial centre	23	61%	29	81%	12	35%	64	59%
Urban residential area	13	34%	4	11%	6	18%	23	21%
Urban informal settlement	2	5%	3	8%	2	6%	7	6%
Rural town/ shopping centre	0	0%	0	0%	14	41%*	14	13%
**Years of operation**								
Number of years in operation (median [range])	5	1-19	4	1-42	5	1-11	5	1-42
**Physical facilities**								
Total number of rooms (Median [range])	2	1-10	2	1-6	2	1-8	1	1-10
Consultation room	14	37%	10	28%	20	59%*	44	41%
On-site lab	5	13%	1	3%	6	18%	12	11%
**Information systems**								
Computerized stock system	29	81%*	19	53%	23	68%	71	67%
Individual medical records[1]	9	25%	2	6%	7	21%	18	17%
**Chain network membership[2]**								
Part of a chain network	9	24%*	3	8%	1	3%	13	12%
Number of branches nationwide (Median [range])	1	1-41	1	1-41	1	1-9	1	1-41
**Branding[3]**								
None	33	87%	29	81%	32	94%	94	87%
Green Cross	2	5%	2	6%	0	0%	4	4%
Pharmnet (blue cross)	0	0%	3	8%	1	3%	4	4%
Corporate	3	8%	2	6%	1	3%	6	6%
**Distance to the nearest clinic**								
Within the pharmacy	0	0%	0	0%	2	6%	2	2%
< 100 meters	17	45%	13	36%	9	26%	39	36%
< 1km	19	50%	16	44%	21	62%	56	52%
< 5km	2	5%	7	19%	2	6%	11	10%
**Distance to the nearest HIV testing site**								
< 100 meters	14	37%	12	33%	11	32%	37	34%
< 1km	18	47%	16	44%	21	62%	55	51%
< 5km	4	11%	7	19%	2	6%	13	12%
>5km	1	3%	0	0%	0	0%	1	1%
Not sure	1	3%	1	3%	0	0%	2	2%
**Service providers**								
Total number of service providers (Median [range])	3*	1-16	2	1-9	2	1-4	2	1-16
At least one pharmacy degree holder	8	21%	7	19%	3	9%	18	17%

For peer review only

Characteristic	Nairobi (n=38)		Mombasa (n=36)		Kisumu (n=34)		Total (n=108) ^	
	n	%	n	%	n	%	n	%
At least one pharmacy diploma holder	36	95%	35	97%	34	100%	105	97%
All staff belong in a professional association	24	63%	19	53%	18	53%	61	56%
**Management**								
Written job descriptions	21	55%	12	33%	14	41%	47	44%
Regular staff meetings[4]	21	55%	16	44%	19	56%	56	52%
**Opening hours**								
24 hours daily, 7 days a week	4	11%	1	3%	1	3%	6	6%
At least seven days a week	23	61%	28	78%	25	74%	76	70%
At least 6 days a week	38	100%	34	94%	34	100%	106	98%
**Work load**								
Walk-in clients per day (Median [range])	55	15-300	50	20-200	40	20-200	50	15-300
Online clients per day (Median [range])	1	0-20	0	0-20	0	0-10	0	0-20
**Health promotion services**								
Over-the-counter STI treatment	29	76%	29	81%	30	88%	88	81%
Weight management	12	32%	6	17%	10	29%	28	26%
Smoking cessation	4	11%	1	3%	1	3%	6	6%
Vaccination	0	0%	1	3%	3	9%	4	4%
**Screening services**								
Blood pressure measurement	26	68%	26	72%	25	74%	77	71%
Blood sugar testing	19	50%	25	69%	20	59%	64	59%
HIV self-testing	21	55%	22	61%	20	59%	63	58%
Malaria testing	9	24%	19	53%	27	79%*	55	51%
In-pharmacy pregnancy testing	9	24%	9	25%	18	53%*	36	33%
Other screening services[5]	1	3%	0	0%	5	15%*	6	6%

^ Of 195 target pharmacies that had participated in the previous PHP study, 108 (55%) participated in the current survey. Of 87 that did not participate: 55 (63%) failed to complete the questionnaire after three reminders, 15 (17%) reported that the initial respondent had left and no one else was willing to participate, 8 (9%) were unreachable, 3 (3%) reported that the management did not approve participation, 2 (1%) were undergoing transition, 1 (1%) was closed, and 3 (3%) had other reasons (no access to smartphone or computer, preferred face-to-face interview, sold the pharmacy).
** Indicates significant difference between counties (p<0.05), based on chi square test or analysis of variance for means*
[1] An individual medical record is a document detailing the medications an individual patient is on, indications and drug allergies; may also include other clinical information.
[2] Chain network was defined as 6 or more branches nationwide, derived from chain size distribution pattern; 79 (73%) pharmacies were single branch, 16 (15%) were part of a 2-5 branch network, and 13 (12%) were part of a chain network
[3] Green Cross is a professional network of pharmacies owned by pharmacists; Pharmnet is for pharmaceutical technologists
[4] Excludes 18 pharmacies that responded "not applicable" since they have only one staff member
[5] Other screening services included; typhoid testing (n=4) and tuberculosis screening (n=2)
STI: Sexually transmitted infection

For peer review only

B. Characteristics of respondents

Characteristic	Nairobi (N=38)		Mombasa (N=36)		Kisumu (N=34)		Total (N=108)	
	n	%	n	%	n	%	n	%
**Gender**								
Female	12	32%	13	36%	16	47%	41	38%
Male	26	68%	23	64%	18	53%	67	62%
**Age**								
20 – 29 years	12	32%	12	33%	14	41%	38	35%
30 – 39 years	19	50%	18	50%	16	47%	53	49%
40 – 44 years	5	13%	4	11%	2	6%	11	10%
45 years and over	2	5%	2	6%	2	6%	6	6%
**Designation (role)**								
Owner	9	24%	12	33%	12	35%	33	31%
In-charge/ Superintendent	11	29%	13	36%	8	24%	32	30%
Staff[1]	18	47%	11	31%	14	41%	43	40%
**Highest level of education[2]**								
Non-health qualifications	0	0%	2	6%	1	3%	3	3%
Health-related certificate/ diploma/ bachelor's degree	2	5%	0	0%	2	6%	4	4%
Pharmacy certificate	2	5%	0	0%	1	3%	3	3%
Pharmacy Diploma	29	76%	31	86%	28	82%	88	81%
Pharmacy degree	3	8%	3	8%	0	0%	6	6%
Health-related master's degree	2	5%	0	0%	2	6%	4	4%
**Experience (Median [Range])**								
Total number of years worked	7	1-25	5	2-41	6	2-20	6	1-41
Number of years worked in the current pharmacy	3	1-14	3	1-30	3	1-17	3	1-30

[1] Staff designations included: pharmaceutical technologist (n=35), pharmacist (n=5), pharmacy assistant (n=2), and nurse aid (n=1)
[2] Non-health qualifications included: business management (n=1), supplies management (n=1), and college (n=1); health-related certificate/ diploma/ bachelor's degree included: medicine (n=1), nursing (n=2), and environmental health (n=1)h; health-related master's degree included: pharmacy (n=1), and other (n=3)

For peer review only

Supplementary Table II. Characteristics of in-depth interview participants. The interviewee # is used to reference illustrative quotes in the results section. Rows are sorted by interviewee #.

Interviewee #	County	Practice setting	Sex	Age category (years)	Highest level of education	Years in practice	Position
101	Nairobi	Urban residential area	Female	20 - 24	Pharmacy Diploma	4	Staff
102	Nairobi	Urban commercial center	Male	30 - 34	Pharmacy Diploma	10	Superintendent
103	Nairobi	Urban commercial center	Male	25 - 29	Pharmacy degree	2	Staff
104	Nairobi	Urban commercial center	Female	30 - 34	pharmacy assistant certificate	8	Staff
105	Nairobi	Urban commercial center	Male	25 - 29	Pharmacy Diploma	6	Superintendent
106	Nairobi	Urban residential area	Male	35 - 39	Pharmacy Diploma	10	Superintendent
201	Mombasa	Urban commercial center	Female	25 - 29	Pharmacy Diploma	5	In-charge
202	Mombasa	Urban commercial center	Male	40 - 44	Pharmacy Diploma	10	Owner or director
203	Mombasa	Urban informal settlement	Male	35 - 39	BSc supply chain	5	Owner or director
204	Mombasa	Urban commercial center	Male	25 - 29	Pharmacy Diploma	4	Staff
205	Mombasa	Urban commercial center	Male	25 - 29	Pharmacy Diploma	4	Superintendent
206	Mombasa	Urban commercial center	Female	20 - 24	Pharmacy Diploma	2	Staff
301	Kisumu	Urban commercial center	Male	25 - 29	Pharmacy Diploma	2	Staff
302	Kisumu	Rural town/ shopping center	Female	40 - 44	Nursing certificate	17	Staff
303	Kisumu	Urban commercial center	Male	30 - 34	Pharmacy Diploma	7	Superintendent
304	Kisumu	Rural town/ shopping center	Female	35 - 39	Pharmacy Diploma	7	Superintendent
305	Kisumu	Rural town/ shopping center	Female	30 - 34	Health-related masters	9	Owner or director
306	Kisumu	Rural town/ shopping center	Male	35 - 39	Health-related masters	10	Owner or director

For peer review only

Supplementary file A – PHARMACY QUESTIONNAIRE

Note to interviewer: This questionnaire is targeted at the pharmacy service provider (PSP) previously included in the PHP study. In case they are not available, target the in-charge or most experienced or most highly-educated PSP, in that order.

BASIC INFORMATION
Interviewer identifier; Pharmacy identifier; Date; Time

INTRODUCTION
Thank you for finding time for this interview. I would like to ask you some questions regarding general operations in your pharmacy and your experience providing COVID19-related services…

SECTION I: RESPONDENT CHARACTERISTICS

1. What is your gender? Male/Female/other, specify
2. What is your age category in years?
   18 – 24
   25 – 29
   30 – 34
   35 – 39
   40 – 44
   45 – 49
   50 – 54
   55 – 59
   >59

3. What position do you hold in the pharmacy? [pharmacy owner or director/ Superintendent/ Pharmacy in-charge/ Pharm Tech/ Pharmacist/ other, specify]
4. What is your highest level of education? Pharmacy degree/ Pharmacy Diploma / Other
5. How many years have you worked in the retail/ community pharmacy sector? [numeric; if less than a year, enter 0.5]
   a. How many years have you worked in the current pharmacy?

SECTION II: OVERALL EFFECT OF THE COVID19 EPIDEMIC ON PHARMACY OPERATIONS

6. Have you had to make any changes to your operations since the outbreak of COVID19 epidemic in Kenya (mid-March 2020)? Yes/no
   a. Have you had to change your hours of operation? [can select more than one]
      No
      Reduced open hours per day, specify number of hours reduced
      Reduced open days per week, specify number of days reduced
      Closed for some time then re-opened, specify closure period in weeks
      Closed indefinitely, specify date of closure
      Other, specify
   b. Have you had to change number of staff? [select one]
      No
      Reduced
      Increased
      Other, specify
   c. Have you had to change stocking levels? [can select more than one]
      No
      Reduced stock volumes [specify 3 main items reduced in volume]
      Increased stock volumes [specify 3 main items increased in volume]

For peer review only - http://bmjopen.bmj.com/site/about/guidelines.xhtml

d. Have you had to change your meeting routine? Yes/ no
   If yes, describe [open-ended]

7. Have you observed any changes in client flow due to the COVID19 outbreak? [select one]
   No
   Decreased client flow overall
   Increased client flow overall
   Both decrease and increase in different client categories

   a. If both decrease and increase,
      i. Which client category(ies) have decreased? [open-ended]
8. Which client category(ies) have increased? [open-ended]

SECTION III: PROVISION OF COVID19-RELATED SERVICES AND PRODUCTS

9. Which COVID-related equipment and materials does your pharmacy provide? [indicate number of clients in the last one week]
   None
   Cloth masks [number clients]
   Medical masks [number clients]
   N95/ K95 masks/ respirators [number clients]
   Goggles [number clients]
   Face shields [number clients]
   Gowns/ coveralls [number clients]
   Hand sanitizer [number clients]
   Thermometers [number clients]
   Pulse oximeters [number clients]
   Other, specify type and number of clients
   Specify other COVID19-related equipment and materials that your pharmacy provides

10. Which COVID-related preventive and curative therapies does your pharmacy provide? [indicate number of clients in the last one week]
   None
   Vitamin C [number clients]
   Multivitamins [number clients]
   Traditional/ alternative/ natural remedies, specify [number clients]
   Hydroxychloroquine/ chloroquine [number clients]
   Antibiotics, specify [number clients]
   Antivirals, specify [number clients]
   Immunomodulators, specify [number clients]
   Steroids, specify [number clients]
   Other, specify type [number of clients]
   Specify other preventive and curative therapies provided

   Indicate **number of clients** who bought or requested vitamin C in the last one week
      How many of these vitamin C purchases do you think were COVID-related? [numeric]

   [same format for related subsequent questions]

11. Which COVID-related information and counselling do clients seek from your pharmacy? [indicate number of clients in the last one week]
   None

For peer review only - http://bmjopen.bmj.com/site/about/guidelines.xhtml

How to protect themselves from infection [number clients]
Symptoms to look out for if they suspect infection [number clients]
What to do if they develop symptoms or suspect infection [number clients]
Mask use and related problems [number clients]
Counselling for anxiety related to getting infected/ becoming sick with COVID19 [number clients]
Counselling for anxiety about job or business loss [number clients]
Other, specify [number clients]

Specify other type of information or counselling that clients seek: ___________________________

Indicate number of clients in the last one week who asked how to protect themselves from infection

Indicate number of clients in the last one week who asked about symptoms to look out for if they suspect infection

Indicate number of clients in the last one week who asked what to do if they develop symptoms or suspect infection

Indicate number of clients in the last one week who asked about mask use and related problems

Indicate number of clients in the last one week who sought counselling for anxiety related to getting infected or becoming sick with COVID19

Indicate number of clients in the last one week who sought counselling for anxiety about job or business loss

Indicate number of clients in the last one week who sought for other type of information or counselling

12. Select the modes of communication that clients use to seek information/ counselling
    In-person (visit the pharmacy physically)
    Phone calls (including WhatsApp calls etc)
    Texts (including WhatsApp)
    Facebook/ other web-based platforms
    Other, specify

13. What records do you keep regarding COVID-related services and products? [open-ended]
14. Do you prepare any reports regarding COVID-related services and products? Yes/no
    a. To whom do you send the reports?
        Don't send (reports are for internal use)
        Ministry of health (MoH)/ government agency
        Other, specify
    b. What type of information is contained in the reports? [open-ended]

15. What challenges have you had in providing COVID-related services and products? [open-ended]
16. What would you say is your main motivation for supporting delivery of COVID-related services and products? [select one]
        Help people protect themselves/ Help prevent transmission
        Boost the profile of the pharmacy
        Make money
        It's part of my job/ I am required to do it
        Encouragement/ support from professional association (PSK/ KPA/ etc)
        Other, specify
17. How confident do you feel about providing COVID-related services and products? [select one]

For peer review only - http://bmjopen.bmj.com/site/about/guidelines.xhtml
For peer review only

Very uncertain/ uncertain/ confident/ very confident

18. What additional support do you feel pharmacies require in provision of COVID-related services and products? [open-ended]
19. If you don't provide COVID-related products or services,
    a. Do you get clients who ask for COVID-related services or products? Yes/no
        i. If yes, what help do you offer them? Referral/ other
            1. If you refer, to where? Another pharmacy/ clinic/ Hospital/ Lab/ other
    b. What COVID-related services or products do clients ask for?
        Personal protective equipment and materials
        Preventive and curative therapies
        Information/ counselling on COVID19
        Other, specify
    c. Select the type of information/ counselling that clients seek, and indicate number of clients in the last one week [select one]
        How to protect themselves from infection [number clients]
        Symptoms to look out for if they suspect infection [number clients]
        What to do if they develop symptoms/ suspect infection [number clients]
        Mask use and related problems
        Counselling for anxiety related to getting infected/ becoming sick with COVID19 [number clients]
        Counselling for anxiety about job/ business loss [number clients]
        Other, specify [number clients]
    d. Select the modes of communication that clients use to seek information, and indicate number of clients in the last one week [select one]
        In-person (visit the pharmacy physically)
        Phone calls (including WhatsApp calls etc)
        Texts (including WhatsApp)
        Facebook/ other web-based platforms
        Other, specify
20. Has any staff member been trained on COVID19?
    a. How many current staff members have been trained on COVID19?
    b. Where did the training take place? On-site/ off-site/ virtual
    c. Who provided the training? MoH/ PSK/ KPA/ Other, specify
21. When you have questions about COVID19, who do you consult? [can select more than one]
    None/ don't consult
    MoH
    Pharmacy in-charge/ manager/ colleague (s)
    Med reps
    Nearby doctor
    Other, specify
22. What is your main source of COVID19 information? [select one]
    TV/ Radio
    Webinar
    Social media
    Google search/ internet
    Scientific journals
    Colleagues/ other pharmacy
    KPA/ PSK communications
    CME/ CPD session
    MoH communications
    Other, specify

<u>SECTION IV: INFECTION PREVENTION</u>

For peer review only - http://bmjopen.bmj.com/site/about/guidelines.xhtml

23. Which infection prevention measure(s) is your pharmacy using among staff? [can select more than one]
    - None
    - Physical distancing, describe
    - Temperature monitoring
    - Hand hygiene (handwashing, hand sanitizer, etc)
    - Disinfection of surfaces
    - Face masks
    - Goggles
    - Other, specify
24. Which infection control measure(s) is your pharmacy using among clients? [can select more than one]
    - None
    - Physical distancing, describe
    - Temperature monitoring before entering the pharmacy
    - Require clients to be wearing face masks before entering the pharmacy
    - Hand washing
    - Hand sanitizer
    - Use of non-touch payment methods, describe
    - Information materials and messages
    - Other, specify

    Which information materials and messages is your pharmacy providing to clients?
    - Posters/ banners
    - Leaflets/ pamphlets
    - Audio announcements
    - TV screen displays
    - Other, specify

25. Which method do staff members mainly use for cleaning their hands? [select one]
    - Handwashing
    - Hand sanitizer
    - Other, specify

    a. [If handwashing is main] In the last 5 working days,
       i. On how many days was water available for handwashing in your pharmacy? [enter value between 0 and 5]
       ii. On how many days was soap available for handwashing in your pharmacy? [enter value between 0 and 5]
    b. [If hand sanitizer is main] In the last 5 working days,
       i. On how many days was hand sanitizer available in your pharmacy? [enter value between 0 and 5]
26. What type of masks do staff use in your pharmacy? [can select more than one]
    - Cloth masks
    - Surgical/ medical masks
    - N95 or similar
    - Other, specify
27. Have staff members been trained on COVID19 infection prevention? Yes/no
    a. If yes, by who? [can select more than one]
       - MoH
       - Pharmacy in charge/ colleague
       - Other, specify
    b. Where did the training take place? [can select more than one]
       - Off-site, specify venue
       - In-person within the pharmacy

For peer review only - http://bmjopen.bmj.com/site/about/guidelines.xhtml

Remotely (virtually) when at the pharmacy
Remotely (virtually) when at home
Other, specify

28. Do you have any written guidelines on COVID19 infection prevention? Yes/no
    a. If yes, describe the guidelines you have access to (e.g. source, title, etc) [open-ended]
29. How adequate do you feel the infection prevention measures in your pharmacy are? [select one]
    Very inadequate/ inadequate/ adequate/ very adequate
30. Has any staff member in your pharmacy ever tested positive for COVID19? [yes/no]
    a. How many staff members have ever tested COVID19 positive? [numeric]
31. State any other comments you have regarding COVID19 infection prevention at your workplace? [open-ended, leave blank if none]

SECTION V: VIEWS ABOUT CORONAVIRUS 2019 (COV19) TESTING IN PHARMACIES

32. Do you get clients asking for COV19 testing? Yes/no
    a. In the last one week, how many clients asked for COV19 testing? [numeric]
    b. What help do you offer clients asking for COV19 testing? COV19 tests/ Temperature measurement/ Referral/ other
        i. If referral, where do you refer? [can select more than one]
            Another pharmacy
            Public clinic/ hospital
            Private clinic/ hospital
            Private lab
            Other, specify
33. [If providing COV19 testing] What type of tests do you provide? [can select more than one]
    Antibody rapid tests, specify [unit cost]
    Antigen rapid tests, specify [unit cost]
    Laboratory tests, specify [unit cost]
    Other, specify [unit cost]
34. [If not currently providing] Do you think COV19 testing should be offered in pharmacies? Yes/no
    a. If yes, state the main reason for supporting pharmacy-based COV19 testing [select one]
        There is demand for it/ clients ask for it
        Clients shy away from health facilities
        Clients perceive pharmacies as more confidential compared to other testing facilities
        Pharmacies are open for longer hours
        Pharmacies are easily accessible
        Pharmacies provide speedier (faster) services
        Other, specify
    b. If no, state the main reason for NOT supporting pharmacy-based COV19 testing
        There is no demand for it
        No privacy in pharmacies
        Might be misused
        Other, specify
35. State any other comments you have regarding provision of COV19 testing in retail pharmacies? [open-ended, leave blank if none]

SECTION VI: OTHER VIEWS AND EXPERIENCES

36. How prepared do you feel about handling a client who presents to the pharmacy with COVID19 symptoms? [select one]
    Very unprepared/ unprepared/ prepared/ very prepared
37. Which is the main concern you are facing related to personal health and wellbeing? [select one]
    None
    Exposure to infection at work

For peer review only - http://bmjopen.bmj.com/site/about/guidelines.xhtml

Lack of transportation to get to work
Being caught up with the curfew
Keeping my family safe
Meeting financial needs for me and my family
Other, specify

38. How much do you feel your personal role as a health worker in the COVID19 response is valued by the community you are serving?
    Not at all valued/ little valued/ highly valued/ very highly valued
39. What other inputs can pharmacies (uniquely) contribute to the national response to the epidemic?? [open-ended]
40. State any other comments you have regarding pharmacy practice during the COVID19 pandemic? [open-ended, leave blank if none]

For peer review only

For peer review only - http://bmjopen.bmj.com/site/about/guidelines.xhtml

Supplementary file B - Simulated client script

Call-in simulation

- Look up the pharmacy on Google Maps and note basic operational details, such as opening hours or COVID19-related client interactions. Take note of any COVID-related messaging.
- Call the customer service number on the Google Maps profile, or if no profile or no number provided, try a simple google search, otherwise contact the study coordinators. They will check if the number in the database is personal or official; if personal further discussion is needed to assess risk of detection.
- **Ask if they have medicines that you can use to protect yourself from getting COVID19, using appropriate language** as agreed during the training. Avoid prompting for information unless it is in the script.
- Make a mental note of **questions asked or advice given**. Encourage the provider to talk by listening actively and saying "don't know" if the provider asks, for example, if you know how to prepare ginger home remedy.
    - If asked why you are asking for the medicines, say that your job requires interaction with customers, some of who don't wear masks
    - If asked where you are located, mention a name of a place near the pharmacy
- If any products are recommended, ask and **write down the names.** You can also ask for the price to keep the conversation natural, but the price data is not being collected
- Ask the service provider **how you can get the medicines**. List down the **options given**, and then if "pick up from the pharmacy" is one of the options, mention that you would rather not visit the pharmacy physically, because you are afraid of getting exposed to COVID19.
- If not told spontaneously,
    - ask what **symptoms you should look out for** if you suspect COVID19 infection? If asked what symptoms you have, emphasize you don't have symptoms but just want to be ready.
    - ask **what you should do if you suspect you have COVID19 infection**?
- Thank the service provider and mention that you will get back to them to arrange how to get the medicines.

Completing the debrief questionnaire

- The debrief questionnaire should ideally be completed within 30 minutes of the simulation, while memory is still fresh. In any case, do not call another pharmacy before the previous debrief interview has been done.
- It is **very important to say honestly how the call went**, even if you feel like it didn't go very well. The **study is assessing the pharmacy and not you**, the simulated client.
- Try to capture the responses to open-ended questions close as possible to what the provider says (verbatim)
- If there is follow-up interaction, you can leave the questionnaire "unsubmitted" and finish later. Open a fresh page if you need to do another pharmacy
- If further information comes in after you have submitted, relay to the study coordinators who will then edit the record from the back end.

Ver 1.2; 08 dec 2020

For peer review only

For peer review only - http://bmjopen.bmj.com/site/about/guidelines.xhtml

Supplementary file C - debrief questionnaire

Basic data

- Pharmacy identifier
- Simulated client identifier
- Simulation type: in-person/ call-in

Call-in simulation

- What time did you call the pharmacy? [24-hour format]
- What was the gender of the person who served you? Male/ female/ could not tell
- Were any medicines recommended? Yes/no
- If yes, which medicines were recommended for prevention? [Indicate price for each]
    - None
    - Vitamin C
    - Zinc
    - Multivitamins
    - Azithromycin,
    - Other antibiotics (Augmentin, Amoxil, ceftriaxone, etc)
    - Herbal medicine, specify
    - Home remedies, specify
    - Other, specify
- What options were given on how you can get the recommended medicines?
    - None
    - Come to the pharmacy and pick
    - Send someone to the pharmacy to pick
    - The pharmacy to deliver to client's location
    - From nearest chemist/ health facility
    - Other, specify
- If no medicines recommended, what was recommended?
    - None
    - Mask use
    - Hand hygiene (washing, sanitizer)
    - Other, specify
- Were you asked why you are asking for the medicines? (yes/no)
- Before prompting, were you told the symptoms to look out for if you suspect COVID19 infection? Yes/no
    - If yes, describe [open-ended]
- After prompting, what symptoms were you told to look out for if you suspect COVID19 infection? [open-ended]
- Before prompting, were you told what to do if you suspect you have COVID19 infection? Yes/no
    - If yes, what was recommended if you suspect COVID19 infection?
        - Medications
        - Visit nearest health facility/ go see a doctor
        - Check for information online/ WHO website
        - Get tested for COVID19
        - Get checked for oxygen level
        - Get checked for body temperature
        - Self-quarantine/ isolate at home
        - other, specify
    - If medications, what medications were recommended for treatment?
        - Steriods (dexamethasone, betamethasone, prednisolone, etc)
        - Azithromycin,

Ver 1.3; 08 dec 2020

For peer review only

        - Other antibiotics (Augmentin, Amoxil, ceftriaxone, etc)
        - Painkillers (paracetamol, brufen, etc)
        - Herbal medicine, specify
        - Home remedies, specify
        - Other, specify
- After prompting, what were you told to do if you suspect you have COVID19 infection? Medications
        - Nothing
        - Visit nearest health facility/ go see a doctor
        - Check for information online/ WHO website
        - Get tested for COVID19
        - Get checked for oxygen level
        - Get checked for body temperature
        - Self-quarantine/ isolate at home
        - other, specify
    - If medications, what medications were recommended for treatment?
        - Steriods (dexamethasone, betamethasone, prednisolone, etc)
        - Azithromycin,
        - Other antibiotics (Augmentin, Amoxil, ceftriaxone, etc)
        - Painkillers (paracetamol, brufen, etc)
        - Herbal medicine, specify
        - Home remedies
        - Other, specify
- What other advice was given?
        - None
        - Wear a mask
        - Sanitize/ wash hands
        - Eat fruits
        - Other, specify
- What other questions were you asked? [open-ended]
- About how long was the call? [mins]
- Was there further interaction after the call? Yes/no
    - If yes, describe
        - SMS
        - Whatsapp
        - Follow-up call
        - Other, specify

<u>General observations</u>

- How friendly (approachable) was the person (s) who served you?
  1 – Very unfriendly
  2 – Unfriendly
  3 – Friendly
  4 – Very friendly
- How enthusiastic was the provider about providing the service?
  1 – Very unenthusiastic
  2 – Unenthusiastic
  3 – Enthusiastic
  4 – Very enthusiastic
- Did the provider say or do anything that you found particularly helpful or nice? Yes/no
  If yes, describe [free text]
- Did the provider say or do anything that you found particularly NOT helpful or NOT nice? Yes/no
  If yes, describe [free text]

Ver 1.3; 08 dec 2020

For peer review only

- Do you have any additional observations about the interaction? Yes/no
  If yes, describe [free text]

Ver 1.3; 08 dec 2020

For peer review only

Supplementary file D - In-depth Interview Topic Guide:

Assessing the response to the COVID-19 epidemic by private retail pharmacies in Kenya.

Notes to interviewer:

1. As first option, use Teams platform with video on. If internet is slow, try Teams without video and if that still doesn't work then use plain old telephone (POT).
2. If using POT, put the phone on speaker and record on a standard audio recorder placed next to the phone, and if using Teams, record directly through the application.
3. When setting up the interview, ask about internet access, preference of platform, etc. Inform the participant that they will need to be in a quiet place for about 30-60 minutes and that earphones are preferred to speaker mode.
4. Information required from the questionnaire:
    a. Preparedness level in handling COVID suspect case
    b. Products provided
    c. Information and counseling provided
    d. Whether they get clients who ask for testing
    e. Whether they think testing can be offered in a pharmacy

We would like to understand how the pharmacy sector is responding to the COVID19 epidemic, including: initial impact, experiences providing services during the epidemic, and views about pharmacy-based COVID19 testing. Towards the end of the interview I will also ask you a few questions about your experience being interviewed remotely compared to face-to-face.

Interviewee profile

1. Briefly tell me about your pharmacy (setting, staff, clients)?
    - What is your role in the pharmacy?

Initial response and impact

2. When did you first hear about COVID19? From who?
    - What did you think and feel about it at that time?

3. What was the initial response at your pharmacy?

4. What was the effect of the epidemic on pharmacy operations?
    - Client flow, sales, working hours, staff, meeting routine, etc

5. What has been your role in responding to the epidemic?
    - You said your preparedness in handling a COVID-19 case was: __________________. Please explain further
    - What aspects of your education and previous experience helped you in responding to the outbreak?

COVID-related products and services

6. In the questionnaire, you mentioned a number of products that your pharmacy is providing in relation to the epidemic. ____
Which of these products were introduced specifically because of the epidemic?
    - You also mentioned information and counseling that clients are seeking or you are providing? ____
    - How is this done?
    - How does your pharmacy handle suspected COVID19 cases?

7. Have you been involved in any COVID training or peer learning sessions, either as a participant or a trainer?

Version 1.4; 19feb2021

For peer review only - http://bmjopen.bmj.com/site/about/guidelines.xhtml

- If yes, Where? When? Who else was included?
  - Was it stand alone training on this topic or part of a wider training?
  - Do you feel the training was adequate?
  - Who was supporting the training (financially and technically)?
- If no, how did you learn how to provide COVID services and products?

8. What do you think is needed to improve the delivery of COVID-related services in the retail pharmacy sector?
   - What support (material or financial) can be given by the government or any other external party? (e.g., drugs and other supplies/ toolkits/ job-aids/ digital systems)
   - Do you report information on COVID-related services to the government or any other external party?

<u>Pharmacy-based COVID19 testing</u>

9. If they get clients who ask for COVID19 testing: YES NO (*circle one*)
   - What type of clients ask for testing? (reasons for wanting to test, etc)
   - How do you help clients asking for COVID19 testing?
   - Why do you think they prefer pharmacies rather than health facilities or other testing sites?

10. You mentioned that COVID19 testing SHOULD/ SHOULD NOT *(circle one)* be provided in pharmacies? Explain.
    - What would be needed to ensure successful implementation of COV19 testing in the retail pharmacy sector?
    - What type of tests would be most suited for pharmacy-based COVID19 testing?

<u>Other potential inputs into the response</u>

11. What other inputs can pharmacies (uniquely) contribute to the national response to the epidemic?
    - How would this be done?

Conclusion of main interview: We have come to the end of the specific questions I had. Do you have any further comments?

<u>Experience doing the interview remotely</u>

Now I would like to ask you a few questions about your experience being interviewed remotely compared to face-to-face.

12. How would you describe your experience doing the interview remotely, compared to face-to-face?
    - Have you done a face-to-face interview before? Describe.
    - Advantages and challenges
    - Would you have preferred a face-to-face interview? Explain.

13. What did you like or not like about the Teams platform?
    - Do you have previous experience with digital meeting applications? (Teams, Zoom, etc)
    - Would you have preferred another platform?

Conclusion: Thank you very much for your time. If it is ok with you, I may contact you again for any clarifications or to hear about additional experiences and views on the topic. We will share a report once data analysis is complete.

Version 1.4; 19feb2021

For peer review only

For peer review only - http://bmjopen.bmj.com/site/about/guidelines.xhtml

Supplementary file E - Checklist – Consolidated criteria for reporting qualitative studies (COREQ)[i]

Manuscript: Response to the coronavirus disease 2019 (COVID-19) pandemic at private retail pharmacies in Kenya: a mixed methods study

Item number	Guide questions/description	Reported on Page #
**Domain 1: Research team and reflexivity**		
Personal Characteristics		
1. Inter viewer/facilitator	Which author/s conducted the interview or focus group?	4
2. Credentials	What were the researcher's credentials? E.g. PhD, MD	The names and affiliations are included on title page
3. Occupation	What was their occupation at the time of the study?	The names and affiliations are included on title page
4. Gender	Was the researcher male or female?	One female, one male
5. Experience and training	What experience or training did the researcher have?	The names and affiliations are included on title page
Relationship with participants		
6. Relationship established	Was a relationship established prior to study commencement?	4- Participants had been included in a previous study
7. Participant knowledge of the interviewer	What did the participants know about the researcher? e.g. personal goals, reasons for doing the research	5 – participants signed a consent form that explained the research goals
8. Interviewer characteristics	What characteristics were reported about the inter viewer/facilitator? e.g. Bias, assumptions, reasons and interests in the research topic	No bias to report
**Domain 2: study design**		
Theoretical framework		
9. Methodological orientation and Theory	What methodological orientation was stated to underpin the study? e.g. grounded theory, discourse analysis, ethnography, phenomenology, content analysis	5- thematic analysis
Participant selection		
10. Sampling	How were participants selected? e.g. purposive, convenience, consecutive, snowball	4- purposive
11. Method of approach	How were participants approached? e.g. face-to-face, telephone, mail, email	4- telephone
12. Sample size	How many participants were in the study?	5 – 18 participants
13. Non-participation	How many people refused to participate or dropped out? Reasons?	Participatin rate was 56% of 32 providers invited; 7 of those invited did not respond after three reminders, 5 said they were not interested, and 2 had already left the pharmacy

For peer review only

Item number	Guide questions/description	Reported on Page #
		Not included due to word limit
Setting		
14. Setting of data collection	Where was the data collected? e.g. home, clinic, workplace	4-phone
15. Presence of non-participants	Was anyone else present besides the participants and researchers?	No.<br><br>*Not included due to word limit*
16. Description of sample	What are the important characteristics of the sample? e.g. demographic data, date	5 – supp tab 2
Data collection		
17. Interview guide	Were questions, prompts, guides provided by the authors? Was it pilot tested?	4 – supp file D<br><br>The interview guide was updated after the first few interviews based on responses from participants and how effectively these addressed research objectives.<br><br>*Not included due to word limit*
18. Repeat interviews	Were repeat interviews carried out? If yes, how many?	No repeat interviews were done.<br><br>*Not included due to word limit*
19. Audio/visual recording	Did the research use audio or visual recording to collect the data?	5 – data management and analysis
20. Field notes	Were field notes made during and/or after the interview or focus group?	Field notes made after the interviews<br><br>*Not included due to word limit*
21. Duration	What was the duration of the interviews or focus group?	Interviews lasted a median [range] of 35 [20-92] minutes<br><br>*Not included due to word limit*
22. Data saturation	Was data saturation discussed?	To expedite data collection, all 18 interviews were completed before starting analysis or checking for saturation. During analysis, Saturation was reached after 14 interviews, but all 18 interviews were included in analysis.

For peer review only

Item number	Guide questions/description	Reported on Page #
		Not included due to word limit
23. Transcripts returned	Were transcripts returned to participants for comment and/or correction?	Transcripts were not returned to participants for comment and/or correction *Not included due to word limit*
**Domain 3: analysis and findings**		
Data analysis		
24. Number of data coders	How many data coders coded the data?	5 – data management and analysis
25. Description of the coding tree	Did authors provide a description of the coding tree?	No.<br><br>*Not included due to word limit*
26. Derivation of themes	Were themes identified in advance or derived from the data?	Analysis followed a thematic approach with initial categories based on the interview guide and emergent themes integrated<br><br>*Not included due to word limit*
27. Software	What software, if applicable, was used to manage the data?	5- NVivo 12
28. Participant checking	Did participants provide feedback on the findings?	Participants were not asked for feedback on the findings.<br><br>*Not included due to word limit*
Reporting		
29. Quotations presented	Were participant quotations presented to illustrate the themes/findings? Was each quotation identified? e.g. participant number	Yes. Results section
30. Data and findings consistent	Was there consistency between the data presented and the findings?	Yes.
31. Clarity of major themes	Were major themes clearly presented in the findings?	Yes. Results section
32. Clarity of minor themes	Is there a description of diverse cases or discussion of minor themes?	Yes. Results section

[i] Developed from:
Tong A, Sainsbury P, Craig J. Consolidated criteria for reporting qualitative research (COREQ): a 32-item checklist for interviews and focus groups. *International Journal for Quality in Health Care*. 2007. Volume 19, Number 6: pp. 349 – 357

For peer review only

STROBE Statement—Checklist of items that should be included in reports of ***cross-sectional studies***

For peer review only

	Item No	Recommendation	Page No
**Title and abstract**	1	(*a*) Indicate the study's design with a commonly used term in the title or the abstract	1, 2
		(*b*) Provide in the abstract an informative and balanced summary of what was done and what was found	2
**Introduction**			
Background/rationale	2	Explain the scientific background and rationale for the investigation being reported	2-3
Objectives	3	State specific objectives, including any prespecified hypotheses	3
**Methods**			
Study design	4	Present key elements of study design early in the paper	3
Setting	5	Describe the setting, locations, and relevant dates, including periods of recruitment, exposure, follow-up, and data collection	3
Participants	6	(*a*) Give the eligibility criteria, and the sources and methods of selection of participants	4
Variables	7	Clearly define all outcomes, exposures, predictors, potential confounders, and effect modifiers. Give diagnostic criteria, if applicable	4
Data sources/ measurement	8*	For each variable of interest, give sources of data and details of methods of assessment (measurement). Describe comparability of assessment methods if there is more than one group	*4*
Bias	9	Describe any efforts to address potential sources of bias	4-5
Study size	10	Explain how the study size was arrived at	4
Quantitative variables	11	Explain how quantitative variables were handled in the analyses. If applicable, describe which groupings were chosen and why	4-5
Statistical methods	12	(*a*) Describe all statistical methods, including those used to control for confounding	5
		(*b*) Describe any methods used to examine subgroups and interactions	5
		(*c*) Explain how missing data were addressed	5
		(*d*) If applicable, describe analytical methods taking account of sampling strategy	5
		(*e*) Describe any sensitivity analyses	5
**Results**			
Participants	13*	(a) Report numbers of individuals at each stage of study—eg numbers potentially eligible, examined for eligibility, confirmed eligible, included in the study, completing follow-up, and analysed	5
		(b) Give reasons for non-participation at each stage	5
		(c) Consider use of a flow diagram	n/a
Descriptive data	14*	(a) Give characteristics of study participants (eg demographic, clinical, social) and information on exposures and potential confounders	5
		(b) Indicate number of participants with missing data for each variable of interest	5
Outcome data	15*	Report numbers of outcome events or summary measures	5
Main results	16	(*a*) Give unadjusted estimates and, if applicable, confounder-adjusted estimates and their precision (eg, 95% confidence interval). Make clear which confounders were adjusted for and why they were included	6-17

For peer review only - http://bmjopen.bmj.com/site/about/guidelines.xhtml

		(*b*) Report category boundaries when continuous variables were categorized	6-17
		(*c*) If relevant, consider translating estimates of relative risk into absolute risk for a meaningful time period	6-17
Other analyses	17	Report other analyses done—eg analyses of subgroups and interactions, and sensitivity analyses	6-17
**Discussion**			
Key results	18	Summarise key results with reference to study objectives	17-20
Limitations	19	Discuss limitations of the study, taking into account sources of potential bias or imprecision. Discuss both direction and magnitude of any potential bias	20
Interpretation	20	Give a cautious overall interpretation of results considering objectives, limitations, multiplicity of analyses, results from similar studies, and other relevant evidence	17-20
Generalisability	21	Discuss the generalisability (external validity) of the study results	20
**Other information**			
Funding	22	Give the source of funding and the role of the funders for the present study and, if applicable, for the original study on which the present article is based	20-21

*Give information separately for exposed and unexposed groups.

Note: An Explanation and Elaboration article discusses each checklist item and gives methodological background and published examples of transparent reporting. The STROBE checklist is best used in conjunction with this article (freely available on the Web sites of PLoS Medicine at http://www.plosmedicine.org/, Annals of Internal Medicine at http://www.annals.org/, and Epidemiology at http://www.epidem.com/). Information on the STROBE Initiative is available at www.strobe-statement.org.

For peer review only

BMJ Open

Experiences of and response to the coronavirus disease 2019 (COVID-19) pandemic at private retail pharmacies in Kenya: a mixed methods study

Journal:	*BMJ Open*
Manuscript ID	bmjopen-2021-058688.R2
Article Type:	Original research
Date Submitted by the Author:	27-Apr-2022
Complete List of Authors:	Mugo, Peter; KEMRI-Wellcome Trust Research Programme Nairobi, Health Economics Research Unit; KEMRI-Wellcome Trust Research Programme, Health Systems and Research Ethics Department<br>Mumbi, Audrey; KEMRI-Wellcome Trust Research Programme Nairobi, Health Economics Research Unit<br>Munene, Daniella; Pharmaceutical society of Kenya, CEO Office<br>Nzinga, Jacinta; KEMRI-Wellcome Trust Research Programme Nairobi, Health Economics Research Unit; KEMRI-Wellcome Trust Research Programme, Health Systems and Research Ethics<br>Molyneux, Sassy ; KEMRI-Wellcome Trust Research Programme, Health Systems and Research Ethics; University of Oxford, Nuffield Department of Medicine<br>Barasa, Edwine; KEMRI-Wellcome Trust Research Programme Nairobi, Health Economics Research Unit; University of Oxford, Nuffield Department of Medicine<br>Taskforce, COVID19 ; Pharmaceutical Society of Kenya (PSK), COVID-19 Response Taskforce
**Primary Subject Heading**:	Public health
Secondary Subject Heading:	Global health, Health services research, Infectious diseases
Keywords:	COVID-19, Public health < INFECTIOUS DISEASES, PRIMARY CARE, PUBLIC HEALTH, QUALITATIVE RESEARCH, Quality in health care < HEALTH SERVICES ADMINISTRATION & MANAGEMENT

SCHOLARONE™
Manuscripts

I, the Submitting Author has the right to grant and does grant on behalf of all authors of the Work (as defined in the below author licence), an exclusive licence and/or a non-exclusive licence for contributions from authors who are: i) UK Crown employees; ii) where BMJ has agreed a CC-BY licence shall apply, and/or iii) in accordance with the terms applicable for US Federal Government officers or employees acting as part of their official duties; on a worldwide, perpetual, irrevocable, royalty-free basis to BMJ Publishing Group Ltd ("BMJ") its licensees and where the relevant Journal is co-owned by BMJ to the co-owners of the Journal, to publish the Work in this journal and any other BMJ products and to exploit all rights, as set out in our licence.

The Submitting Author accepts and understands that any supply made under these terms is made by BMJ to the Submitting Author unless you are acting as an employee on behalf of your employer or a postgraduate student of an affiliated institution which is paying any applicable article publishing charge ("APC") for Open Access articles. Where the Submitting Author wishes to make the Work available on an Open Access basis (and intends to pay the relevant APC), the terms of reuse of such Open Access shall be governed by a Creative Commons licence – details of these licences and which Creative Commons licence will apply to this Work are set out in our licence referred to above.

Other than as permitted in any relevant BMJ Author's Self Archiving Policies, I confirm this Work has not been accepted for publication elsewhere, is not being considered for publication elsewhere and does not duplicate material already published. I confirm all authors consent to publication of this Work and authorise the granting of this licence.

For peer review only

Experiences of and response to the coronavirus disease 2019 (COVID-19) pandemic at private retail pharmacies in Kenya: a mixed methods study

Peter Mugo[1,3], Audrey Mumbi[1], Daniella Munene[2], Jacinta Nzinga[1,3], Sassy Molyneux[3,4], Edwine Barasa [1,3,4], on behalf of PSK CRT[5]

[1] Health Economics Research Unit, Kemri-Wellcome Trust Research Programme, Nairobi, Kenya
[2] Pharmaceutical society of Kenya
[3] Health Systems and Research Ethics Department, KEMRI-Wellcome Trust Research Programme, Kilifi, Kenya
[4] University of Oxford, Headington, UK
[5] Pharmaceutical Society of Kenya (PSK) COVID-19 Response Taskforce (CRT)

Correspondence to:
Peter Mwangi Mugo
KEMRI-Wellcome Trust Research Programme
P.O. Box 43640 – 00100 Nairobi, Kenya
Tel: + 254 730 162 000
E-mail: PMugo@kemri-wellcome.org

Keywords: Private retail pharmacies, COVID-19 response, Kenya

For peer review only - http://bmjopen.bmj.com/site/about/guidelines.xhtml

Abstract

Objectives: To assess experiences of and response to the COVID-19 pandemic at community pharmacies in Kenya.

Design, setting, and participants: This was a mixed-methods study conducted from November 2020 to April 2021, targeting service providers in three counties (Nairobi, Mombasa, and Kisumu), selected purposively to represent the main urban centres; pharmacies were selected randomly from a list of licensed pharmacies.

Results: Of 195 sampled pharmacies, 108 (55%) completed a questionnaire and 103 (53%) received a simulated client call; 18 service providers were interviewed. The initial weeks of the pandemic were characterized by fear and panic among service providers and a surge in client flow. Subsequently, 65 (60%) of 108 pharmacies experienced a dip in demand to below pre-pandemic levels and 34 (31%) reported challenges with unavailability, high price, and poor-quality of products. Almost all pharmacies were actively providing preventive materials and therapies; educating clients on prevention measures; counselling anxious clients; and handling and referring suspect cases. Fifty-nine pharmacies (55% [95% CI 45-65%]) reported receiving a client asking for COVID-19 testing and a similar proportion stated they would support pharmacy-based testing if implemented. For treatment of simulated clients, most pharmacies (71%, 73 of 103) recommended alternative therapies and nutritional supplements such as vitamin C; the rest recommended conventional therapies such as antibiotics. While 52 (48%) of 108 pharmacies had at least one staff member trained on COVID-19, a general feeling of disconnection from the national program prevailed.

Conclusions: Private pharmacies in Kenya were actively contributing to the COVID-19 response, but more deliberate engagement, support and linkages are required. Notably, there is an urgent need to develop guidelines for pharmacy-based COVID-19 testing, a service that is clearly needed and which could greatly increase test coverage. Pharmacy-based COVID-19 programs should be accompanied with implementation research to inform current and future pandemic responses.

Strengths and limitations of this study

- The study used multiple quantitative and qualitative methods to enhance the comprehensiveness and reliability of the findings.
- We used simulated clients to objectively assess provider practices, an approach that is gaining acceptance as a gold standard for the measurement of clinical practice quality around the world.
- The study took advantage of information collected in a recent study to speed up recruitment and data collection.
- One weakness of the study was the low response rate (55%), considering that it targeted pharmacies that had participated in a similar study less than a year previously.

For peer review only - http://bmjopen.bmj.com/site/about/guidelines.xhtml

Introduction

The coronavirus disease 2019 (COVID-19) pandemic is an ongoing global health emergency [1, 2]. As of September 2021, over 230 million cases had been confirmed globally resulting in over 4.7 million deaths [3]. For the better part of 2020, non-pharmaceutical interventions such as early case detection, contact reduction, and hygiene measures were the main prevention strategies [4, 5]. In early 2021, following unprecedented public-private collaboration and speed in research and development, a number of highly effective vaccines became available [6, 7].

Non-pharmaceutical supportive care remains the key approach to treatment and management [8-10]. Among hospitalized patients, pharmaceuticals that have been shown to be effective include corticosteroids such as dexamethasone, antivirals such as remdesivir, and immunomodulators such as tocilizumab [8, 11, 12]. In the outpatient setting, pharmaceuticals used to manage mild COVID-19 cases include those targeting fever and pain such as ibuprofen, and those aiming to relieve respiratory congestion [9][12]. Other more specific agents that have been tried, such as hydroxychloroquine, ivermectin, and azithromycin, are discouraged [9, 12]. The World Health Organization and the US National Institutes of Health recommend that such unproven therapies be used only within the context of clinical trials [13, 14].

It is predicted that follow-up waves of COVID-19 infection may continue indefinitely [15]. Strategies are therefore required to enhance and sustain coverage, cost-effectiveness, and efficiency of prevention and treatment interventions. Private retail pharmacies (also referred to as community pharmacies) are a unique channel through which COVID-19 prevention and treatment interventions can be delivered, since they are often the first or only point of contact with the healthcare system in developing countries [16-18]. The main reasons for preferential care-seeking at pharmacies, compared to health facilities, include greater accessibility, lower cost, and greater perceived privacy [19, 20]. The International Pharmaceutical Federation highlights key areas where the pharmacy sector can support the pandemic response, including: management of the medication supply chain, patient education, identification and referral of patients to other health care providers, and provision of prevention materials and therapies [21, 22]. During the COVID-19 pandemic, pharmacies in many settings, including Kenya, were categorized as essential services hence they were exempted from closure [23].

The first COVID-19 case in Kenya was reported on 13th March 2020, and as of 12th October 2021, there were 251,248 confirmed cases and 5,190 deaths [24]. A number of control measures have been instituted by the government at different times depending on rates of infection and mortality [23, 25, 26]. Vaccine roll-out in Kenya started in March 2021 [27]. The aim of the study was to assess the preparedness, experiences, and response to the COVID-19 pandemic at private retail pharmacies in Kenya, with an overall goal of identifying strategies for maximising the contribution of pharmacies to the national response.

Methods

For peer review only - http://bmjopen.bmj.com/site/about/guidelines.xhtml

Study design

We conducted a cross-sectional descriptive study, including a questionnaire survey, a simulated client survey, and in-depth interviews. A mixed-methods approach was used in data collection and analysis: insights from interviews were used to refine the survey tools and probe quantitative data; and survey data were used to refine the interview guide and qualitative data analysis. Given the physical distancing requirements at the time, data collection was done remotely.

Study settings and populations

Community pharmacies in Kenya are generally small to medium-sized businesses providing typical pharmacy services such as filling of prescriptions, over-the counter products, and point-of-care testing or self-testing kits for common diseases. While traditionally a fragmented market, the sector is starting to consolidate. In 2019, of 5,033 licensed pharmacies, 4,286 (85%) were independent (single branch) pharmacies, 594 (12%) belonged to small chain networks (2-5 branches), and 152 (3%) were part of large chain networks (6-41 branches) belonging to 13 companies [28].

The study was conducted in three counties (Nairobi, Mombasa and Kisumu), selected purposively to represent the main urban centres, where there was high concentration of pharmacies and COVID-19 cases. As of June 2020, the three counties together contributed 32% (1,602/5,033) of licensed pharmacies [28] and 78% (2,229/2,862) of reported COVID-19 cases [29] [24].

We targeted pharmacies that had participated in a pharmacy HIV prevention (PHP) study about a year previously. Pharmacies had been selected through stratified random sampling where the required number of pharmacies for each county was picked by an independent data manager from the list of licensed pharmacies for that county using the Excel® Rand() function. The sample size for the PHP study (n=195) had been calculated to provide 80% power to detect associations at the 5% significance level, assuming a binary dependent variable split at 70% and 50% across a binary predictor variable. The sample size achieved in the current study (n=108) gives 57% power for similar parameters and assumptions, and 80% power for a 75%-50% split. Respondents were pharmacists (bachelor's degree holders) and pharmaceutical technologists (diploma holders). The preferred questionnaire respondent was the pharmacy-in-charge, the most experienced staff member or the most highly educated, in that order. Interview participants were purposively selected from among questionnaire respondents, targeting those that showed enthusiasm for the study through prompt and detailed responses. We aimed to increase variability of interview data by stratifying the sample by county, practice setting (urban vs rural) and gender (male vs female).

Data collection

Data collection started in November 2020 (approximately seven months after the pandemic reached Kenya) and was concluded in December 2020 (for the surveys) and April 2021 (for the interviews).

For peer review only - http://bmjopen.bmj.com/site/about/guidelines.xhtml

We used previously obtained phone numbers to contact sampled pharmacies, obtain informed consent and share a link to an online questionnaire. One questionnaire, taking about 20-30 minutes, was completed by each participating pharmacy (**Supplementary file A**). Up to three reminder phone calls were made. Data on pharmacy characteristics were imported from the PHP study database.

Simulated clients (SCs) made unscheduled phone calls to participating pharmacies, mimicking a client seeking COVID-19 preventive therapies (**Supplementary file B**). The SCs (2 women and 2 men, aged 25-35 years) had previously been engaged in the PHP study and trained comprehensively over a 2-week period. For the current study, further training was provided over a 1-week period through the Teams® digital meeting platform. An online debrief questionnaire was used to record observations (**Supplementary file C**). All calls were made on weekdays (Monday – Friday) between 8am and 5pm, over a 3-week period. Median [range] duration of calls was 4 [1-12] minutes. The majority (68%) of calls were made to a publicly available phone number, while the rest used a number in the study database as there was no public number. A few weeks after the simulated call, participating pharmacies were contacted to check if they suspected any clients visiting the pharmacies during the survey period to be "fake" (detection survey).

In-depth interviews were conducted by the first and second authors using a semi-structured interview guide (**Supplementary file D**). Interviews were conducted via the Teams® digital meeting platform as a first option or a simple phone call if the participant so preferred or if internet connectivity was poor.

Data management and analysis

Quantitative data were captured directly into an open-source database (REDcap®, Vanderbilt University). Data cleaning and analysis was carried out using Stata® version 15 (College Station, Texas, USA). Summary statistics were compared across counties using the chi square test or analysis of variance for means. Exploratory multivariable modelling was done using logistic regression. Variables with $p<0.10$ in bivariate analysis were included in the multivariable model and those with $p<0.05$ in the multivariable model were considered to be statistically significant. Systematicity in detection was assessed by correlating detection status with caller, pharmacy characteristics, and simulation process features.

Qualitative interviews were audio-recorded and transcribed verbatim in Microsoft Word®, translating into English where necessary. To ensure quality, all transcripts were cross-checked against the audio recordings. Transcripts were imported into NVivo 12 (SR International, Australia) and coded by the first and second authors. Analysis followed a thematic approach with initial categories based on the interview guide and emergent themes integrated in a second

For peer review only - http://bmjopen.bmj.com/site/about/guidelines.xhtml

round of coding. A checklist on Consolidated criteria for Reporting Qualitative research (COREQ) was completed (**Supplementary file E**).

Ethical considerations

The study was approved by the Kenya Medical Research Institute (KEMRI) Scientific and Ethics Review Unit (KEMRI/ SERU/CGMR-C/208/4080). Participants signed written informed consent forms administered digitally.

Patient and public involvement

An early version of the study protocol received inputs from the Pharmaceutical Society of Kenya (PSK) COVID-19 response taskforce composed of pharmacy sector stakeholders. Preliminary findings were shared with the pharmacy sector at the 2021 PSK Annual Conference and a policy brief has been distributed to stakeholders in the ministry of health, pharmacy sector and other implementing partners. Findings were also discussed in a local Radio show in February 2022.

Results

Response rate and characteristics of participants

Of 195 target pharmacies, 108 (55%) completed a questionnaire. Participating pharmacies were generally similar to non-participating pharmacies, but had features indicative of higher operational capacity and interest in public health interventions, such as having a computerized stock management system, written job descriptions, and providing blood pressure measurement (data not shown). Ninety-four (87%) pharmacies were located in urban areas; 44 (41%) had a consultation room; 71 (67%) had a computerised stock management system; and 13 (12%) were part of a chain network (**Supplementary table Ia**). The majority of questionnaire respondents were male, below 40 years, and had a pharmacy diploma (**Supplementary table Ib**).

One hundred and three (95%) pharmacies received a simulated client call. Thirteen (13%) calls were determined as probably detected. Detection was less likely if the call was made by one particular caller (perfect prediction), gender of person answering the call was male (adjusted odds ratio, aOR, 0.1 [95% Confidence interval, CI, 0.0004-0.4, p=0.01); the pharmacy had a publicly available phone number (aOR 0.01 [95% CI 0.0006-0.4], p=0.01), was part of a chain network (perfect prediction), opened 24 hours daily (perfect prediction), or had an on-site laboratory (perfect prediction). Main call outcomes (preventive therapies recommended, options for delivery of recommended products, and referral options) did not vary by detection status (data not shown), therefore all data were included in analysis.

Of 18 interview participants, 11 (61%) were male, 16 (88%) were below 40 years, 11 (61%) were pharmacy owner or in-charge, and 12 (67%) were diploma holders. Number of participants was

For peer review only - http://bmjopen.bmj.com/site/about/guidelines.xhtml

balanced by county (6 each), but was skewed towards urban setting (13 vs 5) and male gender (11 vs 7) (**Supplementary Table II)**.

Impact of the pandemic on service providers and pharmacy operations

Interview participants described fear and panic when they first heard about COVID-19, especially regarding the risk of exposure to infection:

> *"In the beginning, it sent everybody into a panic mode. Including my entire staff where everybody now thought this is a killer disease and being healthcare workers, they must be people who will be among the victims."*
>
> - Male pharmaceutical technologist, Nairobi, 105

Some providers were in denial, feeling that the pandemic would never reach *"where we are"*, and others reported that some clients believed the pandemic was "*just an avenue maybe for our politicians ready to make some money or something*".

The main concerns reported in the questionnaire were: exposure to infection at work (66% of respondents), "keeping my family safe" (22%), meeting financial needs (6%), and being caught up in the curfew (3%).

One hundred (93%) of 108 pharmacies reported pandemic-induced operational challenges (**Figure 1**). The main supply-side challenge was unavailability and high price of products and the main demand-side challenge was reduced client flow.

Interview data corroborated survey findings. Interviewees described temporal changes in client flow, with an initial surge of clients buying vitamins and other immune boosters, followed a few weeks later by a dip in demand to below pre-pandemic levels. The initial surge was accompanied by an increase in self-medication and stockpiling of long-term medications, such as for diabetes. While some of these requests were deemed potentially inappropriate, providers were not always able to effectively intervene:

> *"They just keep [these drugs] in case. …Like there was one [client] who came with a running nose, and then he was telling me he wanted Zithromax [a brand of the antibiotic azithromycin]. 'Get Flu gone, get antihistamine, this one will help you' [I told him]. So sometimes as a primary healthcare provider, you try to advise them, but now they have made up their mind, so you just go ahead and let them go [somewhere else]. Because whether you refuse, you do what, they've made up their mind."*
>
> - Male pharmaceutical technologist, Kisumu, 303

Participants attributed the subsequent reduction in demand mainly to the dusk-to-dawn curfew, despite the fact that pharmacy providers were classified as essential workers and issued curfew passes:

For peer review only - http://bmjopen.bmj.com/site/about/guidelines.xhtml

"Of course, the curfew really beat us down, because now by the time [it's] seven o'clock, people are going home… and it's our prime time to serve clients as from 7 pm. So, we as well missed out on business."

- Male pharmacy owner, Mombasa, 203

A number of interviewees believed that the decrease in service utilization was due to fear of infection and quarantine among clients:

"Then people shied off from coming to the pharmacy especially the ones with coughs and flus because they were just thinking that I could tell them to go to hospital for the corona tests. So most clients decided to stay at home, not to come to the pharmacy for consultation, or they just sent somebody to buy them the drugs"

- Female pharmaceutical technologist, Kisumu, 304

One interviewee saw the stockpiling by clients in positive light, observing that it enabled him to offload slow-moving products:

"People resorted to scavenging where they might find these products and so that way [one was] able to liquidate what was lying in your shops"

- Male pharmaceutical technologist, Nairobi, 105

A reduction in hygiene-related illnesses was also hailed as a positive public health effect of the COVID-19 preventive measures:

"Due to proper maintenance of hygiene, the short-short illnesses have reduced. Washing of hands and stuff.""

- Male pharmacist, Nairobi, 103

Adjustments to pharmacy operations

Pharmacies adjusted their operations to cope with these challenges and to accommodate the government-instituted prevention measures: 74% reduced opening hours (mean 3 hours [range 1-7]); 38% reduced staffing; and 30% changed their meeting routine (**Table 1**). Adjustments did not vary significantly across counties.

Table 1. Adjustments to pharmacy operations following the outbreak of COVID-19 in Kenya

Change	Nairobi (N=38)		Mombasa (N=36)		Kisumu (N=34)		Total (N=108)	
	n	%	n	%	n	%	n	%
**Opening hours**[1]								
No change	7	18%	8	22%	10	29%	25	23%
Reduced open hours per day	31	82%	25	69%	24	71%	80	74%
Reduced open days per week	1	3%	4	11%	0	0%	5	5%
Closed temporarily	0	0%	0	0%	1	3%	1	1%
**Staffing**								

For peer review only - http://bmjopen.bmj.com/site/about/guidelines.xhtml

Change	Nairobi (N=38)		Mombasa (N=36)		Kisumu (N=34)		Total (N=108)	
No change	19	50%	25	71%	19	59%	63	60%
Reduced number of staff	18	47%	9	26%	13	41%	40	38%
Increased number of staff	0	0%	1	3%	0	0%	1	1%
Reduced pay	1	3%	0	0%	0	0%	1	1%
**Stock volume levels**								
No change	12	32%	13	36%	8	24%	33	31%
Reduced overall	8	21%	12	33%	12	35%	32	30%
Increased overall	14	37%	10	28%	14	41%	38	35%
Both reduced and increased for different items	4	11%	1	3%	0	0%	5	5%
**Meeting routine**								
No change	24	63%	30	83%	22	65%	76	70%
Resorted to virtual/ remote/ online meetings[2]	8	21%	3	8%	7	21%	18	17%
Reduced frequency of meetings	4	11%	1	3%	3	9%	8	7%
Stopped meetings altogether	2	5%	1	3%	0	0%	3	3%
Reduced duration/ number of people	0	0%	1	3%	1	3%	2	2%
Changed timing	0	0%	0	0%	1	3%	1	1%

[1] Three pharmacies in Mombasa and one in Nairobi reduced their open days per week by one, while one pharmacy in Mombasa reduced by three days. One pharmacy in Kisumu closed temporarily for 2 weeks

[2] Main virtual platforms used included Zoom, Google Meet, and Whatsapp

COVID-19-related products and services provided by pharmacies

Table 2 presents the COVID-19-related products and services that were being provided or requested at pharmacies. Equipment and materials in highest demand were medical masks, N95/ K95 respirators, face shields, hand sanitizer, and thermometers; while the medications in highest demand were vitamin C, multivitamins, and antibiotics. Only 24 (22%) pharmacies reported keeping records related to COVID-19 services.

Table 2. COVID-19-related products and services provided by private retail pharmacies in Kenya

Data are number (%) of pharmacies providing or receiving requests (main row) and number of clients in past week (subsidiary row).

Product/ service	Nairobi (N=38)		Mombasa (N=36)		Kisumu (N=34)		Total (N=108)	
	N or median	% or IQR	N or median	% or IQR	N or median	% or IQR	N or median	% or IQR
**Equipment and materials**								
Cloth masks	1	3%	5	14%	6	18%	12	11%
Number of clients in past week	20	20-20	20	4-30	10	5-15	15	5-25
Medical Masks	37	97%	33	92%	30	88%	100	93%
Number of clients in past week	50	20-100	23	10-80	26	20-50	30	20-70

For peer review only - http://bmjopen.bmj.com/site/about/guidelines.xhtml

Product/ service	Nairobi (N=38)		Mombasa (N=36)		Kisumu (N=34)		Total (N=108)	
	N or median	% or IQR	N or median	% or IQR	N or median	% or IQR	N or median	% or IQR
N95/ K95 masks	24	63%*	18	50%	11	32%	53	49%
Number of clients in past week	5	4-16	5	2-30	1	0-2	5	2-10
Goggles	6	16%	2	6%	3	9%	11	10%
Number of clients in past week	0	0-10	6	2-10	1	0-5	1	0-10
Face shields	19	50%*	10	28%	8	24%	37	34%
Number of clients in past week	1	0-6	1	0-3	1	0-8	1	0-5
Gowns/ coveralls	1	3%	1	3%	3	9%	5	5%
Number of clients in past week	0	0-0	10*	0-10	0	0-1	0	0-1
Hand sanitizer	37	97%	35	97%	31	91%	103	95%
Number of clients in past week	10	3-30	5	2-13	5	2-13	5	3-15
Thermometers	27	71%	23	64%	20	59%	70	65%
Number of clients in past week	2	1-5	2	1-5	1	0-2	2	2-3
Pulse oximeters	10	26%	11	31%	3	9%	24	22%
Number of clients in past week	1	1-3	2	0-10	1	0-2	2	1-3
**Medicines**								
Vitamin C[1]	35	92%*	29	81%	23	68%	87	81%
Number of clients in past week	10	10-30	15	10-30	5	3-11	10	4-20
Multivitamins[1]	29	76%	30	83%	24	71%	83	77%
Number of clients in past week	10	5-19	10	6-20	10	7-18	10	6-20
Traditional/ alternative/ natural remedies[1, 2]	1	3%	2	6%	0	0%	3	3%
Number of clients in past week	0	0-0	13	5-20	0	0-0	5	0-20
Hydroxychloroquine/ chloroquine	8	21%	7	19%	2	6%	17	16%
Number of clients in past week	3	2-7	1	0-5	3	0-5	2	0-5
Antibiotics[1, 3]	26	68%	22	61%	23	68%	71	66%
Number of clients in past week	3	2-10	15*	10-39	15	5-29	10	3-20
Steroids[1]	16	42%	16	44%	16	47%	48	44%
Number of clients in past week	3	1-8	9	5-19	10	4-15	7	3-15
Other medicines[4]	3	8%	4	11%	2	6%	9	8%
Number of clients in past week	3	1-5	28	23-45	3	1-4	13	3-28
**Information and counselling**								
How to protect themselves from infection	28	74%	28	78%	27	79%	83	77%
Number of clients in past week	5	2-13	5	3-15	10	3-20	5	3-15
Symptoms to look out for if they suspect infection	32	84%	28	78%	26	76%	86	80%
Number of clients in past week	3	2-10	5	4-10	9	2-11	5	2-11
What to do if they develop symptoms/ suspect infection	22	58%	21	58%	16	47%	59	55%
Number of clients in past week	3	2-7	3	2-5	5	3-22	4	2-10
Mask use and related problems	19	50%	19	53%	20	59%	58	54%
Number of clients in past week	5	2-10	5	2-35	15	4-20	7	2-20
Counselling for anxiety related to getting infected/ becoming sick with COVID-19	14	37%	16	44%	13	38%	43	40%
Number of clients in past week	2	1-10	3	1-10	3	1-5	3	1-8

For peer review only

Product/ service	Nairobi (N=38)		Mombasa (N=36)		Kisumu (N=34)		Total (N=108)	
	N or median	% or IQR	N or median	% or IQR	N or median	% or IQR	N or median	% or IQR
Counselling for anxiety about job/ business loss	5	13%	7	19%	5	15%	17	16%
Number of clients in past week	2	2-8	2	1-5	5	3-8	3	2-8
**Modes of communication with clients**								
Face-to-face at the pharmacy	33	87%	33	92%	34	100%	100	93%
Phone calls (including WhatsApp calls etc)	21	55%	25	69%	20	59%	66	61%
Texts (including WhatsApp messages, etc)	13	34%	15	42%	12	35%	40	37%
Facebook/ other web-based social media platforms	3	8%	2	6%	2	6%	7	6%

IQR: interquartile range
** Indicates significant difference between counties ($p<0.05$), based on chi square test or analysis of variance for means*
[1] A significant proportion of these sales may not have been related to COVID-19 prevention/ treatment
[2] Traditional/ alternative/ natural remedies included echinacea, chapa shoka, kaluma inhaler, menthol plus balm, and home remedies (lemon, papaya steaming)
[3] Antibiotics included azithromycin (n=55), cephalosporin (n=7), amoxicillin-containing (n=6), other macrolide (n=2); 3 pharmacies did not specify
[4] Other medicines included zinc+ vitamin D (n=4), vitamin B complex (n=1), salbutamol (n=1), and scotts emulsion (n=1); 2 pharmacies did not specify

Interview data suggested that many of these products and services were newly introduced following the outbreak, including products like vitamin C and surgical masks that one would have expected to find routinely in a pharmacy.

Demand for COVID-19 testing and provider views towards pharmacy-based testing

Fifty-nine (55% [95% CI 45-65%]) of 108 pharmacies reported ever receiving a client asking for COVID-19 testing, with a median [interquartile range] number of requests in the last week of 2 [1-4]. At the time of the survey, none of the pharmacies was providing COVID-19 testing; and among the 59 pharmacies experiencing demand for testing, alternatives given to clients included referral to other testing sites (94%) and body temperature measurement (15%). Referral options included: public health facilities (92%), private health facilities (27%), and private labs (13%).

Interviews revealed that test requests were mostly by clients with symptoms, those who suspected they had been exposed, and those who needed certification for travel or employment purposes (eg, drivers and hotel staff).

When asked if they thought COVID-19 testing should be provided in pharmacies, 60% [95% CI 50-70%] said "yes". Among the 64 that were supportive of pharmacy-based testing, main reasons for support were: pharmacies are easily accessible (36%), clients perceive pharmacies as more confidential (23%), clients shy away from health facilities (19%), and demand exists (9%).

For peer review only - http://bmjopen.bmj.com/site/about/guidelines.xhtml

In interviews, the confidentiality and convenience of the pharmacy setting was reiterated, with some interviewees suggesting home-based self-testing as an option:

> *"If it [testing] is available in pharmacies, somebody can just take the kit, you can just go home, you test yourself. If you find you are positive, you can just self-quarantine, without raising an alarm. So that when the quarantine period is over, you don't get that kind of stigma, rejection."*
>
> - Female pharmacy in charge, Mombasa, 201

Among the 42 that were not supportive of pharmacy-based testing, main reasons for opposing were: might be misused (29%), no private space in the pharmacy (24%), risk of infection (15%), protective gear is expensive or not available (12%), there is no demand for the service (10%), and staff are not adequately trained (10%). From the interviews, hesitancy to support pharmacy-based testing was mainly related to lack of capacity (*"I still need a thorough training…"*) and fear of infection.

Handling of simulated clients seeking covid-19-related services

When a simulated client called a pharmacy to ask if they have medicines that the client can use to protect themselves from getting COVID-19, 76 (74%) of 103 pharmacies recommended one or more medications: 27% recommended at least one conventional therapy and 71% at least one alternative therapy or nutritional supplement (**Table 3**). The main recommendations given to a client suspecting COVID-19 infection were to: get tested, visit the nearest health facility, and self-quarantine at home.

Table 3. Handling of simulated clients seeking COVID-19-related services at private retail pharmacies in Kenya

Call outcome	Nairobi (N=38)		Mombasa (N=36)		Kisumu (N=34)		Total (N=103)	
	n	%	n	%	n	%	n	%
**Provider response to "*Do you have medicines that I can use to protect myself from getting COVID-19?*"**								
**Asked the client why he/she was asking for the medicines**	**14**	**38%***	**4**	**14%**	**2**	**6%**	**20**	**20%**
**Recommended conventional therapies**	**11**	**29%**	**6**	**19%**	**11**	**32%**	**28**	**27%**
Antibiotics	11	29%	6	19%	10	29%	27	26%
Azithromycin	9	24%	6	19%	9	26%	24	23%
Other antibiotics[1]	2	5%	1	3%	4	12%	7	7%
Painkillers	1	3%	0	0%	3	9%	4	4%
Antihistamines	0	0%	1	3%	2	6%	3	3%
**Recommended alternative therapies**	**29**	**76%**	**22**	**71%**	**22**	**65%**	**73**	**71%**
Vitamin C	28	74%	20	65%	19	56%	67	65%
Zinc	25	66%*	10	32%	7	21%	42	41%
Home remedies[2]	6	16%	4	13%	6	18%	16	16%

For peer review only - http://bmjopen.bmj.com/site/about/guidelines.xhtml

Call outcome	Nairobi (N=38)		Mombasa (N=36)		Kisumu (N=34)		Total (N=103)	
Vitamin B/ D	4	11%	4	13%	2	6%	10	10%
Multivitamins	2	5%	0	0%	3	9%	5	5%
Other remedies[3]	1	3%	0	0%	1	3%	2	2%
**Gave options on how to get the recommended medicines**	**37**	**97%**	**28**	**90%**	**34**	**100%**	**99**	**96%**
Pick from the pharmacy	21	55%	7	23%*	18	53%	46	45%
Home delivery	19	50%*	9	29%	4	12%	32	31%
Send someone to pick	5	13%	8	26%	4	12%	17	17%
**Provider response to "*What symptoms should I look out for if I suspect COVID-19 infection?*"**								
**Mentioned any symptoms**	**34**	**89%**	**29**	**94%**	**30**	**88%**	**93**	**90%**
Fever	23	61%	20	65%	21	62%	64	62%
Cough	13	34%	15	48%	16	47%	44	43%
Flu-like symptoms	15	39%	15	48%	12	35%	42	41%
Breathing difficulty	13	34%	12	39%	17	50%	42	41%
Loss/ change of smell or taste	16	42%	13	42%	11	32%	40	39%
Sore throat	15	39%*	8	26%	4	12%	27	26%
Chest pain or congestion	7	18%	4	13%	12	35%*	23	22%
Headache	6	16%	4	13%	8	24%	18	17%
Fatigue, tiredness, malaise	2	5%	8	26%*	4	12%	14	14%
Reduced appetite	2	5%	2	6%	7	21%	11	11%
Body aches/ pains	0	0%	3	10%	2	6%	5	5%
Gut symptoms, eg, stomach pain, diarrhea, etc	2	5%	0	0%	2	6%	4	4%
Dizziness	0	0%	2	6%	0	0%	2	2%
Weight loss	0	0%	0	0%	1	3%	1	1%
**Provider response to "*What should I do if I suspect I have COVID-19 infection?*"**								
**Immediate actions recommended**								
Get tested for COVID-19	25	66%	18	58%	16	47%	59	57%
Visit nearest health facility/ go see a doctor	18	47%	13	42%	24	71%*	55	53%
Self-quarantine/ isolate at home	3	8%	9	29%*	4	12%	16	16%
Take medications[4]	3	8%	2	6%	3	9%	8	8%
Check body temperature	2	5%	1	3%	2	6%	5	5%
Check oxygen levels	0	0%	1	3%	1	3%	2	2%
Check information online	0	0%	1	3%	1	3%	2	2%
Call 719[5]	2	5%	0	0%	0	0%	2	2%
**Supplementary actions recommended**								
Wear a mask	23	61%	16	52%	20	59%	59	57%
Maintain physical distance	21	55%	15	48%	18	53%	54	52%
Observe hand hygiene	7	18%	5	16%	7	21%	19	18%
Eat fruits	5	13%	5	16%	5	15%	15	15%
Eat well[6]	2	5%	3	10%	1	3%	6	6%
Take lots of fluids	1	3%	1	3%	3	9%	5	5%
Live well[7]	0	0%	0	0%	4	12%	4	4%

For peer review only

Call outcome	Nairobi (N=38)		Mombasa (N=36)		Kisumu (N=34)		Total (N=103)	
Other advice[8]	3	8%	1	3%	2	6%	6	6%
**Caller assessment of the interaction**								
**How friendly (approachable) was the person who served you?**								
1 - Very unfriendly	1	3%	0	0%	0	0%	1	1%
2 - Unfriendly	4	11%	3	10%	4	12%	11	11%
3 - Friendly	20	53%	22	71%	22	65%	64	62%
4 - Very friendly	13	34%	6	19%	8	24%	27	26%
**How enthusiastic was the provider about providing the service?**								
1 - Very unenthusiastic	1	3%	0	0%	0	0%	1	1%
2 - Unenthusiastic	4	11%	4	13%	7	21%	15	15%
3 - Enthusiastic	22	58%	22	71%	21	62%	65	63%
4 - Very enthusiastic	11	29%	5	16%	6	18%	22	21%
**Did the provider say or do anything that you found particularly helpful or nice?[9]**	**19**	**50%**	**9**	**29%**	**14**	**41%**	**42**	**41%**
**Did the provider say or do anything that you found particularly NOT helpful or NOT nice?[10]**	**4**	**11%**	**2**	**6%**	**7**	**21%**	**13**	**13%**

** Indicates significant difference between counties (p<0.05), based on chi square test*

[1] Other antibiotics included Augmentin, Amoxil, ceftriaxone, etc

[2] Home remedies included ginger/ lemon/ hot water drink, hot soup/ water, garlic, steam, frequent hot baths

[3] Other alternative therapies included ginsomin and omega-3

[4] Medications for treatment included: azithromycin (3%), other antibiotics (4%), painkillers (2%), steroids (1%), home remedies (1%)

[5] 719 is the government hotline for COVID-19 reporting and information-seeking

[6] Eat well: eat healthy, eat vegetables, etc

[7] Live well: exercise, enough rest, fresh air, don't panic, avoid stress, etc

[8] Other advice: don't touch face, avoid handshakes, do not take any medicines, don't reuse mask, etc

[9] Things that were deemed particularly helpful or nice related mainly to: reassurance that infection risk is low if prevention measures are followed; high chance of recovery in case of infection; detailed information on what to do or where to seek further help; and follow-up calls in cases where the provider was busy at the time of the initial call.

[10] Things that were deemed not helpful or not nice related mainly to: calls being hang up; inattentiveness or being in a hurry to finish the call; and dismissiveness (eg, "don't you watch news? The symptoms are public knowledge!" and "we don't have those medicines you are asking for, jaribu kwingine [try elsewhere])

Infection prevention during service provision

The main infection prevention measures during service provision were hand hygiene, face masks, disinfection of services, and physical distancing (**Table 4**). Eighty-five per cent of respondents rated the prevention measures instituted at their pharmacy as either adequate (72%) or very adequate (13%). Only one pharmacy reported that a staff member had ever tested positive for COVID-19, though no pharmacy reported regular testing of staff.

For peer review only

Table 4. COVID-19 infection prevention during service provision by private retail pharmacies in Kenya

Infection prevention measures	Nairobi (N=38)		Mombasa (N=36)		Kisumu (N=34)		Total (N=108)	
	n	%	n	%	n	%	n	%
**Among staff**								
Physical distancing[1]	25	66%	26	72%	27	79%	78	72%
Temperature monitoring	13	34%	14	39%	13	38%	40	37%
Hand hygiene	35	92%	33	92%	32	94%	100	93%
Disinfection of surfaces	29	76%	30	83%	27	79%	86	80%
Face masks[2]	34	89%	30	83%	30	88%	94	87%
Goggles	0	0%	1	3%	1	3%	2	2%
Ingestion of immune boosters	1	3%	0	0%	0	0%	1	1%
**Among clients**								
Physical distancing[1]	33	87%	31	86%	30	88%	94	87%
Temperature monitoring	13	34%	9	25%	11	32%	33	31%
Hand hygiene[3]	36	95%	33	92%	30	88%	99	92%
Face masks	27	71%	32	89%	27	79%	86	80%
Non-touch payment methods[4]	14	37%	16	44%	10	29%	40	37%
Information materials and messages[5]	12	32%	13	36%	11	32%	36	33%

[1] Physical distancing was achieved through various means including: reducing number of staff and working in shifts, ensuring a distance of 1.5 -3 meters between staff and clients through the use of posters and floor/seat markings, serving one client at a time or other crowd control measures, ribbons/ ropes/ glass/ metal-grill/ wooden barriers between client and provider, and home delivery of products. One pharmacy noted that "patients with cough and related symptoms were always first referred to the health center for covid19 testing before being served further".

[2] Majority of pharmacies (95%) were using surgical/medical masks; only 6% reported use of cloth masks.

[3] Hand hygiene measures among clients included: handwashing, hand sanitizer, and "no hand-shakes" policy

[4] Non-touch payment methods included: mobile money, and credit/ debit cards; one pharmacy reported disinfection of cash.

[4] Messages were delivered through: posters (21%), leaflets (5%), television screens (2%), and audio announcements (1%)

Information sources and training

Sources of general COVID-19 information included: continuing professional development sessions (37% of respondents), ministry of health (MoH) communications (23%), social media and internet (19%), television and radio (12%), Kenya Pharmaceutical Association (KPA)/ Pharmaceutical Society of Kenya (PSK) communications (5%), scientific journals (2%) and colleagues (2%). For specific questions about COVID-19, providers consulted the pharmacy-in-charge (48%), nearby doctor (37%), MoH officials (32%), the internet (5%), and medical representatives (2%).

Interviewees felt that the official announcement of the first case played a major role in awareness creation, though some reported having learnt about the outbreak prior to that, through radio, television, and social media. However, providers decried the rapidly changing and sometimes contradictory information, for example *"on whether to use or not to use masks"*.

For peer review only - http://bmjopen.bmj.com/site/about/guidelines.xhtml

Fifty-two (48%) pharmacies had at least one staff member trained on COVID-19, with a median [range] of 1 [1-8] providers trained. This did not vary by county or by membership in professional associations. Modalities of training included: on-site in the pharmacy (67% of respondents), virtual (54%), and off-site (13%). Virtual trainings were more prevalent in Kisumu compared to Nairobi (76% vs 35%, p=0.04). Training sessions were facilitated by MoH (73%), KPA (51%), PSK (19%), AMREF (9%), and Kenyatta National Hospital (5%). While some interview participants felt they had received "a lot'' of training, others felt the trainings were not very adequate, particularly with regard to inclusion of updated information in follow-up sessions and the fact that most trainings included only the pharmacy in-charge and not the entire staff.

External support and linkages

Interviewees expressed appreciation for guidance from the ministry of health, including the pharmacy and poisons board, who "*did some inspection and brought some banners [information materials] on how I can protect myself and the general public."* The government-led community awareness-creation activities were also hailed as an important complement to pharmacy efforts in educating the public.

However, examples of direct linkages and material support were rare. One interviewee in Nairobi reported that community health workers from a nearby health centre regularly visited the pharmacy to collect information on number of suspect cases seen. Another interviewee in Kisumu described an instance where an ambulance was sent promptly after he reported a patient in critical condition.

More commonly, a feeling of isolation and neglect prevailed, with pharmacies reporting that they were not receiving much support from the government or other external parties with regard to COVID-19, unlike what happens with other public health programs:

> *"So far being a private facility, we are not receiving any financial support from the organizations in COVID related issues. We don't receive that apart from PS Kenya [NGO supporting TB and HIV programs]."*
>
> - Male pharmaceutical technologist, Kisumu, 301

Confidence, motivation, and coping mechanisms

Majority (87%) of questionnaire respondents reported feeling confident (68%) or very confident (19%) about providing COVID-19-related services. Over half felt prepared (51%) or very prepared (7%) about handling a client who presents to the pharmacy with COVID-19 symptoms.

Motivation for supporting delivery of COVID-19 services included: "to help people protect themselves or prevent transmission (75%), "It's part of my job/ I am required to do it" (14%), encouragement and support from professional associations (7%), "to boost the profile of the pharmacy" (2%), and "to make money" (1%). When asked how much they felt their personal role

For peer review only - http://bmjopen.bmj.com/site/about/guidelines.xhtml

in the COVID-19 response was valued by the community, the majority stated highly valued (68%) or very highly valued (11%)

In addition to the operational adjustments described above, interviewees described other coping mechanisms such putting up a shade outside the pharmacy to prevent crowding inside and improvisation of hand sanitizers when not available from suppliers. Some interviewees also reported going the extra mile sometimes, eg, by providing free masks to clients.

Provider suggestions and recommendations
Seventy-six (70%) questionnaire respondents highlighted contributions that they felt pharmacies can uniquely make to the national response, including: provision of information and counselling to clients (64%), distribution of preventive materials (21%), triage and referral of suspect cases (17%), surveillance of infection rates and community adherence to preventive measures (14%), provision of preventive and curative therapies (12%), and maintenance of long-term therapy (3%). Pharmacies' location within communities was cited as a strength when it comes to surveillance of client behaviour and community awareness creation.

To facilitate these inputs, pharmacies suggested additional support that they would require from the government and other external parties, including: training (34% of respondents), free or subsidized preventive materials such as masks, sanitizers, and thermo guns for own use and distribution to clients (34%), supply management such as price and quality controls (15%), clear guidelines and referral systems (9%), community awareness creation (7%), information materials (6%), financial support such as loan repayment holidays and medical insurance (6%), and testing services (5%).

These suggestions were echoed in interviews. Post-marketing surveillance to ensure quality of products in the market and a streamlined process to capture and transmit information back to the government were pinpointed as key gaps in the pandemic response. Overall, there were earnest calls for more deliberate engagement, oversight, and support supervision by the government and other implementing partners:

> *"If the government could put us, the pharmacists and pharm techs in the you know, treat us like people who are fighting this virus and facilitate this thing it would be a major shift, it would be a major shift."*
>
> - Male pharmacy in charge, Mombasa, 205

Discussion
In this study among private retail pharmacies (community pharmacies) in Kenya, we describe the impact of the COVID-19 pandemic on operations, COVID-19-related products and services being provided, infection prevention during service provision, and provider suggestions and recommendations on how to improve pandemic preparedness and response.

For peer review only - http://bmjopen.bmj.com/site/about/guidelines.xhtml

The initial weeks after the pandemic reached Kenya were characterized by fear and panic among providers and a surge in client flow. Broadly speaking, the experiences documented in our study can be captured with the apt words of Austin et al in their paper describing the experiences of community pharmacists in Ontario, Canada, viz: “the scale and magnitude of the COVID-19 pandemic defied imagination or pre-planning, yet health care professionals like pharmacists were required to continue to provide services and care to patients – and in many cases, expand their repertoire of clinical skills to assume new and even more challenging responsibilities” [15].

The initial surge in demand waned rapidly, with client flow dipping below pre-pandemic levels within a few weeks and remaining depressed until the time of data collection, seven months later. This was surprising as we had hypothesized, before the study, that pharmacies would experience an overall increase in demand as patients redirected their care-seeking from health facilities for fear of exposure to infection and quarantine. Participants attributed the decrease in demand mainly to the movement restrictions instituted by government. However, they also suggested – ironically – that clients, especially those with flu-like symptoms, may have avoided pharmacies for fear of being referred to public facilities and subsequently quarantined.

Our findings clearly show that community pharmacies in Kenya were undertaking potentially impactful activities to support the response to the COVID-19 pandemic. Despite a feeling of disconnection from the government-led national response, providers expressed great readiness and motivation for provision of COVID-19-related services, similar to providers in Qatar [30], the USA [31], and other settings around the world [32]. The services pharmacies in our study were providing are generally similar to those suggested by experts in the field [22, 33-35], as well as those being provided in other low-to-middle income countries [36-39] and in high-income settings [30, 40, 41].

However, management of drug shortages and prevention of stockpiling (hoarding) by clients [22, 30, 33] were reported only as challenges and not as roles that Kenyan pharmacies were playing or could play. Additionally, while 60% of questionnaire respondents said they were receiving queries and providing information and counseling over the phone, other remote services such as online prescribing and home delivery of medications [22, 36] were not being widely used or mentioned as potential additional roles. Participants in our study, being frontline workers, may have deemed the carrying out of these innovations as being beyond the sphere of their influence. Similarly, COVID-19 vaccine administration was not mentioned as a potential role, understandably perhaps because a vaccine was not available at the time. But pharmacies in Kenya are actively involved in provision of other vaccines [42, 43], hence it is reasonable to hypothesize that COVID-19 vaccines could be delivered through private pharmacies in the future. Interviews with policymakers and captains of industry may be required to probe these topics further.

For peer review only - http://bmjopen.bmj.com/site/about/guidelines.xhtml

The finding that no pharmacy was providing COVID-19 testing was unsurprising since no approved point-of-care (POC) tests were available at the time of the study. The more interesting finding was that significant demand and support for pharmacy-based testing existed, similar to what studies in the Middle East [44] and the USA [31] have found. In this context and given the fact that at least three rapid POC tests have since been approved in Kenya [45], there is an urgent need to start developing policies to guide implementation. This will obviate the emergence of an uncoordinated and potentially dangerous service, like what we saw in 2016 with HIV testing before policy guidelines were issued the following year [46, 47].

For treatment of COVID-19 symptoms, most pharmacies recommended alternative therapies and nutritional supplements such as vitamin C and zinc, and only about a third suggested conventional therapies such as antibiotics which have no proven efficacy [13, 14]. This finding is similar to that of a small study in Nairobi in May 2020 which found that despite increased requests for antimalarials and antibiotics, pharmacists recommended alternative therapies [48]. A similar finding was also obtained in a multinational survey of Asian pharmacists [35]. While alternative therapies and nutritional supplements are themselves not evidence-based (as no rigorous trials have proven their efficacy against SARS-CoV-2), they have a better safety profile and their useful effects are more biologically plausible since they have been used for ages against the flu virus, itself a coronavirus. These findings are therefore encouraging. However, among Egyptian pharmacies, the antibiotic azithromycin was given to about 40% of presumptive COVID-19 patients with mild to moderate symptoms, albeit based on physician recommendation [49]. Further research in different jurisdictions may be needed to describe and contrast the use of antibiotics and other unproven COVID-19 therapies, in order to inform the promotion of rational drug use and the prevention of antimicrobial resistance.

Our study identified other important gaps in community pharmacies' preparedness and response to the pandemic. Notably, pharmacies did not seem to have any well-defined operational linkages to other units of the healthcare system, including for example, the emergency operations center at the ministry of health. Besides, while only about half of pharmacies had at least one staff member trained on COVID-19, some of those trained felt they had received a lot of training, suggesting a "network effect" where some providers were receiving many trainings while others received none.

Specific programs will be required to address these gaps and enhance pandemic preparedness. Templates for public-private cooperation in pandemic responses do exist from other settings. Ung *et al*, for example, report how in one administrative region on the south-east coast of China, pre-existing public-private partnerships between the local government and community pharmacies ensured rapid provision and prevented hoarding of face masks through a government-led "Guaranteed Mask Supply for Macao Residents Scheme" [50]. A qualitative study in Canada, identified key features that predicted community pharmacists' resilience during the early period of the pandemic, including: use of and comfort with technology; early adoption

For peer review only - http://bmjopen.bmj.com/site/about/guidelines.xhtml

of corporate and professional guidance; emphasis on task-focus rather than multi-tasking; and provision of personal protective equipment [15]. Roll-out of pharmacy-based COVID-19 programs should be accompanied with implementation studies to better uncover aspects of capacity, preparedness, and resilience.

Our study had a number of strengths. First, we took advantage of information collected in a recent study to speed up recruitment and data collection. Second, we used multiple quantitative and qualitative methods to enhance the comprehensiveness and reliability of the findings. Of note, we used simulated clients to objectively assess provider practices, an approach that has been shown to be superior to methods that rely on real patients, such as exit interviews, chart abstraction, and direct observation [51]; and is gaining acceptance as a gold standard for the measurement of clinical practice quality around the world [52]. Our study also had some weaknesses. We observed a significant variation in detection rate by individual fieldworkers, suggesting variation in the way different fieldworkers enacted the simulation. However, detection rate did not vary across counties (perhaps because fieldworkers were scheduled across counties) and outcomes did not vary by fieldworker, hence this bias is unlikely to have had an effect on our comparisons across counties. Another weakness of our study was the somewhat low response rate (55%), considering that we targeted pharmacies that had participated in a similar study less than a year previously. Compared to non-participating pharmacies, participating pharmacies had features suggestive of higher operational capacity and interest in public health interventions. While this limits generalizability of our findings, it can also be looked at in positive light. We propose that pharmacies with these characteristics could be identified for inclusion in a "practice and learning network" that could provide a platform for testing implementation strategies for various public health interventions [53-55]. Scale up of such interventions would then target similar pharmacies and include strategies for enhancing operational capacity and interest in public health among the other pharmacies.

In conclusion, we found that private retail pharmacies in Kenya were actively contributing to the COVID-19 response despite an apparent disconnection from the national response program. More deliberate engagement, support and linkages are required. Notably, there is an urgent need to develop policy guidelines for pharmacy-based COVID-19 testing, a service that is clearly needed and which could greatly increase test coverage. Roll-out of this and other pharmacy-based COVID-19 programs should be accompanied with implementation research in order to inform current and future pandemic responses.

Acknowledgments

For peer review only - http://bmjopen.bmj.com/site/about/guidelines.xhtml

We acknowledge the contributions of all participating pharmacy service providers. We thank James Wafula for supporting database development and data management; and Langat Justus, Lucy Omumbo, Milkah Auka, and Rodgers Ayoma for supporting the simulated client survey.

Contributors

Conceived the study: PM EB. Developed protocol: PM DM SM EB. Performed the experiments: PM AM; Analysed the data: PM AM JN. Wrote the manuscript: PM AM DM JN SM EB. Approved the final manuscript: PM AM DM JN SM EB.

Competing interests

The authors declare not conflicts of interest.

Funding

This work was supported by Wellcome Trust [#211353/Z/18/Z]. For the purpose of open access, the author has applied a CC-BY public copyright licence to any author accepted manuscript version arising from this submission. The KWTRP at the Centre for Geographical Medicine Research-Nairobi is supported by core funding from the Wellcome Trust [#077092]. This report was published with the permission of Director KEMRI CGMRC.

Data availability statement

Underlying data can be accessed in the Harvard Dataverse using the following link: https://doi.org/10.7910/DVN/XTLUFP

References

1. *Zhu N, Zhang D, Wang W, Li X, Yang B, Song J, et al.* A novel coronavirus from patients with pneumonia in China, 2019. *New England Journal of Medicine* 2020.
2. *Anand U, Cabreros C, Mal J, Ballesteros Jr F, Sillanpää M, Tripathi V, et al.* Novel coronavirus disease 2019 (COVID-19) pandemic: From transmission to control with an interdisciplinary vision. *Environmental research* 2021:111126.
3. Coronavirus cases and deaths. https://www.worldometers.info/coronavirus/ Updated, Date Accessed: 29/09.
4. *Lai S, Ruktanonchai NW, Zhou L, Prosper O, Luo W, Floyd JR, et al.* Effect of non-pharmaceutical interventions for containing the COVID-19 outbreak: an observational and modelling study. *medRxiv* 2020.
5. *Cowling BJ, Ali ST, Ng TW, Tsang TK, Li JC, Fong MW, et al.* Impact assessment of non-pharmaceutical interventions against coronavirus disease 2019 and influenza in Hong Kong: an observational study. *The Lancet Public Health* 2020.
6. *Fauci AS.* The story behind COVID-19 vaccines: American Association for the Advancement of Science, 2021.
7. *Kashte S, Gulbake A, El-Amin III SF, Gupta A.* COVID-19 vaccines: rapid development, implications, challenges and future prospects. *Human cell* 2021:1-23.
8. *US National Institutes of Health.* Care of Critically Ill Adult Patients With COVID-19. 2020. https://www.covid19treatmentguidelines.nih.gov/critical-care/

For peer review only - http://bmjopen.bmj.com/site/about/guidelines.xhtml

9. *US National Institutes of Health*. Outpatient Management of Acute COVID-19. 2021. https://www.covid19treatmentguidelines.nih.gov/outpatient-management/
10. *Jin Y, Yang H, Ji W, Wu W, Chen S, Zhang W, et al.* Virology, epidemiology, pathogenesis, and control of COVID-19. *Viruses* 2020;12(4):372.
11. *Siemieniuk RA, Bartoszko JJ, Ge L, Zeraatkar D, Izcovich A, Kum E, et al.* Drug treatments for covid-19: living systematic review and network meta-analysis. *BMJ* 2020;370:m2980.
12. *American Society of Hospital Pharmacists*. Assessment of Evidence for COVID-19-Related Treatments. 2021. https://www.ashp.org/-/media/assets/pharmacy-practice/resource-centers/Coronavirus/docs/ASHP-COVID-19-Evidence-Table.ashx?la=en&hash=FD5D4C4AEDAC4B725B85D3375A88216E526C39EC
13. *World Health Organization*. Clinical management of COVID-19. 2020.
14. *US National Institutes of Health*. Prevention and Prophylaxis of SARS-CoV-2 Infection. 2021. https://www.covid19treatmentguidelines.nih.gov/overview/prevention-of-sars-cov-2/
15. *Austin Z, Gregory P*. Resilience in the time of pandemic: the experience of community pharmacists during COVID-19. *Research in Social and Administrative Pharmacy* 2021;17(1):1867-75.
16. *Abuya TO, Mutemi W, Karisa B, Ochola SA, Fegan G, Marsh V*. Use of over-the-counter malaria medicines in children and adults in three districts in Kenya: implications for private medicine retailer interventions. *Malar J* 2007;6:57.
17. *Bigogo G, Audi A, Aura B, Aol G, Breiman RF, Feikin DR*. Health-seeking patterns among participants of population-based morbidity surveillance in rural western Kenya: implications for calculating disease rates. *Int J Infect Dis* 2010;14(11):e967-73.
18. *Tran BX, Vu GT, Le HT, Pham HQ, Phan HT, Latkin CA, et al.* Understanding health seeking behaviors to inform COVID-19 surveillance and detection in resource-scarce settings. *Journal of Global Health* 2020;10(2).
19. *Fonck K, Mwai C, Rakwar J, Kirui P, Ndinya-Achola JO, Temmerman M*. Healthcare-seeking behavior and sexual behavior of patients with sexually transmitted diseases in Nairobi, Kenya. *Sex Transm Dis* 2001;28(7):367-71.
20. *Onwujekwe O, Hanson K, Uzochukwu B*. Do poor people use poor quality providers? Evidence from the treatment of presumptive malaria in Nigeria. *Trop Med Int Health* 2011;16(9):1087-98.
21. *International Pharmaceutical Federation*. Guidelines for Pharmacists and the Pharmacy Workforce to manage COVID-19. 2020. https://www.fip.org/publications?publicationCategory=97&publicationYear=2020&publicationKeyword=
22. *Aruru M, Truong H-A, Clark S*. Pharmacy Emergency Preparedness and Response (PEPR): a proposed framework for expanding pharmacy professionals' roles and contributions to emergency preparedness and response during the COVID-19 pandemic and beyond. *Research in Social and Administrative Pharmacy* 2021;17(1):1967-77.
23. *Shaw, N*. Kenya: protocols for the containment of the coronavirus (COVID-19) announced. 2020. https://www.ensafrica.com/news/detail/2373/kenya-announces-protocols-for-the-containment
24. *Worldometer*. Kenya Coronavirus Cases. 2021. https://www.worldometers.info/coronavirus/country/kenya/
25. *Wangari EN, Gichuki P, Abuor AA, Wambui J, Okeyo SO, Oyatsi HT, et al.* Kenya's response to the COVID-19 pandemic: a balance between minimising morbidity and adverse economic impact. *AAS Open Research* 2021;4.
26. *Barasa E, Kazungu J, Orangi S, Kabia E, Ogero M, Kasera K*. Assessing the Indirect Health Effects of the COVID-19 Pandemic in Kenya. *CGD Work. Pap* 2021;570.

For peer review only

27. *Global Alliance for Vaccines and Immunization*. Kenya completes its first round of COVID-19 vaccinations. 2021. https://www.gavi.org/vaccineswork/kenya-completes-its-first-round-covid-19-vaccinations
28. *Pharmacy and Poisons Board*. Online Services Portal: License Status. 2019. https://practice.pharmacyboardkenya.org/LicenseStatus
29. *Statista*. Cumulative number of confirmed coronavirus (COVID-19) cases in Kenya by county. 2021. https://www.statista.com/statistics/1136519/cumulative-coronavirus-cases-in-kenya-by-county/
30. *ElGeed H, Owusu Y, Abdulrhim S, Awaisu A, Kattezhathu VS, Abdulrouf PV, et al.* Evidence of community pharmacists' response preparedness during COVID-19 public health crisis: A cross-sectional study. *The Journal of Infection in Developing Countries* 2021;15(01):40-50.
31. *Nguyen E, Owens CT, Daniels T, Boyle J, Robinson RF*. Pharmacists' Willingness to Provide Coronavirus Disease (COVID-19) Services and the Needs to Support COVID-19 Testing, Management, and Prevention. *Journal of community health* 2020:1-6.
32. *Jordan D, Guiu-Segura JM, Sousa-Pinto G, Wang L-N*. How COVID-19 has impacted the role of pharmacists around the world. *Farmacia Hospitalaria: Organo Oficial de Expresion Cientifica de la Sociedad Espanola de Farmacia Hospitalaria* 2021;45(2):89-95.
33. *Liu S, Luo P, Tang M, Hu Q, Polidoro JP, Sun S, et al.* Providing pharmacy services during the coronavirus pandemic. *International Journal of Clinical Pharmacy* 2020;42(2):299-304.
34. *Bukhari N, Rasheed H, Nayyer B, Babar Z-U-D*. Pharmacists at the frontline beating the COVID-19 pandemic: Springer, 2020.
35. *Godman B, Haque M, Islam S, Iqbal S, Urmi UL, Kamal ZM, et al.* Rapid assessment of price instability and paucity of medicines and protection for COVID-19 across Asia: findings and public health implications for the future. *Frontiers in public health* 2020;8.
36. *Bahlol M, Dewey RS*. Pandemic preparedness of community pharmacies for COVID-19. *Research in Social & Administrative Pharmacy* 2021;17(1):1888.
37. *Meghana A, Aparna Y, Chandra SM, Sanjeev S*. Emergency preparedness and response (EP&R) by pharmacy professionals in India: lessons from the COVID-19 pandemic and the way forward. *Research in Social and Administrative Pharmacy* 2021;17(1):2018-22.
38. *Kasahun GG, Kahsay GM, Asayehegn AT, Demoz GT, Desta DM, Gebretekle GB*. Pharmacy preparedness and response for the prevention and control of coronavirus disease (COVID-19) in Aksum, Ethiopia; a qualitative exploration. *BMC health services research* 2020;20(1):1-6.
39. *Hoti K, Jakupi A, Hetemi D, Raka D, Hughes J, Desselle S*. Provision of community pharmacy services during COVID-19 pandemic: a cross sectional study of community pharmacists' experiences with preventative measures and sources of information. *International journal of clinical pharmacy* 2020;42(4):1197-206.
40. *Zaidi STR, Hasan SS*. Personal protective practices and pharmacy services delivery by community pharmacists during COVID-19 pandemic: Results from a national survey. *Research in Social and Administrative Pharmacy* 2021;17(1):1832-37.
41. *Giua C, Paoletti G, Minerba L, Malipiero G, Melone G, Heffler E, et al.* Community pharmacist's professional adaptation amid Covid-19 emergency: a national survey on Italian pharmacists. *International journal of clinical pharmacy* 2021:1-8.
42. *Goodlife Pharmacy*. Get your vaccinations today. 2021. https://www.goodlife.co.ke/get-your-vaccinations-today/
43. *Portal Pharmacy*. Flu vaccine. 2021. https://portalpharmacy.ke/flu-vaccine

For peer review only - http://bmjopen.bmj.com/site/about/guidelines.xhtml

44. *Mukattash TL, Jarab AS, Abu-Farha RK, Nusair M, Mukattash IL, Obaidat RM, et al.* Willingness and readiness to test for COVID-19; A qualitative exploration of community pharmacists. *International journal of clinical practice* 2020;74(12):e13620.
45. *Pharmacy and Poisons Board, Kenya Ministry of Health*. View Medical Devices. 2021. https://products.pharmacyboardkenya.org/ppb_admin/pages/system_reports_public.php
46. *Mugo PM, Micheni M, Shangala J, Hussein MH, Graham SM, Rinke de Wit TF, et al.* Uptake and Acceptability of Oral HIV Self-Testing among Community Pharmacy Clients in Kenya: A Feasibility Study. *PLoS One* 2017;12(1):e0170868.
47. *National AIDS and STI Control Programme (NASCOP)*. An operational manual for the delivery of HIV Self-Testing services in Kenya. 2017.
48. *Opanga SA, Rizvi N, Wamaitha A, Sefah IA, Godman B*. Availability of medicines in community pharmacy to manage patients with COVID-19 in Kenya; pilot study and implications. *Scholars Academic Journal of Pharmacy* 2021;10(3):36-43.
49. *Elsayed AA, Darwish SF, Zewail MB, Mohammed M, Saeed H, Rabea H*. Antibiotic misuse and compliance with infection control measures during COVID-19 pandemic in community pharmacies in Egypt. *International journal of clinical practice* 2021;75(6):e14081.
50. *Ung COL*. Community pharmacist in public health emergencies: Quick to action against the coronavirus 2019-nCoV outbreak. *Research in Social and Administrative Pharmacy* 2020;16(4):583-86.
51. Kwan A, Daniels B, Bergkvist S, Das V, Pai M, Das J. Use of standardised patients for healthcare quality research in low- and middle-income countries. *BMJ Global Health* 2019;4(5).
52. *Daniels B, Dolinger A, Bedoya G, Rogo K, Goicoechea A, Coarasa J, et al.* Use of standardised patients to assess quality of healthcare in Nairobi, Kenya: a pilot, cross-sectional study with international comparisons. *BMJ global health* 2017;2(2):e000333.
53. *English M, Ayieko P, Nyamai R, Were F, Githanga D, Irimu G*. What do we think we are doing? How might a clinical information network be promoting implementation of recommended paediatric care practices in Kenyan hospitals? *Health research policy and systems* 2017;15(1):1-12.
54. *Gachau S, Ayieko P, Gathara D, Mwaniki P, Ogero M, Akech S, et al.* Does audit and feedback improve the adoption of recommended practices? Evidence from a longitudinal observational study of an emerging clinical network in Kenya. *BMJ global health* 2017;2(4):e000468.
55. *Tuti T, Bitok M, Malla L, Paton C, Muinga N, Gathara D, et al.* Improving documentation of clinical care within a clinical information network: an essential initial step in efforts to understand and improve care in Kenyan hospitals. *BMJ Global Health* 2016;1(1):e000028.

For peer review only - http://bmjopen.bmj.com/site/about/guidelines.xhtml

FIGURE TITLES

Figure 1. Challenges experienced by pharmacies during the COVID-19 pandemic in Kenya

For peer review only

For peer review only - http://bmjopen.bmj.com/site/about/guidelines.xhtml

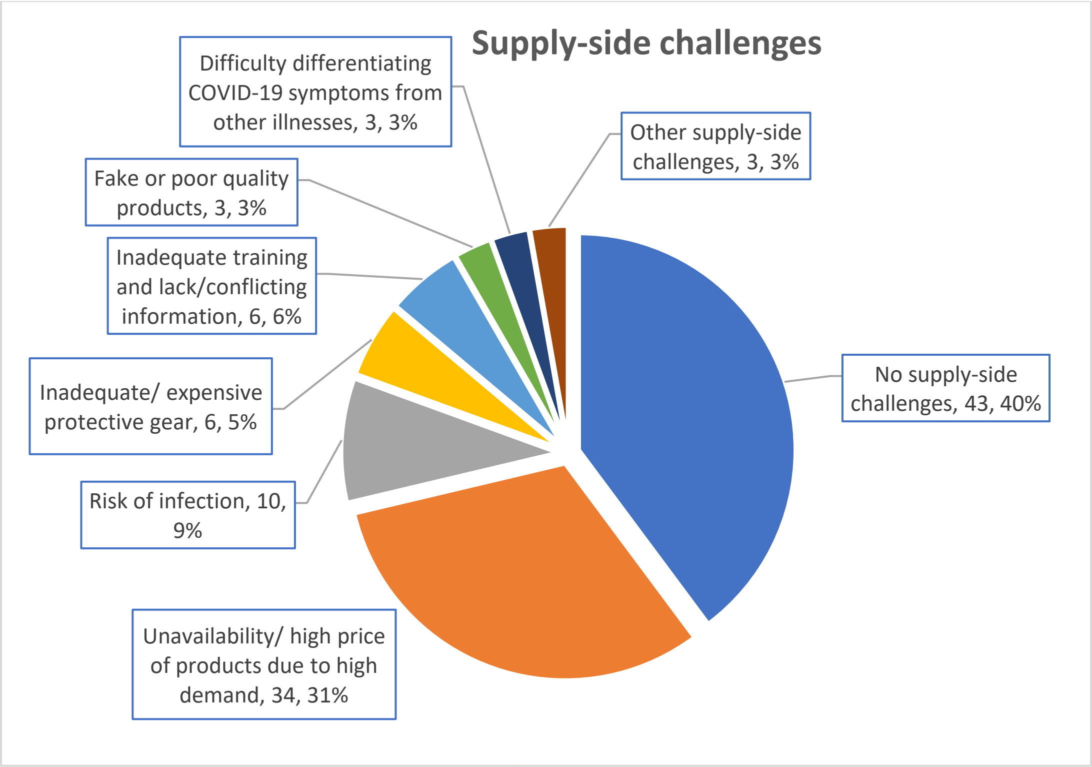

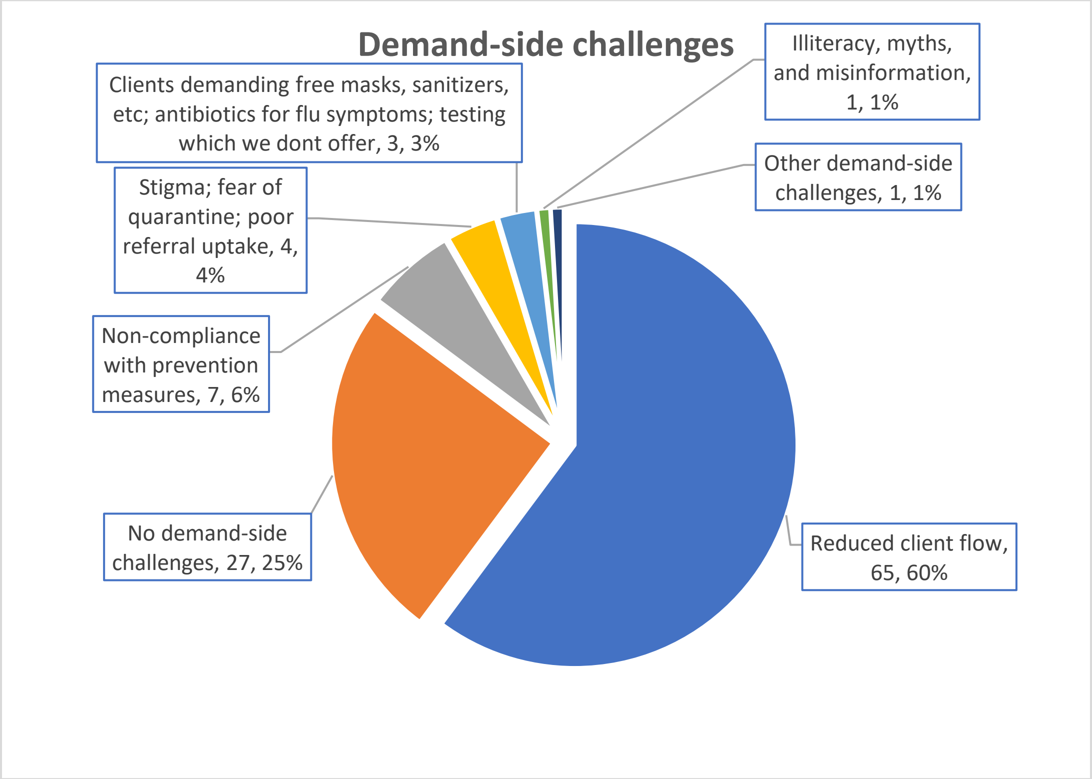

Figure 1. Challenges experienced by pharmacies during the COVID-19 pandemic in Kenya

Supplementary Table I: Characteristics of participating pharmacies and respondents

A. Characteristics of participating pharmacies

Characteristic	Nairobi (n=38)		Mombasa (n=36)		Kisumu (n=34)		Total (n=108) ^	
	n	%	n	%	n	%	n	%
**Setting**								
Urban commercial centre	23	61%	29	81%	12	35%	64	59%
Urban residential area	13	34%	4	11%	6	18%	23	21%
Urban informal settlement	2	5%	3	8%	2	6%	7	6%
Rural town/ shopping centre	0	0%	0	0%	14	41%*	14	13%
**Years of operation**								
Number of years in operation (median [range])	5	1-19	4	1-42	5	1-11	5	1-42
**Physical facilities**								
Total number of rooms (Median [range])	2	1-10	2	1-6	2	1-8	1	1-10
Consultation room	14	37%	10	28%	20	59%*	44	41%
On-site lab	5	13%	1	3%	6	18%	12	11%
**Information systems**								
Computerized stock system	29	81%*	19	53%	23	68%	71	67%
Individual medical records[1]	9	25%	2	6%	7	21%	18	17%
**Chain network membership[2]**								
Part of a chain network	9	24%*	3	8%	1	3%	13	12%
Number of branches nationwide (Median [range])	1	1-41	1	1-41	1	1-9	1	1-41
**Branding[3]**								
None	33	87%	29	81%	32	94%	94	87%
Green Cross	2	5%	2	6%	0	0%	4	4%
Pharmnet (blue cross)	0	0%	3	8%	1	3%	4	4%
Corporate	3	8%	2	6%	1	3%	6	6%
**Distance to the nearest clinic**								
Within the pharmacy	0	0%	0	0%	2	6%	2	2%
< 100 meters	17	45%	13	36%	9	26%	39	36%
< 1km	19	50%	16	44%	21	62%	56	52%
< 5km	2	5%	7	19%	2	6%	11	10%
**Distance to the nearest HIV testing site**								
< 100 meters	14	37%	12	33%	11	32%	37	34%
< 1km	18	47%	16	44%	21	62%	55	51%
< 5km	4	11%	7	19%	2	6%	13	12%
>5km	1	3%	0	0%	0	0%	1	1%
Not sure	1	3%	1	3%	0	0%	2	2%
**Service providers**								
Total number of service providers (Median [range])	3*	1-16	2	1-9	2	1-4	2	1-16
At least one pharmacy degree holder	8	21%	7	19%	3	9%	18	17%

For peer review only

Characteristic	Nairobi (n=38)		Mombasa (n=36)		Kisumu (n=34)		Total (n=108) ^	
	n	%	n	%	n	%	n	%
At least one pharmacy diploma holder	36	95%	35	97%	34	100%	105	97%
All staff belong in a professional association	24	63%	19	53%	18	53%	61	56%
**Management**								
Written job descriptions	21	55%	12	33%	14	41%	47	44%
Regular staff meetings[4]	21	55%	16	44%	19	56%	56	52%
**Opening hours**								
24 hours daily, 7 days a week	4	11%	1	3%	1	3%	6	6%
At least seven days a week	23	61%	28	78%	25	74%	76	70%
At least 6 days a week	38	100%	34	94%	34	100%	106	98%
**Work load**								
Walk-in clients per day (Median [range])	55	15-300	50	20-200	40	20-200	50	15-300
Online clients per day (Median [range])	1	0-20	0	0-20	0	0-10	0	0-20
**Health promotion services**								
Over-the-counter STI treatment	29	76%	29	81%	30	88%	88	81%
Weight management	12	32%	6	17%	10	29%	28	26%
Smoking cessation	4	11%	1	3%	1	3%	6	6%
Vaccination	0	0%	1	3%	3	9%	4	4%
**Screening services**								
Blood pressure measurement	26	68%	26	72%	25	74%	77	71%
Blood sugar testing	19	50%	25	69%	20	59%	64	59%
HIV self-testing	21	55%	22	61%	20	59%	63	58%
Malaria testing	9	24%	19	53%	27	79%*	55	51%
In-pharmacy pregnancy testing	9	24%	9	25%	18	53%*	36	33%
Other screening services[5]	1	3%	0	0%	5	15%*	6	6%

^ Of 195 target pharmacies that had participated in the previous PHP study, 108 (55%) participated in the current survey. Of 87 that did not participate: 55 (63%) failed to complete the questionnaire after three reminders, 15 (17%) reported that the initial respondent had left and no one else was willing to participate, 8 (9%) were unreachable, 3 (3%) reported that the management did not approve participation, 2 (1%) were undergoing transition, 1 (1%) was closed, and 3 (3%) had other reasons (no access to smartphone or computer, preferred face-to-face interview, sold the pharmacy).

** Indicates significant difference between counties (p<0.05), based on chi square test or analysis of variance for means*

[1] An individual medical record is a document detailing the medications an individual patient is on, indications and drug allergies; may also include other clinical information.

[2] Chain network was defined as 6 or more branches nationwide, derived from chain size distribution pattern; 79 (73%) pharmacies were single branch, 16 (15%) were part of a 2-5 branch network, and 13 (12%) were part of a chain network

[3] Green Cross is a professional network of pharmacies owned by pharmacists; Pharmnet is for pharmaceutical technologists

[4] Excludes 18 pharmacies that responded "not applicable" since they have only one staff member

[5] Other screening services included; typhoid testing (n=4) and tuberculosis screening (n=2)

STI: Sexually transmitted infection

For peer review only

B. Characteristics of respondents

Characteristic	Nairobi (N=38)		Mombasa (N=36)		Kisumu (N=34)		Total (N=108)	
	n	%	n	%	n	%	n	%
**Gender**								
Female	12	32%	13	36%	16	47%	41	38%
Male	26	68%	23	64%	18	53%	67	62%
**Age**								
20 – 29 years	12	32%	12	33%	14	41%	38	35%
30 – 39 years	19	50%	18	50%	16	47%	53	49%
40 – 44 years	5	13%	4	11%	2	6%	11	10%
45 years and over	2	5%	2	6%	2	6%	6	6%
**Designation (role)**								
Owner	9	24%	12	33%	12	35%	33	31%
In-charge/ Superintendent	11	29%	13	36%	8	24%	32	30%
Staff[1]	18	47%	11	31%	14	41%	43	40%
**Highest level of education[2]**								
Non-health qualifications	0	0%	2	6%	1	3%	3	3%
Health-related certificate/ diploma/ bachelor's degree	2	5%	0	0%	2	6%	4	4%
Pharmacy certificate	2	5%	0	0%	1	3%	3	3%
Pharmacy Diploma	29	76%	31	86%	28	82%	88	81%
Pharmacy degree	3	8%	3	8%	0	0%	6	6%
Health-related master's degree	2	5%	0	0%	2	6%	4	4%
**Experience (Median [Range])**								
Total number of years worked	7	1-25	5	2-41	6	2-20	6	1-41
Number of years worked in the current pharmacy	3	1-14	3	1-30	3	1-17	3	1-30

[1] Staff designations included: pharmaceutical technologist (n=35), pharmacist (n=5), pharmacy assistant (n=2), and nurse aid (n=1)
[2] Non-health qualifications included: business management (n=1), supplies management (n=1), and college (n=1); health-related certificate/ diploma/ bachelor's degree included: medicine (n=1), nursing (n=2), and environmental health (n=1)h; health-related master's degree included: pharmacy (n=1), and other (n=3)

For peer review only

Supplementary Table II. Characteristics of in-depth interview participants. The interviewee # is used to reference illustrative quotes in the results section. Rows are sorted by interviewee #.

Interviewee #	County	Practice setting	Sex	Age category (years)	Highest level of education	Years in practice	Position
101	Nairobi	Urban residential area	Female	20 - 24	Pharmacy Diploma	4	Staff
102	Nairobi	Urban commercial center	Male	30 - 34	Pharmacy Diploma	10	Superintendent
103	Nairobi	Urban commercial center	Male	25 - 29	Pharmacy degree	2	Staff
104	Nairobi	Urban commercial center	Female	30 - 34	pharmacy assistant certificate	8	Staff
105	Nairobi	Urban commercial center	Male	25 - 29	Pharmacy Diploma	6	Superintendent
106	Nairobi	Urban residential area	Male	35 - 39	Pharmacy Diploma	10	Superintendent
201	Mombasa	Urban commercial center	Female	25 - 29	Pharmacy Diploma	5	In-charge
202	Mombasa	Urban commercial center	Male	40 - 44	Pharmacy Diploma	10	Owner or director
203	Mombasa	Urban informal settlement	Male	35 - 39	BSc supply chain	5	Owner or director
204	Mombasa	Urban commercial center	Male	25 - 29	Pharmacy Diploma	4	Staff
205	Mombasa	Urban commercial center	Male	25 - 29	Pharmacy Diploma	4	Superintendent
206	Mombasa	Urban commercial center	Female	20 - 24	Pharmacy Diploma	2	Staff
301	Kisumu	Urban commercial center	Male	25 - 29	Pharmacy Diploma	2	Staff
302	Kisumu	Rural town/ shopping center	Female	40 - 44	Nursing certificate	17	Staff
303	Kisumu	Urban commercial center	Male	30 - 34	Pharmacy Diploma	7	Superintendent
304	Kisumu	Rural town/ shopping center	Female	35 - 39	Pharmacy Diploma	7	Superintendent
305	Kisumu	Rural town/ shopping center	Female	30 - 34	Health-related masters	9	Owner or director
306	Kisumu	Rural town/ shopping center	Male	35 - 39	Health-related masters	10	Owner or director

For peer review only

<u>**Supplementary file A – PHARMACY QUESTIONNAIRE**</u>

Note to interviewer: This questionnaire is targeted at the pharmacy service provider (PSP) previously included in the PHP study. In case they are not available, target the in-charge or most experienced or most highly-educated PSP, in that order.

BASIC INFORMATION
Interviewer identifier; Pharmacy identifier; Date; Time

INTRODUCTION
Thank you for finding time for this interview. I would like to ask you some questions regarding general operations in your pharmacy and your experience providing COVID19-related services…

<u>**SECTION I: RESPONDENT CHARACTERISTICS**</u>

1. What is your gender? Male/Female/other, specify
2. What is your age category in years?
    - 18 – 24
    - 25 – 29
    - 30 – 34
    - 35 – 39
    - 40 – 44
    - 45 – 49
    - 50 – 54
    - 55 – 59
    - >59

3. What position do you hold in the pharmacy? [pharmacy owner or director/ Superintendent/ Pharmacy in-charge/ Pharm Tech/ Pharmacist/ other, specify]
4. What is your highest level of education? Pharmacy degree/ Pharmacy Diploma / Other
5. How many years have you worked in the retail/ community pharmacy sector? [numeric; if less than a year, enter 0.5]
    a. How many years have you worked in the current pharmacy?

<u>**SECTION II: OVERALL EFFECT OF THE COVID19 EPIDEMIC ON PHARMACY OPERATIONS**</u>

6. Have you had to make any changes to your operations since the outbreak of COVID19 epidemic in Kenya (mid-March 2020)? Yes/no
    a. Have you had to change your hours of operation? [can select more than one]
        - No
        - Reduced open hours per day, specify number of hours reduced
        - Reduced open days per week, specify number of days reduced
        - Closed for some time then re-opened, specify closure period in weeks
        - Closed indefinitely, specify date of closure
        - Other, specify
    b. Have you had to change number of staff? [select one]
        - No
        - Reduced
        - Increased
        - Other, specify
    c. Have you had to change stocking levels? [can select more than one]
        - No
        - Reduced stock volumes [specify 3 main items reduced in volume]
        - Increased stock volumes [specify 3 main items increased in volume]

For peer review only - http://bmjopen.bmj.com/site/about/guidelines.xhtml

d. Have you had to change your meeting routine? Yes/ no
   If yes, describe [open-ended]

7. Have you observed any changes in client flow due to the COVID19 outbreak? [select one]
   No
   Decreased client flow overall
   Increased client flow overall
   Both decrease and increase in different client categories

   a. If both decrease and increase,
      i. Which client category(ies) have decreased? [open-ended]
8. Which client category(ies) have increased? [open-ended]

SECTION III: PROVISION OF COVID19-RELATED SERVICES AND PRODUCTS

9. Which COVID-related equipment and materials does your pharmacy provide? [indicate number of clients in the last one week]
   None
   Cloth masks [number clients]
   Medical masks [number clients]
   N95/ K95 masks/ respirators [number clients]
   Goggles [number clients]
   Face shields [number clients]
   Gowns/ coveralls [number clients]
   Hand sanitizer [number clients]
   Thermometers [number clients]
   Pulse oximeters [number clients]
   Other, specify type and number of clients
   Specify other COVID19-related equipment and materials that your pharmacy provides

10. Which COVID-related preventive and curative therapies does your pharmacy provide? [indicate number of clients in the last one week]
   None
   Vitamin C [number clients]
   Multivitamins [number clients]
   Traditional/ alternative/ natural remedies, specify [number clients]
   Hydroxychloroquine/ chloroquine [number clients]
   Antibiotics, specify [number clients]
   Antivirals, specify [number clients]
   Immunomodulators, specify [number clients]
   Steroids, specify [number clients]
   Other, specify type [number of clients]
   Specify other preventive and curative therapies provided

   Indicate **number of clients** who bought or requested vitamin C in the last one week
      How many of these vitamin C purchases do you think were COVID-related? [numeric]

   [same format for related subsequent questions]

11. Which COVID-related information and counselling do clients seek from your pharmacy? [indicate number of clients in the last one week]
   None

For peer review only - http://bmjopen.bmj.com/site/about/guidelines.xhtml

How to protect themselves from infection [number clients]
Symptoms to look out for if they suspect infection [number clients]
What to do if they develop symptoms or suspect infection [number clients]
Mask use and related problems [number clients]
Counselling for anxiety related to getting infected/ becoming sick with COVID19 [number clients]
Counselling for anxiety about job or business loss [number clients]
Other, specify [number clients]

Specify other type of information or counselling that clients seek: ___________________________

Indicate number of clients in the last one week who asked how to protect themselves from infection

Indicate number of clients in the last one week who asked about symptoms to look out for if they suspect infection

Indicate number of clients in the last one week who asked what to do if they develop symptoms or suspect infection

Indicate number of clients in the last one week who asked about mask use and related problems

Indicate number of clients in the last one week who sought counselling for anxiety related to getting infected or becoming sick with COVID19

Indicate number of clients in the last one week who sought counselling for anxiety about job or business loss

Indicate number of clients in the last one week who sought for other type of information or counselling

12. Select the modes of communication that clients use to seek information/ counselling
    In-person (visit the pharmacy physically)
    Phone calls (including WhatsApp calls etc)
    Texts (including WhatsApp)
    Facebook/ other web-based platforms
    Other, specify

13. What records do you keep regarding COVID-related services and products? [open-ended]
14. Do you prepare any reports regarding COVID-related services and products? Yes/no
    a. To whom do you send the reports?
        Don't send (reports are for internal use)
        Ministry of health (MoH)/ government agency
        Other, specify
    b. What type of information is contained in the reports? [open-ended]

15. What challenges have you had in providing COVID-related services and products? [open-ended]
16. What would you say is your main motivation for supporting delivery of COVID-related services and products? [select one]
        Help people protect themselves/ Help prevent transmission
        Boost the profile of the pharmacy
        Make money
        It's part of my job/ I am required to do it
        Encouragement/ support from professional association (PSK/ KPA/ etc)
        Other, specify
17. How confident do you feel about providing COVID-related services and products? [select one]

For peer review only - http://bmjopen.bmj.com/site/about/guidelines.xhtml
For peer review only

Very uncertain/ uncertain/ confident/ very confident

18. What additional support do you feel pharmacies require in provision of COVID-related services and products? [open-ended]
19. If you don't provide COVID-related products or services,
    a. Do you get clients who ask for COVID-related services or products? Yes/no
        i. If yes, what help do you offer them? Referral/ other
            1. If you refer, to where? Another pharmacy/ clinic/ Hospital/ Lab/ other
    b. What COVID-related services or products do clients ask for?
        Personal protective equipment and materials
        Preventive and curative therapies
        Information/ counselling on COVID19
        Other, specify
    c. Select the type of information/ counselling that clients seek, and indicate number of clients in the last one week [select one]
        How to protect themselves from infection [number clients]
        Symptoms to look out for if they suspect infection [number clients]
        What to do if they develop symptoms/ suspect infection [number clients]
        Mask use and related problems
        Counselling for anxiety related to getting infected/ becoming sick with COVID19 [number clients]
        Counselling for anxiety about job/ business loss [number clients]
        Other, specify [number clients]
    d. Select the modes of communication that clients use to seek information, and indicate number of clients in the last one week [select one]
        In-person (visit the pharmacy physically)
        Phone calls (including WhatsApp calls etc)
        Texts (including WhatsApp)
        Facebook/ other web-based platforms
        Other, specify
20. Has any staff member been trained on COVID19?
    a. How many current staff members have been trained on COVID19?
    b. Where did the training take place? On-site/ off-site/ virtual
    c. Who provided the training? MoH/ PSK/ KPA/ Other, specify
21. When you have questions about COVID19, who do you consult? [can select more than one]
    None/ don't consult
    MoH
    Pharmacy in-charge/ manager/ colleague (s)
    Med reps
    Nearby doctor
    Other, specify
22. What is your main source of COVID19 information? [select one]
    TV/ Radio
    Webinar
    Social media
    Google search/ internet
    Scientific journals
    Colleagues/ other pharmacy
    KPA/ PSK communications
    CME/ CPD session
    MoH communications
    Other, specify

<u>SECTION IV: INFECTION PREVENTION</u>

For peer review only - http://bmjopen.bmj.com/site/about/guidelines.xhtml

23. Which infection prevention measure(s) is your pharmacy using among staff? [can select more than one]
    - None
    - Physical distancing, describe
    - Temperature monitoring
    - Hand hygiene (handwashing, hand sanitizer, etc)
    - Disinfection of surfaces
    - Face masks
    - Goggles
    - Other, specify
24. Which infection control measure(s) is your pharmacy using among clients? [can select more than one]
    - None
    - Physical distancing, describe
    - Temperature monitoring before entering the pharmacy
    - Require clients to be wearing face masks before entering the pharmacy
    - Hand washing
    - Hand sanitizer
    - Use of non-touch payment methods, describe
    - Information materials and messages
    - Other, specify

    Which information materials and messages is your pharmacy providing to clients?
    - Posters/ banners
    - Leaflets/ pamphlets
    - Audio announcements
    - TV screen displays
    - Other, specify

25. Which method do staff members mainly use for cleaning their hands? [select one]
    - Handwashing
    - Hand sanitizer
    - Other, specify

    a. [If handwashing is main] In the last 5 working days,
        i. On how many days was water available for handwashing in your pharmacy? [enter value between 0 and 5]
        ii. On how many days was soap available for handwashing in your pharmacy? [enter value between 0 and 5]
    b. [If hand sanitizer is main] In the last 5 working days,
        i. On how many days was hand sanitizer available in your pharmacy? [enter value between 0 and 5]
26. What type of masks do staff use in your pharmacy? [can select more than one]
    - Cloth masks
    - Surgical/ medical masks
    - N95 or similar
    - Other, specify
27. Have staff members been trained on COVID19 infection prevention? Yes/no
    a. If yes, by who? [can select more than one]
        - MoH
        - Pharmacy in charge/ colleague
        - Other, specify
    b. Where did the training take place? [can select more than one]
        - Off-site, specify venue
        - In-person within the pharmacy

For peer review only - http://bmjopen.bmj.com/site/about/guidelines.xhtml

Remotely (virtually) when at the pharmacy
Remotely (virtually) when at home
Other, specify

28. Do you have any written guidelines on COVID19 infection prevention? Yes/no
    a. If yes, describe the guidelines you have access to (e.g. source, title, etc) [open-ended]
29. How adequate do you feel the infection prevention measures in your pharmacy are? [select one]
    Very inadequate/ inadequate/ adequate/ very adequate
30. Has any staff member in your pharmacy ever tested positive for COVID19? [yes/no]
    a. How many staff members have ever tested COVID19 positive? [numeric]
31. State any other comments you have regarding COVID19 infection prevention at your workplace? [open-ended, leave blank if none]

SECTION V: VIEWS ABOUT CORONAVIRUS 2019 (COV19) TESTING IN PHARMACIES

32. Do you get clients asking for COV19 testing? Yes/no
    a. In the last one week, how many clients asked for COV19 testing? [numeric]
    b. What help do you offer clients asking for COV19 testing? COV19 tests/ Temperature measurement/ Referral/ other
        i. If referral, where do you refer? [can select more than one]
            Another pharmacy
            Public clinic/ hospital
            Private clinic/ hospital
            Private lab
            Other, specify
33. [If providing COV19 testing] What type of tests do you provide? [can select more than one]
    Antibody rapid tests, specify [unit cost]
    Antigen rapid tests, specify [unit cost]
    Laboratory tests, specify [unit cost]
    Other, specify [unit cost]
34. [If not currently providing] Do you think COV19 testing should be offered in pharmacies? Yes/no
    a. If yes, state the main reason for supporting pharmacy-based COV19 testing [select one]
        There is demand for it/ clients ask for it
        Clients shy away from health facilities
        Clients perceive pharmacies as more confidential compared to other testing facilities
        Pharmacies are open for longer hours
        Pharmacies are easily accessible
        Pharmacies provide speedier (faster) services
        Other, specify
    b. If no, state the main reason for NOT supporting pharmacy-based COV19 testing
        There is no demand for it
        No privacy in pharmacies
        Might be misused
        Other, specify
35. State any other comments you have regarding provision of COV19 testing in retail pharmacies? [open-ended, leave blank if none]

SECTION VI: OTHER VIEWS AND EXPERIENCES

36. How prepared do you feel about handling a client who presents to the pharmacy with COVID19 symptoms? [select one]
    Very unprepared/ unprepared/ prepared/ very prepared
37. Which is the main concern you are facing related to personal health and wellbeing? [select one]
    None
    Exposure to infection at work

For peer review only - http://bmjopen.bmj.com/site/about/guidelines.xhtml

Lack of transportation to get to work
Being caught up with the curfew
Keeping my family safe
Meeting financial needs for me and my family
Other, specify

38. How much do you feel your personal role as a health worker in the COVID19 response is valued by the community you are serving?
    Not at all valued/ little valued/ highly valued/ very highly valued
39. What other inputs can pharmacies (uniquely) contribute to the national response to the epidemic?? [open-ended]
40. State any other comments you have regarding pharmacy practice during the COVID19 pandemic? [open-ended, leave blank if none]

For peer review only

For peer review only - http://bmjopen.bmj.com/site/about/guidelines.xhtml

Supplementary file B - Simulated client script

Call-in simulation

- Look up the pharmacy on Google Maps and note basic operational details, such as opening hours or COVID19-related client interactions. Take note of any COVID-related messaging.
- Call the customer service number on the Google Maps profile, or if no profile or no number provided, try a simple google search, otherwise contact the study coordinators. They will check if the number in the database is personal or official; if personal further discussion is needed to assess risk of detection.
- **Ask if they have medicines that you can use to protect yourself from getting COVID19, using appropriate language** as agreed during the training. Avoid prompting for information unless it is in the script.
- Make a mental note of **questions asked or advice given**. Encourage the provider to talk by listening actively and saying “don’t know” if the provider asks, for example, if you know how to prepare ginger home remedy.
  - If asked why you are asking for the medicines, say that your job requires interaction with customers, some of who don’t wear masks
  - If asked where you are located, mention a name of a place near the pharmacy
- If any products are recommended, ask and **write down the names.** You can also ask for the price to keep the conversation natural, but the price data is not being collected
- Ask the service provider **how you can get the medicines**. List down the **options given**, and then if “pick up from the pharmacy” is one of the options, mention that you would rather not visit the pharmacy physically, because you are afraid of getting exposed to COVID19.
- If not told spontaneously,
  - ask what **symptoms you should look out for** if you suspect COVID19 infection? If asked what symptoms you have, emphasize you don’t have symptoms but just want to be ready.
  - ask **what you should do if you suspect you have COVID19 infection**?
- Thank the service provider and mention that you will get back to them to arrange how to get the medicines.

Completing the debrief questionnaire

- The debrief questionnaire should ideally be completed within 30 minutes of the simulation, while memory is still fresh. In any case, do not call another pharmacy before the previous debrief interview has been done.
- It is **very important to say honestly how the call went**, even if you feel like it didn’t go very well. The **study is assessing the pharmacy and not you**, the simulated client.
- Try to capture the responses to open-ended questions close as possible to what the provider says (verbatim)
- If there is follow-up interaction, you can leave the questionnaire “unsubmitted” and finish later. Open a fresh page if you need to do another pharmacy
- If further information comes in after you have submitted, relay to the study coordinators who will then edit the record from the back end.

Ver 1.2; 08 dec 2020

For peer review only

Supplementary file C - debrief questionnaire

Basic data

- Pharmacy identifier
- Simulated client identifier
- Simulation type: in-person/ call-in

Call-in simulation

- What time did you call the pharmacy? [24-hour format]
- What was the gender of the person who served you? Male/ female/ could not tell
- Were any medicines recommended? Yes/no
- If yes, which medicines were recommended for prevention? [Indicate price for each]
  - None
  - Vitamin C
  - Zinc
  - Multivitamins
  - Azithromycin,
  - Other antibiotics (Augmentin, Amoxil, ceftriaxone, etc)
  - Herbal medicine, specify
  - Home remedies, specify
  - Other, specify
- What options were given on how you can get the recommended medicines?
  - None
  - Come to the pharmacy and pick
  - Send someone to the pharmacy to pick
  - The pharmacy to deliver to client's location
  - From nearest chemist/ health facility
  - Other, specify
- If no medicines recommended, what was recommended?
  - None
  - Mask use
  - Hand hygiene (washing, sanitizer)
  - Other, specify
- Were you asked why you are asking for the medicines? (yes/no)
- Before prompting, were you told the symptoms to look out for if you suspect COVID19 infection? Yes/no
  - If yes, describe [open-ended]
- After prompting, what symptoms were you told to look out for if you suspect COVID19 infection? [open-ended]
- Before prompting, were you told what to do if you suspect you have COVID19 infection? Yes/no
  - If yes, what was recommended if you suspect COVID19 infection?
    - Medications
    - Visit nearest health facility/ go see a doctor
    - Check for information online/ WHO website
    - Get tested for COVID19
    - Get checked for oxygen level
    - Get checked for body temperature
    - Self-quarantine/ isolate at home
    - other, specify
  - If medications, what medications were recommended for treatment?
    - Steriods (dexamethasone, betamethasone, prednisolone, etc)
    - Azithromycin,

Ver 1.3; 08 dec 2020

For peer review only - http://bmjopen.bmj.com/site/about/guidelines.xhtml

  - Other antibiotics (Augmentin, Amoxil, ceftriaxone, etc)
  - Painkillers (paracetamol, brufen, etc)
  - Herbal medicine, specify
  - Home remedies, specify
  - Other, specify
- After prompting, what were you told to do if you suspect you have COVID19 infection? Medications
  - Nothing
  - Visit nearest health facility/ go see a doctor
  - Check for information online/ WHO website
  - Get tested for COVID19
  - Get checked for oxygen level
  - Get checked for body temperature
  - Self-quarantine/ isolate at home
  - other, specify
  - If medications, what medications were recommended for treatment?
    - Steriods (dexamethasone, betamethasone, prednisolone, etc)
    - Azithromycin,
    - Other antibiotics (Augmentin, Amoxil, ceftriaxone, etc)
    - Painkillers (paracetamol, brufen, etc)
    - Herbal medicine, specify
    - Home remedies
    - Other, specify
- What other advice was given?
  - None
  - Wear a mask
  - Sanitize/ wash hands
  - Eat fruits
  - Other, specify
- What other questions were you asked? [open-ended]
- About how long was the call? [mins]
- Was there further interaction after the call? Yes/no
  - If yes, describe
    - SMS
    - Whatsapp
    - Follow-up call
    - Other, specify

<u>General observations</u>

- How friendly (approachable) was the person (s) who served you?
  1 – Very unfriendly
  2 – Unfriendly
  3 – Friendly
  4 – Very friendly
- How enthusiastic was the provider about providing the service?
  1 – Very unenthusiastic
  2 – Unenthusiastic
  3 – Enthusiastic
  4 – Very enthusiastic
- Did the provider say or do anything that you found particularly helpful or nice? Yes/no
  If yes, describe [free text]
- Did the provider say or do anything that you found particularly NOT helpful or NOT nice? Yes/no
  If yes, describe [free text]

Ver 1.3; 08 dec 2020

For peer review only

- Do you have any additional observations about the interaction? Yes/no

  If yes, describe [free text]

For peer review only

Ver 1.3; 08 dec 2020

Supplementary file D - In-depth Interview Topic Guide:

Assessing the response to the COVID-19 epidemic by private retail pharmacies in Kenya.

Notes to interviewer:

1. As first option, use Teams platform with video on. If internet is slow, try Teams without video and if that still doesn't work then use plain old telephone (POT).
2. If using POT, put the phone on speaker and record on a standard audio recorder placed next to the phone, and if using Teams, record directly through the application.
3. When setting up the interview, ask about internet access, preference of platform, etc. Inform the participant that they will need to be in a quiet place for about 30-60 minutes and that earphones are preferred to speaker mode.
4. Information required from the questionnaire:
    a. Preparedness level in handling COVID suspect case
    b. Products provided
    c. Information and counseling provided
    d. Whether they get clients who ask for testing
    e. Whether they think testing can be offered in a pharmacy

We would like to understand how the pharmacy sector is responding to the COVID19 epidemic, including: initial impact, experiences providing services during the epidemic, and views about pharmacy-based COVID19 testing. Towards the end of the interview I will also ask you a few questions about your experience being interviewed remotely compared to face-to-face.

Interviewee profile

1. Briefly tell me about your pharmacy (setting, staff, clients)?
    - What is your role in the pharmacy?

Initial response and impact

2. When did you first hear about COVID19? From who?
    - What did you think and feel about it at that time?

3. What was the initial response at your pharmacy?

4. What was the effect of the epidemic on pharmacy operations?
    - Client flow, sales, working hours, staff, meeting routine, etc

5. What has been your role in responding to the epidemic?
    - You said your preparedness in handling a COVID-19 case was: ___________________. Please explain further
    - What aspects of your education and previous experience helped you in responding to the outbreak?

COVID-related products and services

6. In the questionnaire, you mentioned a number of products that your pharmacy is providing in relation to the epidemic. ____
Which of these products were introduced specifically because of the epidemic?
    - You also mentioned information and counseling that clients are seeking or you are providing? ____
    - How is this done?
    - How does your pharmacy handle suspected COVID19 cases?

7. Have you been involved in any COVID training or peer learning sessions, either as a participant or a trainer?

Version 1.4; 19feb2021

For peer review only - http://bmjopen.bmj.com/site/about/guidelines.xhtml

- If yes, Where? When? Who else was included?
  - Was it stand alone training on this topic or part of a wider training?
  - Do you feel the training was adequate?
  - Who was supporting the training (financially and technically)?
- If no, how did you learn how to provide COVID services and products?

8. What do you think is needed to improve the delivery of COVID-related services in the retail pharmacy sector?
   - What support (material or financial) can be given by the government or any other external party? (e.g., drugs and other supplies/ toolkits/ job-aids/ digital systems)
   - Do you report information on COVID-related services to the government or any other external party?

Pharmacy-based COVID19 testing

9. If they get clients who ask for COVID19 testing: YES NO (*circle one*)
   - What type of clients ask for testing? (reasons for wanting to test, etc)
   - How do you help clients asking for COVID19 testing?
   - Why do you think they prefer pharmacies rather than health facilities or other testing sites?

10. You mentioned that COVID19 testing SHOULD/ SHOULD NOT *(circle one)* be provided in pharmacies? Explain.
    - What would be needed to ensure successful implementation of COV19 testing in the retail pharmacy sector?
    - What type of tests would be most suited for pharmacy-based COVID19 testing?

Other potential inputs into the response

11. What other inputs can pharmacies (uniquely) contribute to the national response to the epidemic?
    - How would this be done?

Conclusion of main interview: We have come to the end of the specific questions I had. Do you have any further comments?

Experience doing the interview remotely

Now I would like to ask you a few questions about your experience being interviewed remotely compared to face-to-face.

12. How would you describe your experience doing the interview remotely, compared to face-to-face?
    - Have you done a face-to-face interview before? Describe.
    - Advantages and challenges
    - Would you have preferred a face-to-face interview? Explain.

13. What did you like or not like about the Teams platform?
    - Do you have previous experience with digital meeting applications? (Teams, Zoom, etc)
    - Would you have preferred another platform?

Conclusion: Thank you very much for your time. If it is ok with you, I may contact you again for any clarifications or to hear about additional experiences and views on the topic. We will share a report once data analysis is complete.

Version 1.4; 19feb2021

For peer review only

For peer review only - http://bmjopen.bmj.com/site/about/guidelines.xhtml

Supplementary file E - Checklist – Consolidated criteria for reporting qualitative studies (COREQ)[i]

Manuscript: Response to the coronavirus disease 2019 (COVID-19) pandemic at private retail pharmacies in Kenya: a mixed methods study

Item number	Guide questions/description	Reported on Page #
**Domain 1: Research team and reflexivity**		
Personal Characteristics		
1. Inter viewer/facilitator	Which author/s conducted the interview or focus group?	4
2. Credentials	What were the researcher's credentials? E.g. PhD, MD	The names and affiliations are included on title page
3. Occupation	What was their occupation at the time of the study?	The names and affiliations are included on title page
4. Gender	Was the researcher male or female?	One female, one male
5. Experience and training	What experience or training did the researcher have?	The names and affiliations are included on title page
Relationship with participants		
6. Relationship established	Was a relationship established prior to study commencement?	4- Participants had been included in a previous study
7. Participant knowledge of the interviewer	What did the participants know about the researcher? e.g. personal goals, reasons for doing the research	5 – participants signed a consent form that explained the research goals
8. Interviewer characteristics	What characteristics were reported about the inter viewer/facilitator? e.g. Bias, assumptions, reasons and interests in the research topic	No bias to report
**Domain 2: study design**		
Theoretical framework		
9. Methodological orientation and Theory	What methodological orientation was stated to underpin the study? e.g. grounded theory, discourse analysis, ethnography, phenomenology, content analysis	5- thematic analysis
Participant selection		
10. Sampling	How were participants selected? e.g. purposive, convenience, consecutive, snowball	4- purposive
11. Method of approach	How were participants approached? e.g. face-to-face, telephone, mail, email	4- telephone
12. Sample size	How many participants were in the study?	5 – 18 participants
13. Non-participation	How many people refused to participate or dropped out? Reasons?	Participatin rate was 56% of 32 providers invited; 7 of those invited did not respond after three reminders, 5 said they were not interested, and 2 had already left the pharmacy

For peer review only

Item number	Guide questions/description	Reported on Page #
		Not included due to word limit
Setting		
14. Setting of data collection	Where was the data collected? e.g. home, clinic, workplace	4-phone
15. Presence of non-participants	Was anyone else present besides the participants and researchers?	No.<br><br>*Not included due to word limit*
16. Description of sample	What are the important characteristics of the sample? e.g. demographic data, date	5 – supp tab 2
Data collection		
17. Interview guide	Were questions, prompts, guides provided by the authors? Was it pilot tested?	4 – supp file D<br><br>The interview guide was updated after the first few interviews based on responses from participants and how effectively these addressed research objectives.<br><br>*Not included due to word limit*
18. Repeat interviews	Were repeat interviews carried out? If yes, how many?	No repeat interviews were done.<br><br>*Not included due to word limit*
19. Audio/visual recording	Did the research use audio or visual recording to collect the data?	5 – data management and analysis
20. Field notes	Were field notes made during and/or after the interview or focus group?	Field notes made after the interviews<br><br>*Not included due to word limit*
21. Duration	What was the duration of the interviews or focus group?	Interviews lasted a median [range] of 35 [20-92] minutes<br><br>*Not included due to word limit*
22. Data saturation	Was data saturation discussed?	To expedite data collection, all 18 interviews were completed before starting analysis or checking for saturation. During analysis, Saturation was reached after 14 interviews, but all 18 interviews were included in analysis.

For peer review only

Item number	Guide questions/description	Reported on Page #
		Not included due to word limit
23. Transcripts returned	Were transcripts returned to participants for comment and/or correction?	Transcripts were not returned to participants for comment and/or correction *Not included due to word limit*
**Domain 3: analysis and findings**		
Data analysis		
24. Number of data coders	How many data coders coded the data?	5 – data management and analysis
25. Description of the coding tree	Did authors provide a description of the coding tree?	No.<br><br>*Not included due to word limit*
26. Derivation of themes	Were themes identified in advance or derived from the data?	Analysis followed a thematic approach with initial categories based on the interview guide and emergent themes integrated<br><br>*Not included due to word limit*
27. Software	What software, if applicable, was used to manage the data?	5- NVivo 12
28. Participant checking	Did participants provide feedback on the findings?	Participants were not asked for feedback on the findings.<br><br>*Not included due to word limit*
Reporting		
29. Quotations presented	Were participant quotations presented to illustrate the themes/findings? Was each quotation identified? e.g. participant number	Yes. Results section
30. Data and findings consistent	Was there consistency between the data presented and the findings?	Yes.
31. Clarity of major themes	Were major themes clearly presented in the findings?	Yes. Results section
32. Clarity of minor themes	Is there a description of diverse cases or discussion of minor themes?	Yes. Results section

[i] Developed from:
Tong A, Sainsbury P, Craig J. Consolidated criteria for reporting qualitative research (COREQ): a 32-item checklist for interviews and focus groups. *International Journal for Quality in Health Care*. 2007. Volume 19, Number 6: pp. 349 – 357

For peer review only

STROBE Statement—Checklist of items that should be included in reports of ***cross-sectional studies***

	Item No	Recommendation	Page No
**Title and abstract**	1	(*a*) Indicate the study's design with a commonly used term in the title or the abstract	1, 2
		(*b*) Provide in the abstract an informative and balanced summary of what was done and what was found	2
**Introduction**			
Background/rationale	2	Explain the scientific background and rationale for the investigation being reported	2-3
Objectives	3	State specific objectives, including any prespecified hypotheses	3
**Methods**			
Study design	4	Present key elements of study design early in the paper	3
Setting	5	Describe the setting, locations, and relevant dates, including periods of recruitment, exposure, follow-up, and data collection	3
Participants	6	(*a*) Give the eligibility criteria, and the sources and methods of selection of participants	4
Variables	7	Clearly define all outcomes, exposures, predictors, potential confounders, and effect modifiers. Give diagnostic criteria, if applicable	4
Data sources/ measurement	8*	For each variable of interest, give sources of data and details of methods of assessment (measurement). Describe comparability of assessment methods if there is more than one group	*4*
Bias	9	Describe any efforts to address potential sources of bias	4-5
Study size	10	Explain how the study size was arrived at	4
Quantitative variables	11	Explain how quantitative variables were handled in the analyses. If applicable, describe which groupings were chosen and why	4-5
Statistical methods	12	(*a*) Describe all statistical methods, including those used to control for confounding	5
		(*b*) Describe any methods used to examine subgroups and interactions	5
		(*c*) Explain how missing data were addressed	5
		(*d*) If applicable, describe analytical methods taking account of sampling strategy	5
		(*e*) Describe any sensitivity analyses	5
**Results**			
Participants	13*	(a) Report numbers of individuals at each stage of study—eg numbers potentially eligible, examined for eligibility, confirmed eligible, included in the study, completing follow-up, and analysed	5
		(b) Give reasons for non-participation at each stage	5
		(c) Consider use of a flow diagram	n/a
Descriptive data	14*	(a) Give characteristics of study participants (eg demographic, clinical, social) and information on exposures and potential confounders	5
		(b) Indicate number of participants with missing data for each variable of interest	5
Outcome data	15*	Report numbers of outcome events or summary measures	5
Main results	16	(*a*) Give unadjusted estimates and, if applicable, confounder-adjusted estimates and their precision (eg, 95% confidence interval). Make clear which confounders were adjusted for and why they were included	6-17

For peer review only - http://bmjopen.bmj.com/site/about/guidelines.xhtml

For peer review only

		(*b*) Report category boundaries when continuous variables were categorized	6-17
		(*c*) If relevant, consider translating estimates of relative risk into absolute risk for a meaningful time period	6-17
Other analyses	17	Report other analyses done—eg analyses of subgroups and interactions, and sensitivity analyses	6-17
**Discussion**			
Key results	18	Summarise key results with reference to study objectives	17-20
Limitations	19	Discuss limitations of the study, taking into account sources of potential bias or imprecision. Discuss both direction and magnitude of any potential bias	20
Interpretation	20	Give a cautious overall interpretation of results considering objectives, limitations, multiplicity of analyses, results from similar studies, and other relevant evidence	17-20
Generalisability	21	Discuss the generalisability (external validity) of the study results	20
**Other information**			
Funding	22	Give the source of funding and the role of the funders for the present study and, if applicable, for the original study on which the present article is based	20-21

*Give information separately for exposed and unexposed groups.

Note: An Explanation and Elaboration article discusses each checklist item and gives methodological background and published examples of transparent reporting. The STROBE checklist is best used in conjunction with this article (freely available on the Web sites of PLoS Medicine at http://www.plosmedicine.org/, Annals of Internal Medicine at http://www.annals.org/, and Epidemiology at http://www.epidem.com/). Information on the STROBE Initiative is available at www.strobe-statement.org.

For peer review only

For peer review only - http://bmjopen.bmj.com/site/about/guidelines.xhtml
